# Self-supplying hydrogen peroxide-driven gold nanoparticle aggregation via bio-orthogonal click reaction activates cGAS-STING-PERK pathway for multimodal tumor therapy

**DOI:** 10.1186/s12951-026-04741-3

**Published:** 2026-06-27

**Authors:** Junyou LI, Kaiqi Hu, Man Lung Lee, Ting Li, Pinyou Chen, Chengke Wang, Hung-Wing LI

**Affiliations:** 1 Department of Chemistry, The Chinese University of Hong Kong, New Territories, 000000 Hong Kong China; 2 Department of Food Science, Ludong University, Yantai, 264000 Shan Dong Province China

**Keywords:** Type I photodynamic therapy, Fenton-like reaction, Self-supplying H_2_O_2_, Bio-orthogonal tetrazole/alkene cycloaddition, Mitochondria targeting, Endoplasmic reticulum stress

## Abstract

We developed an effective nanoplatform, _T+EP_AuNP_Lu&Lap_, that concurrently harnesses Type I photodynamic therapy (PDT), chemodynamic therapy (CDT), photothermal therapy (PTT), and immunomodulation to remodel the immunosuppressive tumor microenvironment (TME). By pairing PpIX and EGCG, the system enables the transformation of PpIX from Type II PDT to oxygen-independent Type I for superoxide (O_2_•^–^) generation, courtesy of electron-richness and low reduction potential of EGCG. Mn^2+^-mediated Fenton-like reaction produces hydroxyl radicals (•OH) for CDT, while its superoxide dismutase (SOD) activity alleviates tumor hypoxia. β-Lapachone-triggered H_2_O_2_ elevation augments luminol chemiluminescence, which not merely fortifies CDT, but also induces bio-orthogonal tetrazole-PpIX click reaction, driving AuNP aggregation for deep-tissue PTT without the need of external light. EGCG-mediated mitochondrial targeting intensifies mitochondrial oxidative and thermal stress, further amplified by EQ-driven GSH depletion. This causes ATP depletion, HSP70 downregulation, ICD, and mtDNA release, which activates the cGAS-STING-PERK pathway, promotes ER stress and antitumor immunity, and inhibits lung metastasis. Collectively, this multimodal nanoplatform remodels TME into a hypoxia-alleviated, M1 macrophage-rich, and Treg-suppressed proinflammatory state, achieving efficacious eradication of tumor and inhibition of pulmonary metastasis in vivo.

**Graphical Abstract:**

**Figure:**
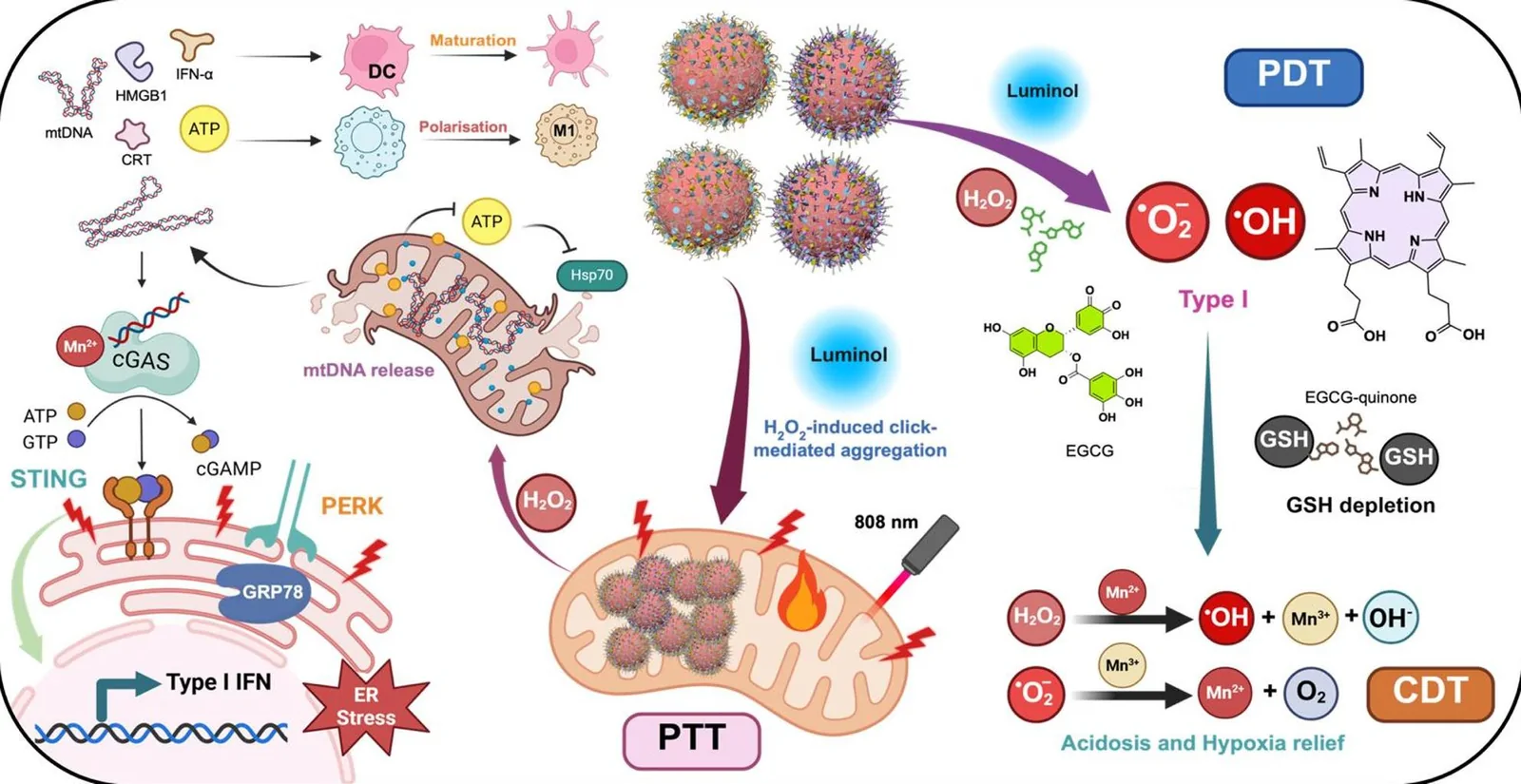
Self-Supplying Hydrogen Peroxide-Driven Gold Nanopar-ticle Aggregation via Bio-orthogonal Click Reaction Acti-vates cGAS-STING-PERK Pathway for Multimodal Tu-mor Therapy

**Supplementary Information:**

The online version contains supplementary material available at https://doi.org/10.1186/s12951-026-04741-3.

## Introduction

**Fig. 1:**
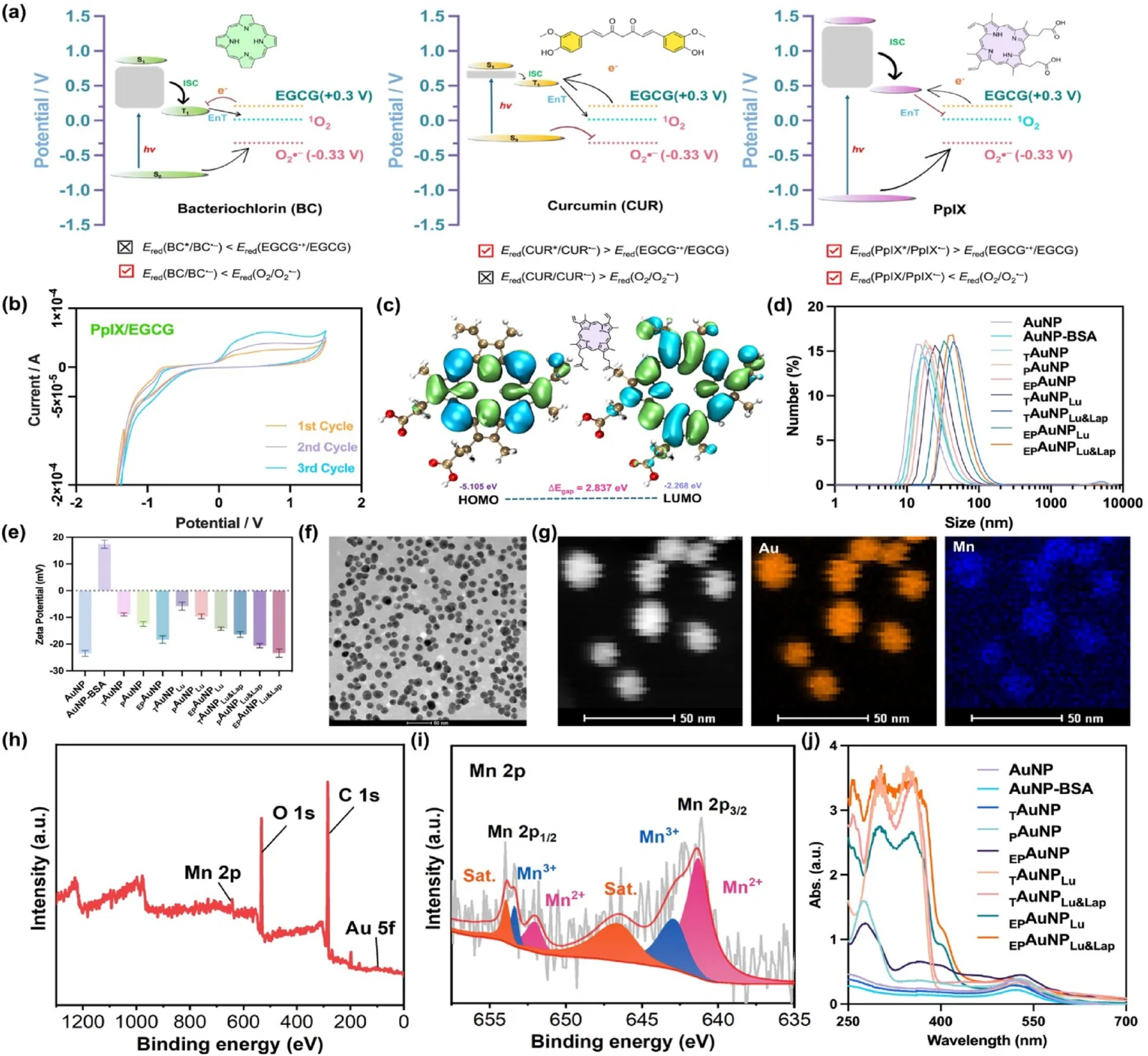
Characterizations of _T+EP_AuNP_Lu&Lap_. (**a**) Jablonski diagram of Type I and Type II photosensitization processes based on electrochemical potential energy levels. Left: BC as a paradigm of Type II-dominated PSs with large Δ*E*_ST_ and a high reduction potential in the triplet excited state, yet with a too low reduction potential in the triplet excited state to gain electrons from the substrate (EGCG as an electron substrate). Middle: CUR as a showcase for Type II-dominated PSs with small Δ*E*_ST_ and a low reduction potential in the ground state, yet with a too high reduction potential in the ground state. Right: the transformation of PpIX from conventional Type II-dominated PS to Type I-dominated PS with the addition of EGCG, with a low enough reduction potential in the ground state and a high enough reduction potential in the triplet excited state. (**b**) CV spectra of PpIX with the addition of EGCG. (**c**) HOMO-LUMO structure of PpIX with energy level. (**d**) DLS analysis, and (**e**) zeta potentials of progressive synthesis of _T+EP_AuNP_Lu&Lap_ and its counterparts. (**f**) TEM images, (**g**) elemental mapping, (**h**) XPS spectra, and (**i**) Mn 2p XPS spectra of _T+EP_AuNP_Lu&Lap_. (**j**) UV-Vis absorbance of progressive synthesis of _T+EP_AuNP_Lu&Lap_ and its counterparts. Quantitative data are presented as mean ± SD (*n* = 3)

 Photodynamic therapy (PDT) has gained mounting attention for its spatiotemporal precision and minimal invasiveness [1]. However, conventional Type II PDT is severely hindered by a strict reliance on molecular oxygen, limiting its efficacy in deep-seated, hypoxic tumor microenvironments (TME) [2, 3]. Type I PDT offers an alternative that functions effectively under low-oxygen conditions [4, 5]. In contrast to Type II PDT, Type I PDT proceeds through electron or hydrogen transfer between an excited photosensitizer and nearby biomolecules to generate radicals, including O2•– and •OH. In this process, oxygen mainly functions as a redox mediator rather than a stoichiometric reactant [6, 7]. However, efficient Type I PDT requires stringent electrochemical matching. Specifically, the ground-state reduction potential of the PS (*E*_red_(PS*/PS•^–^)) must be lower than the oxygen reduction threshold *E*_red_(O_2_/O_2_•^–^) = −0.33 V to efficiently drive radical generation [8, 9]. In a rigorous screening, we identified Protoporphyrin IX (PpIX), an FDA-approved compound, as a thermodynamically ideal candidate [10, 11]. With a ground-state potential of approximately −1.10 V, as illustrated in Fig. 1a, PpIX easily surpasses the threshold required to reduce O_2_ to O_2_•^–^, a capability lacking in other common PSs like bacteriochlorin or curcumin [12, 13]. To fuel this redox cycle, we introduced Epigallocatechin-3-*O*-gallate (EGCG) as a superior electron donor [14]. EGCG possesses abundant hydroxyl groups and a low reduction potential (approximately 0.3 V), allowing the excited PpIX (*E*_red_(PpIX/PpIX•^–^) = 0.67 V) to efficiently capture electrons from EGCG [15–18]. Furthermore, the oxidation of EGCG to its quinone (EQ) consumes glutathion**e** (GSH), thereby further amplifying oxidative stress. This rational pairing of PpIX and EGCG establishes a potent, hypoxia-tolerant ROS generating system** [**15, 19, 20**]. **

**Scheme 1:**
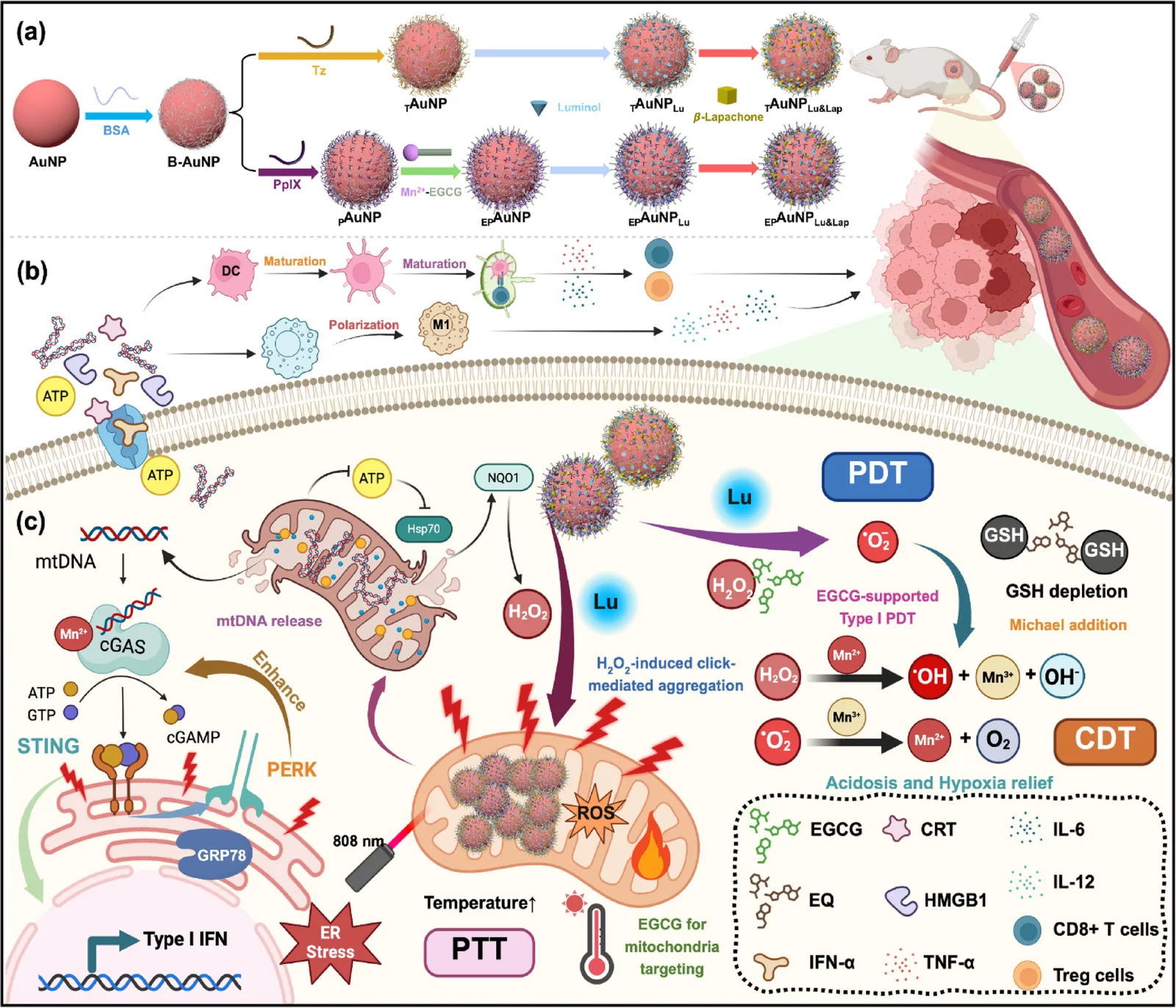
Illustration of comprehensive and holistic therapeutic modalities of _T+EP_AuNP_Lu&Lap_ that integrates reciprocal PDT, CDT, PTT, and immunotherapy. **a**) Preparation and synthesis route of _T+EP_AuNP_Lu&Lap_. **b**) Immunotherapy via M1 TAMs polarization and DC maturation for pro-inflammatory cytokines release, CD8^+^ T cell activation and Treg cells suppression. **c**) Mutually augmentation of cGAS-STING pathway and ROS/heat elicited ER stress, as well as combination of CDT, PDT and PTT for holistic tumor therapy

 While Type I PDT can overcome the hypoxia barrier by using oxygen merely as a redox mediator to generate radicals, it still requires external light activation. To address these challenges, we engineered a self-illuminating, hypoxia-tolerant nanoplatform centered around a rationally designed electron-transfer pair (Scheme 1). We identified Protoporphyrin IX (PpIX) as an ideal photosensitizer with a ground-state potential low enough to efficiently drive radical generation. By pairing PpIX with Epigallocatechin-3-*O*-gallate (EGCG) as a robust electron donor, we established a highly potent, hypoxia-tolerant reactive oxygen species (ROS) generator. Crucially, to bypass the need for external light, we additionally integrated luminol (Lu), which emits endogenous blue light that directly overlaps with the absorption profile of PpIX (Figure S2) [21–23].

This internal chemiluminescence not only activates deep-tissue PDT but also serves as the trigger for the subsequent core tactic: targeted therapeutic aggregation. Gold nanoparticles (AuNP) are classical and well-established materials in PTT application due to their excellent biocompatibility, tunable optical absorption, and strong localized surface plasmon resonance (LSPR) effect. However, their therapeutic performance is restricted by a size trade-off: small AuNPs penetrate tumors efficiently but are rapidly cleared, whereas larger particles exhibit longer circulation but limited tissue diffusion [24–27]. To resolve this issue, we developed a size-transformable AuNP system that remains small and monodisperse during circulation for deep tumor penetration while undergoes controlled aggregation inside tumor cells [28]. We implemented this strategy by leveraging a bio-orthogonal photo-click chemistry reaction and capitalized on the previously overlooked intrinsic alkene group existing within the PpIX molecule [29]. By conjugating a 2,5-diphenyltetrazole (Tz) moiety onto the surface of AuNPs, we created a system where the tetrazole group can undergo a highly specific, Lu chemiluminescence-triggered cycloaddition with PpIX molecules. This reaction successfully “clicks” the small, deeply penetrating AuNPs together directly inside the tumor cells upon luminescence, inducing precise intracellular aggregation to maximize both tumor retention and PTT efficiency.

To ensure the sustained operation of this primary phototheranostic axis, the nanoplatform (termed _T+EP_AuNP_Lu+Lap_) incorporates specific metabolic amplifiers. β-lapachone (Lap) is included to continuously boost intracellular otherwise insufficient H_2_O_2_ levels [30, 31], fueling the luminol luminescence required for PpIX activation [32–34]. Furthermore, Mn^2+^ is integrated to provide supportive chemodynamic therapy (CDT) via a superoxide dismutase-like (SOD-like) mechanism [35–37]. In traditional Mn-based CDT, the conversion of Mn^3+^ back to Mn^2+^ via H_2_O_2_ is notoriously slow, severely throttling •OH generation and acting as a severe rate-limiting bottleneck that throttles the continuous production of highly toxic •OH [38, 39]. Our platform couples this process with Type I PDT. The O_2_•^–^ generated by the Type I PDT promote the continuous, rapid reduction of Mn^3+^ back to Mn^2+^ via a superoxide SOD-like mechanism, effectively bypassing this traditional limitation, serving as a dominant contributor. Moreover, the Lap-mediated H_2_O_2_ elevation further facilitates Mn^2+^ to Mn^3+^, serving as another dominant contributor to address the traditionally slow catalytic bottleneck of Mn-based CDT system.

$$\:{\mathrm{Mn}}^{\mathrm{2+}}\mathrm{+}{\mathrm{H}}_{\mathrm{2}}{\mathrm{O}}_{\mathrm{2}}\rightarrow{\mathrm{Mn}}^{\mathrm{3+}}\mathrm{+}{\mathrm{OH}}^{\mathrm{-}}\mathrm{+}\mathrm{.}\mathrm{OH}$$

$$\:{\mathrm{(Traditional:Mn}}^{\mathrm{3+}}\mathrm{+}{\mathrm{H}}_{\mathrm{2}}{\mathrm{O}}_{\mathrm{2}}\underrightarrow{\text{Very slow}}{\mathrm{Mn}}^{\mathrm{2+}}\mathrm{+}{\mathrm{O}}_{\mathrm{2}}\mathrm{+}{\mathrm{H}}^{\mathrm{+}})$$

$$\:\text{Our design:}{\mathrm{Mn}}^{\mathrm{3+}}\mathrm{+}{{\mathrm{O}}_{\mathrm{2}}\mathrm{.}}^{\mathrm{-}}\underrightarrow{\mathrm{SOD-like}}\text{}{\mathrm{Mn}}^{\mathrm{2+}}\mathrm{+}{\mathrm{O}}_{\mathrm{2}}$$

Ultimately, the profound mitochondrial and endoplasmic reticulum stress generated by this primary photothermal and photochemical damage leads to a definitive downstream cascade. The resultant release of mitochondrial DNA, sensitized by the presence of Mn^2+^ ions, strongly activates the cGAS-STING pathway. This innate immune activation promotes Type I interferon production and ICD. It converts local therapy into systemic immunity that suppresses metastasis, enhances CD8^+^ T cell activity, and reduces Treg-mediated immunosuppression to create an immunostimulatory milieu [40–42].

## Results and discussion

### Preparation and Type I PDT ability of _EP_AuNP_Lu&Lap_

 The addition of EGCG was demonstrated to transform PpIX into Type I PDT, as supported by cyclic voltammetry (CV) analysis. As illustrated in Fig. 1b, the ground-state reduction potential of PpIX/EGCG was notably lower compared to PpIX alone (Figure S3), indicating a significant enhancement in redox activity. As suggested in Table S1, PpIX/EGCG is more receptive to gaining electrons compared to PpIX alone (cathodic peak potential from −1.01 to −0.93). For the anodic process, pure PpIX required a higher potential (+0.75 V) to lose electrons. After EGCG addition, the anodic peak shifted to +0.68 V, indicating that PpIX/EGCG more readily donates electrons and forms radicals. Additionally, the HOMO-LUMO structure of PpIX/EGCG revealed a narrow energy gap of 2.837 eV, signifying less energy is required for the molecule to transition from the ground state to the excited state (Fig. 1c). Subsequently, AuNP was modification with HS-mPEG and BSA to produce AuNP-BSA. Tz and PpIX were then functionalized onto the AuNP surface via amidation to yield _T_AuNP and _P_AuNP. Mn^2+^ was then chelated on _P_AuNP, followed by the addition of EGCG to obtain _EP_AuNP. Further modification with Lu and Lap was achieved through electrostatic interactions and π-π stacking, resulting in the final nanoplatforms: _T_AuNP_Lu&Lap_ and _EP_AuNP_Lu&Lap_. Dynamic light scattering (DLS) analysis revealed that the gradually increased size of the synthesized AuNP from 13 nm to around 55 nm, reflecting the successful surface modifications. (Fig. 1 d). Transmission electron microscopy (TEM) analysis (Fig. 1f) indicated monodispersed 13 nm size of _EP_AuNP_Lu&Lap_ and its counterparts (Figure S4). In addition, Fourier Transform Infrared spectroscopy (FTIR) in Figure S5 exhibited relevant peaks of the product at each synthetic step. Moreover, the hydrodynamic sizes of AuNP analyzed by DLS remained nearly unchanged in 10% FBS at room temperature over 14 days, highlighting its excellent colloidal stability and suitability for biological applications (Figure S6). Zeta potentials also indicated corresponding changes after respective modifications (Fig. 1e). Elemental mapping results (Fig. 1 g) manifested the successful conjugation of Mn^2+^, further supported by X-ray photoelectron spectroscopy (XPS) spectra (Fig. 1 h). The high-resolution spectrum of Mn 2p shown in Fig. 1i confirmed the presence of mixed-valence manganese (Mn^2+^/Mn^3+^) at 640.9 eV and 642.5 eV, proving the tangibility and feasibility of Mn^2+^-mediated CDT. UV-Vis spectra additionally indicated successful modification of the functional moieties onto AuNP surface (Fig. 1j). The loading amounts of Lu and Lap were determined using UV-Vis spectroscopy. Calibration curves at 257 nm for Lap and 365 nm for Lu (Figures S7 and S8) gave EE values of 66.1% and 58.2%, respectively, and LC values of 39.7% and 36.8%, respectively, confirming efficient loading of both agents.

### Evaluation of enhanced ROS generation ability

Tumor cells evade oxidative damage and therapeutic intervention by upregulating powerful, interconnected antioxidant defense systems, including high intracellular GSH concentration and expression of transcription factor Nrf2 that orchestrates a broad cytoprotective gene repertoire [43]. This robust redox buffering capacity effectively neutralizes ROS, thereby creating a major barrier to treatments that severely compromises therapeutic efficacy. To overcome this redox resistance, we engineered _T+EP_AuNP_Lu&Lap_ as a coordinated, self-reinforcing platform that disrupts tumor redox homeostasis and triggers synergistic reactions to overwhelm cellular defenses.

**Fig. 2:**
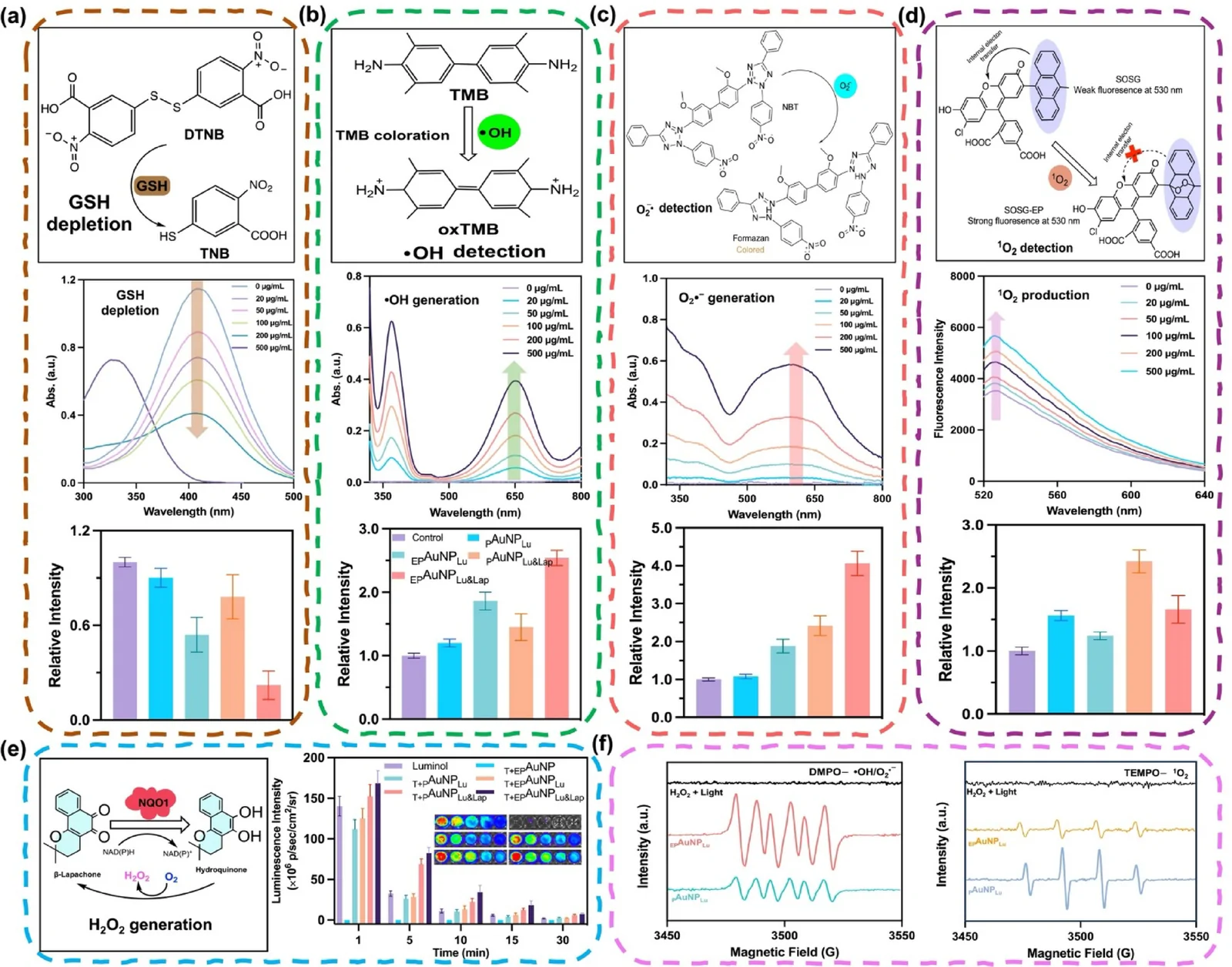
Assessment of ROS generation ability of _T+EP_AuNP_Lu&Lap_. (**a**) GSH depletion ability using DTNB, (**b**) •OH generation ability via TMB oxidization, (**c**) O_2_^•^⁻ production ability by NBT, and (**d**) ^1^O_2_ yielding ability using SOSG of _EP_AuNP_Lu&Lap_. Mechanism (upmost), concentration-dependent detection spectra (middle), and comparison among _EP_AuNP_Lu&Lap_ and its counterparts (bottom) at a concentration of 200 µg mL^− 1^. (**e**) H_2_O_2_ generation ability in the presence of NQO1 and NADH enzyme in Tris-HCl buffer. Mechanism (left) and time-dependent H_2_O_2_-induced Lu luminescence intensity comparison among _T+EP_AuNP_Lu&Lap_ and its counterparts (right). (**f**) ESR spectra of _EP_AuNP_Lu_ and _P_AuNP_Lu_ for the detection of •OH and O_2_^•^⁻ via 5, 5-dimethyl-1-pyrroline N-oxide (DMPO, left) and ^1^O_2_ via 2,2,6,6-tetramethylpiperidine-1-oxyl (TEMPO, right). Quantitative data are presented as mean ± SD (*n* = 3)

ROS oxidize EGCG to EQ (Figure S9), which depletes GSH through Michael addition. GSH consumption was quantified using DTNB, which reacts with GSH to produce a yellow product with an absorption peak at 412 nm [44]. The results revealed that _EP_AuNP_Lu&Lap_ induced a progressive decrease in absorbance with increasing NP concentrations and incubation time, confirming substantial GSH consumption. In contrast, _P_AuNP_Lu(&Lap)_ exhibited only slight GSH depletion (Fig. 2a and S10a).

_EP_AuNP_Lu&Lap_ generates highly cytotoxic •OH via CDT, and its catalytic activity was assessed via 3,3’,5,5’-tetramethylbenzidine (TMB) oxidation [45]. The intensity of the oxTMB absorbance at 650 nm increased proportionally with _EP_AuNP_Lu&Lap_ concentration, confirming •OH production, while _P_AuNP_Lu&Lap_ showed limited •OH yielding ability (Fig. 2b and S10b). Nitroblue tetrazolium (NBT) was used to evaluate O_2_•^–^ production, as its reduction to formazan exhibits an absorbance peak at 560 nm [46]. _EP_AuNP_Lu&Lap_ displayed potent O_2_•^–^ generation ability, compared to _P_AuNP_Lu(&Lap),_ confirming the activation of the Type I PDT pathway in the presence of EGCG (Fig. 2c and S10c). In contrast, the ^1^O_2_ production of _P_AuNP_Lu(&Lap)_ was significantly higher than _EP_AuNP_Lu(&Lap)_, due to the dominant Type II pathway, detected by SOSG (Fig. 2d and S10d) [47]. 

Finally, the production of H_2_O_2_ was evaluated by using a H_2_O_2_ detection kit after adding NQO1/NADH enzymes. The results shown in Figure S11 disclosed that both _P_AuNP_Lu&Lap_ and _EP_AuNP_Lu&Lap_ produced large amount of H_2_O_2_, whereas _EP_AuNP_Lu_ produced a small amount and _P_AuNP_Lu_ showed only a negligible amount.

Subsequently, Lu chemiluminescence measurement was performed by incubating AuNP with 100 mM H_2_O_2_ and enzymes. As shown in Figure S12a and S12b, _T+EP_AuNP_Lu&Lap_ exhibited strong blue luminescence, with a fluorescence spectrum identical to that of Lu with pH-dependent enhancement. Importantly, _T+(E)P_AuNP_Lu&Lap_ showed slower luminescence decay compared to Lu alone over 30 min (Figure 2e). However, no detectable signal was detected from the aqueous solution of _T+EP_AuNP without Lu, confirming successful Lu incorporation. Besides, the luminescence was substantially enhanced with the increasing concentrations of _T+EP_AuNP_Lu&Lap_ (Figure S12c). Collectively, these results confirmed the capability of _T+EP_AuNP_Lu&Lap_ generating intense luminescence in the presence of H_2_O_2_ and enzyme in vitro.

Electron spin resonance (ESR) spectroscopy further validated the transformation to type I dominated mechanism of _EP_AuNP_Lu&Lap_ by elevated generation of O_2_•^–^, whereas _P_AuNP_Lu&Lap_ showed the type II-dominated mechanism by prominent ^1^O_2_ production (Figure 2f). To demonstrate that the system acts as a cascaded electron-transfer network rather than a simple synergistic co-administration, we evaluated ESR for PpIX alone and a simple solution mixture of PpIX and EGCG in a mixing cell. As illustrated in Figure S13, the resulting ESR spectra exhibited strong, characteristic signals of the TEMP-^1^O_2_ adduct. Conversely, the signals corresponding to DMPO- O_2_^•–^/•OH adducts were negligible, very similar to the control group where PpIX was irradiated alone. EGCG did not alter the ROS profile in simple co-administration, indicating that mixing alone is insufficient. Efficient Type II-to-Type I conversion requires close spatial confinement on the AuNP surface, which enables direct electron transfer from EGCG to PpIX.

Sequential electron-transfer and energy-conversion events inevitably incur intrinsic efficiency losses. In Type I PDT, efficiency loss often occurs via spontaneous recombination or non-radiative decay when a photosensitizer cannot readily secure an electron from its surroundings. The internal energy transfer from luminol chemiluminescence to PpIX activation, and subsequently to Tz-PpIX cycloaddition inherently incurs some energy loss. The most significant competing side reaction in any ROS generating cascade is the rapid scavenging of free radicals by the tumor’s robust antioxidant high concentration of GSH, necessitating the addition of EGCG to deplete intracellular GSH.

### Light-induced aggregation of AuNP for PTT

**Fig. 3:**
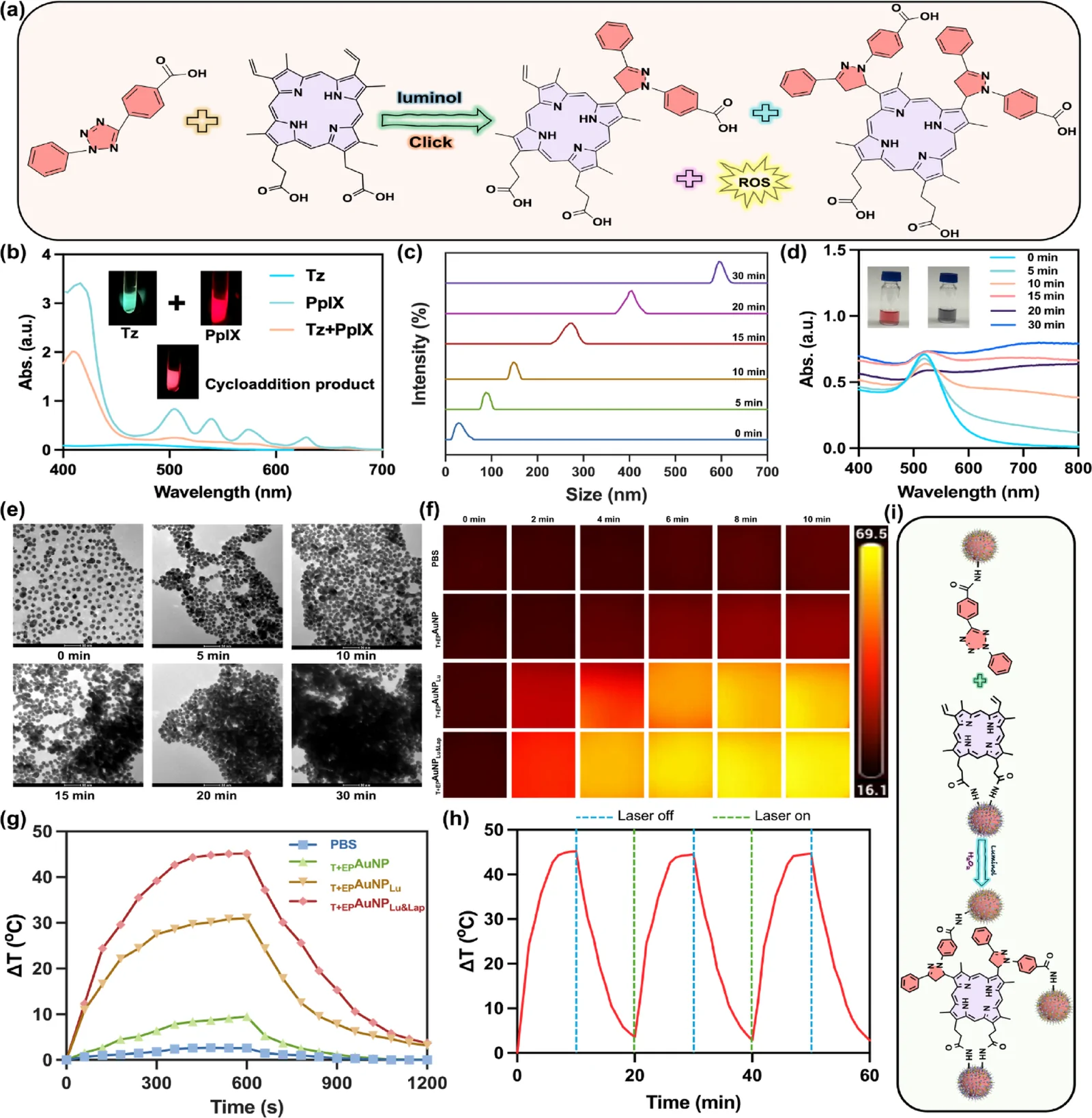
Corroboration of the aggregation ability of _T_AuNP_Lu&Lap_ and _EP_AuNP_Lu&Lap_. (**a**) Photoclick reaction between PpIX and Tz. (**b**) UV-Vis absorbance of Tz, PpIX and Tz+PpIX after exposed to 425 nm laser for 10 min. (**c**) DLS spectra, (**d**) UV-Vis absorbance, and d) TEM images of _T+EP_AuNP_Lu&Lap_ at different time incubated with 100 mM H_2_O_2_ solution and NQO1/NADH enzymes in Tris-HCl buffer for 30 min. (**e**) Photothermal images, and (**f**) record of temperature elevation of PBS, _T+EP_AuNP, _T+EP_AuNP_Lu_ and _T+EP_AuNP_Lu&Lap_ at different time point under 808 nm NIR irradiation. (**g**) Photothermal stability of _T+EP_AuNP_Lu&Lap_ under 808 nm irradiation for 3 cycles. (**h**) Illustration of the click-induced aggregation of _T_AuNP_Lu&Lap_ and _EP_AuNP_Lu&Lap_ in the presence of Lu and H_2_O_2_. (**i**) Scheme of PpIX/Tz click-induced AuNP aggregation

 To confirm the photo-crosslinking potential of _T_AuNP and _EP_AuNP, we first investigated whether the selected photoclick compounds, tetrazole (Tz) and PpIX, undergo light-induced cycloaddition, as illustrated in Fig. 3a. Specifically, Tz, PpIX, and their mixture were exposed to 425 nm laser irradiation (0.8 W cm^− 2^) for 15 min, followed by UV-Vis absorbance and mass spectrometry (MS) analysis. Altered absorption peaks (Fig. 3b) and new mass peaks at 799 and 1040 (Figure S14) confirmed formation of the cycloaddition product and efficient Tz-PpIX photocycloaddition after laser exposure. To further exclude nonspecific intracellular processes, including protein corona effects and lysosomal sequestration, the Tz-PpIX cycloaddition reaction was examined in cellular lysate by high-resolution mass spectrometry. The results revealed prominent peaks corresponding to the calculated molecular weights of the possible Tz-PpIX cycloaddition products, confirming that the click reaction proceeds effectively in a biologically complex environment and contributes to AuNP aggregation (Figure S15).

With this validated, we then examined whether Tz/PpIX cycloaddition facilitates AuNP aggregation. Experimentally, a mixture of _T_AuNP and _EP_AuNP with or without laser exposure (denoted as _T+EP_AuNP_L_ or _T+EP_AuNP, 200 µg mL^− 1^), was irradiated at 425 nm for 15 min, and hydrodynamic size changes were monitored using DLS. As depicted in Figure S16a, the hydrodynamic size of _T+EP_AuNP increased dramatically from 13 nm to ~740 nm over 15 min irradiation period. Additionally, Figure S16b illustrates the progressive emergence of an absorption peak at ~800 nm, along with a color change from wine red to bluish gray, indicating AuNP aggregation. TEM images further validated this transition, manifesting conspicuous AuNP clustering over time (Figure S16c). In sharp contrast, no significant changes were observed in _T+EP_AuNP (Figure S17) without laser exposure, confirming that the NIR irradiation effectively induced interparticle crosslinking via Tz/PpIX cycloaddition.

We then hypothesized that chemiluminescence of Lu could similarly trigger the aggregation of _T_AuNP_Lu&Lap_ and _EP_AuNP_Lu&Lap_, in addition to PDT efficacy. To test this, _T+EP_AuNP, _T+EP_AuNP_Lu_ and _T+EP_AuNP_Lu&Lap_ were incubated separately with H_2_O_2_ (100 mM) and NQO1/NADH enzymes in Tris-HCl buffer for 30 min. DLS spectra of _T+EP_AuNP_Lu&Lap_ dramatically increased from 13 nm to approximately 620 nm over 30 min (Fig. 3c). Additionally, Fig. 3d reveals the emergence of an ~800 nm absorption peak with the progression of incubation time, accompanied by a color change from wine red to bluish gray, indicative of aggregation of _T+EP_AuNP_Lu&Lap_. TEM images further exhibited pronounced aggregation in _T+EP_AuNP_Lu&Lap_, while negligible aggregation was observed in the _T+EP_AuNP and less in _T+EP_AuNP_Lu_ groups (Fig. 3e and S18). To identify the aggregation triggers, we incubated the nanoplatform under four conditions: pH 7.4/low GSH (10 µM), pH 6.5/low GSH (10 µM), pH 7.4/high GSH (10 mM), and pH 6.5/high GSH (10 mM), with H_2_O_2_ and NQO1/NADH enzymes for 30 min (Figure S19). The results indicate higher pH environment and lower GSH favor the aggregation behavior.

Based on the above confirmation, PTT efficiency was subsequently evaluated. After aggregation, _T+EP_AuNP, _T+EP_AuNP_Lu_ and _T+EP_AuNP_Lu&Lap_ were subjected to 808 nm NIR irradiation (1.25 W cm^− 2^) for 10 min, and the temperature rise was recorded. As shown in Fig. 3f g, the _T+EP_AuNP_Lu&Lap_ solution reached a temperature of approximately 67.2 °C, with a photothermal conversion efficiency (*η*) of 68.15%, significantly outperforming _T+EP_AuNP_Lu_ (53.3 °C, *η* = 39.92%) and _T+EP_AuNP (31.5 °C, *η* = 25.14%). Moreover, the temperature of the solution could be finely regulated by adjusting both the laser power and the concentration of AuNPs, as demonstrated in Figure S20. This adaptability in temperature regulation is crucial for optimizing PTT treatments while minimizing potential side effects. Importantly, the aggregates displayed superior photothermal stability, resisting disassembly at elevated temperatures (Fig. 3 h). Figure 3i illustrates the aggregation mechanism induced by Lu chemiluminescence via Tz/PpIX click cycloaddition. In summary, these findings highlight the remarkable improvement in PTT efficacy achieved through the Lu-triggered aggregation of AuNPs. By enhancing the photothermal conversion efficiency, providing superior temperature regulation, and ensuring structural stability, the _T+EP_AuNP_Lu&Lap_ aggregates offer a robust approach for more effective and controlled PTT treatments, minimizing undesired side effects.

### *In vitro* cytotoxicity and Type I PDT performance

**Fig. 4:**
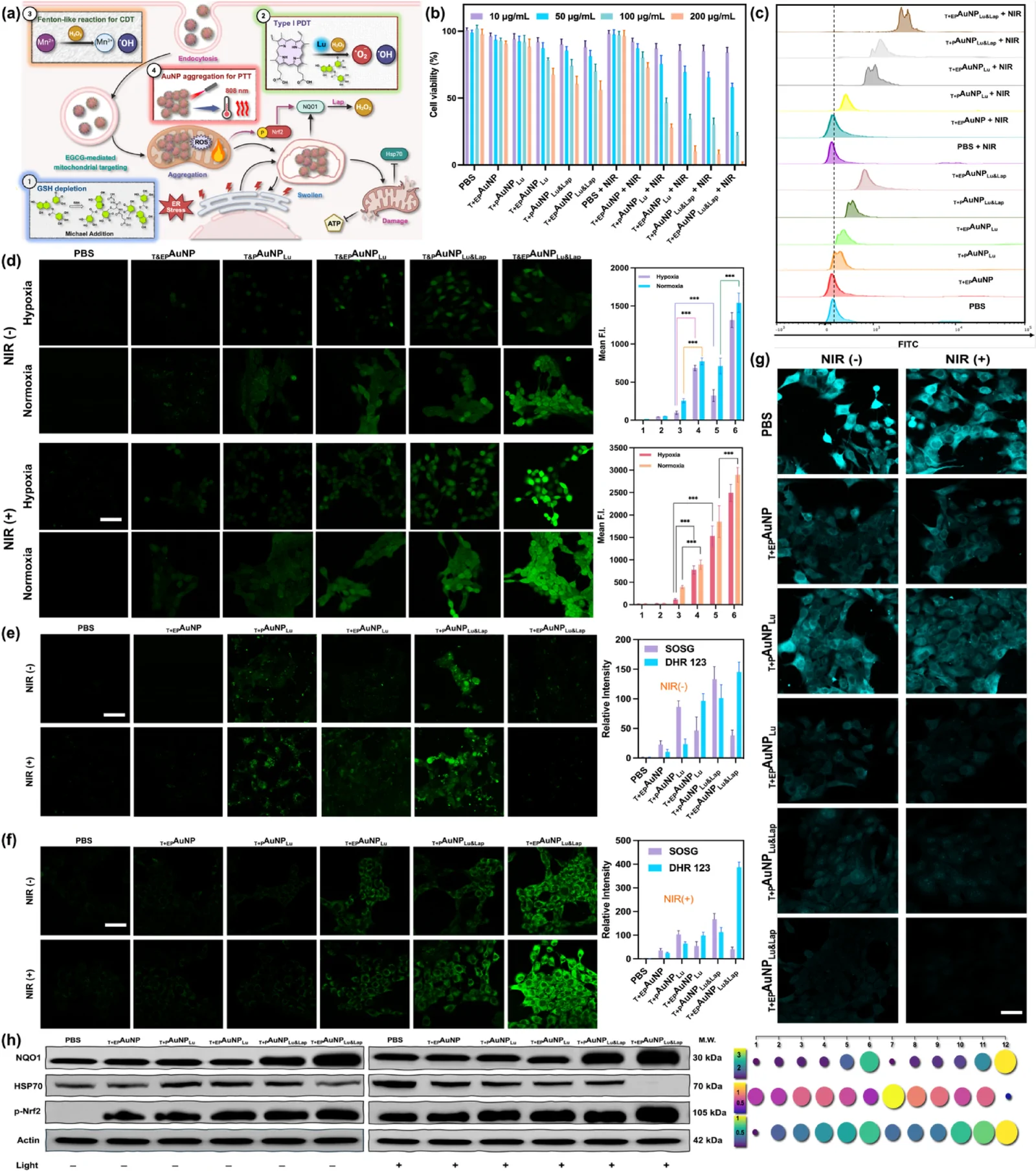
In vitro validation of therapeutic effects and Type I PDT transformation. (**a**) Scheme of therapeutic modalities. (**b**) Cell viability of 4T1 cells after incubation with _T+EP_AuNP_Lu&Lap_ and its counterparts with or without 808 nm NIR irradiation. (**c**) DCFH-DA staining flow cytometry for measuring ROS production. (**d**) DCFH-DA staining CLSM images for intracellular ROS generation, and quantitative analysis of mean fluorescence intensity. (**e**) SOSG staining CLSM images for intracellular ^1^O_2_ production. (**f**) DHR123 staining CLSM images for intracellular O_2_^•^⁻ production. Comparison of ^1^O_2_ and O_2_^•^⁻ production of _T+EP_AuNP_Lu&Lap_ and its counterparts with or without 808 nm NIR irradiation. (**g**) ThiolTracker Violet staining CLSM images for evaluation of intracellular GSH depletion. (**h**) WB analysis of mitochondria-related proteins and quantitative visualization. Quantitative data are presented as mean ± SD (*n* = 3). Scale bar: 50 $\:\mu\:$m

The _T+EP_AuNP_Lu&Lap_ nanoplatform combines multiple therapeutic modalities to synergistically boost ROS generation, amplified by EQ-mediated GSH depletion. Due to intrinsic mitochondria-targeting capability of EGCG, _T+EP_AuNP_Lu&Lap_ specifically accumulates inside mitochondria, directly compromising their integrity and thus leading to ATP depletion and the upregulation of Nrf2, the principal transcriptional regulator of NQO1, which induces NQO1 upregulation. HSP70 expression was suppressed due to the lack of ATP. Meanwhile, H_2_O_2_ production and Lu chemiluminescence were greatly amplified by Lap and upregulated NQO1, favoring Mn^2^⁺-mediated CDT and Lu-involved PDT to achieve superior therapeutic outcomes (Fig. 4a).

To evaluate the therapeutic efficacy, cytotoxicity was first assessed via MTT assay. As shown in Figure S21, _T+EP_AuNP_Lu&Lap_ and its counterparts showed minimal cytotoxicity toward L929 fibroblasts, with cell viability above 80% at 200 µg mL^− 1^. This indicates low toxicity and good biosafety in non-cancerous cells, likely due to TME-responsive activation. In contrast, _T+EP_AuNP_Lu&Lap_ strongly killed 4T1 breast cancer cells, reducing viability to 56.2% without NIR and 1.1% after 808 nm irradiation (Fig. 4b), consistent with live/dead staining and flow cytometry (Figures S22 and S23). Additionally, to further clarify the relationship between ROS generation and cytotoxicity, 4T1 cells were pre-treated with the ROS scavenger TEMPO. TEMPO partially rescued cell viability from 1.1% to 28.6% in the _T+EP_AuNP_Lu&Lap_ + NIR group and from 56.2% to 77.4% in the _T+EP_AuNP_Lu&Lap_ group without NIR (Figure S24). These results confirm that ROS generation actively contributes to cytotoxicity. Importantly, the persistent suppression of cell viability after ROS scavenging indicates that AuNP-mediated PTT remains a major driver of cell death, as the irreversible thermal ablation induced by aggregated AuNPs cannot be fully rescued by antioxidants. Noticeably, Combination Index (*CI*) value via Chou-Talalay method for the synthesized nanoplatform is 0.2, substantially lower than 1, robustly confirming a highly synergistic therapeutic interaction among these modalities.

To verify Type I-dominated PpIX conversion at the cellular level, we measured ROS by flow cytometry (Fig. 4c). _T+EP_AuNP_Lu&Lap_ produced markedly higher ROS than the corresponding counterparts, with or without NIR. More specifically, CLSM images shown in Fig. 4d demonstrated 4T1 breast tumor cells treated by _T+EP_AuNP_Lu&Lap_ exhibited a significant enhancement of ROS generation capacity, 2.17-fold and 4.07-fold (1.56-fold and 1.62-fold with NIR) elevated, compared with that treated by _T+P_AuNP_Lu&Lap_ under both normoxia and hypoxia, respectively. This enhancement is attributed to the hypoxia-tolerant Type I PDT mechanism and •OH generation from Mn^2^⁺-mediated CDT, proved in the _T+EP_AuNP group that produced discernible ROS under all conditions in tumor cells. After NIR irradiation, a similar trend was observed, with elevated ROS generation in _T+P_AuNP_Lu&Lap_ under hypoxia, possibly due to PTT-induced mitochondria stress. More particularly, intracellular ^1^O_2_ and O_2_^•^⁻ production were detected using singlet oxygen sensor green (SOSG) and dihydrorhodamine 123 (DHR 123), respectively. As shown in Fig. 4e and f, an obviously boosted O_2_^•^⁻ production (1.44-fold and 3.41-fold higher with NIR) and a suppressed ^1^O_2_ yield (3.5-fold and 4.18-fold lower with NIR) were observed in _T+EP_AuNP_Lu&Lap_, strongly evincing a Type I-dominated PDT mechanism in the presence of EGCG. In contrast, _T+P_AuNP_Lu&Lap_ manifested a Type II-dominated mechanism, evidenced by enhanced ^1^O_2_ production and negligible O_2_^•^⁻ production.

EGCG is easily oxidized to EQ by abundant intracellular ROS. EQ undergoes efficient Michael addition with -SH and -NH₂ groups of GSH, leading to GSH depletion and further amplifying oxidative stress in tumor cells. ThiolTracker Violet dye was used to visualize changes in GSH concentration. As shown in Fig. 4g and S25, _T+EP_AuNP_Lu&Lap_ exhibits exceptional GSH depletion ability. Western blot (WB) analysis was conducted to measure the expression levels of key proteins, including NQO1, HSP70, and p-Nrf2 as illustrated in Fig. 4h. The results demonstrated the most pronounced upregulation of NQO1 in _T+EP_AuNP_Lu&Lap_ + NIR, which enhanced its catalysis over Lap to produce H_2_O_2_. The expression of HSP70, which requires ATP involvement, was significantly suppressed due to mitochondrial damage. This suppression was most evident in _T+EP_AuNP_Lu&Lap_ + NIR group. Moreover, _T+EP_AuNP_Lu&Lap_ + NIR most effectively upregulated p-Nrf2, indicating severe mitochondrial dysfunction.

### *In vitro* cGAS-STING activation and ER stress

**Fig. 5:**
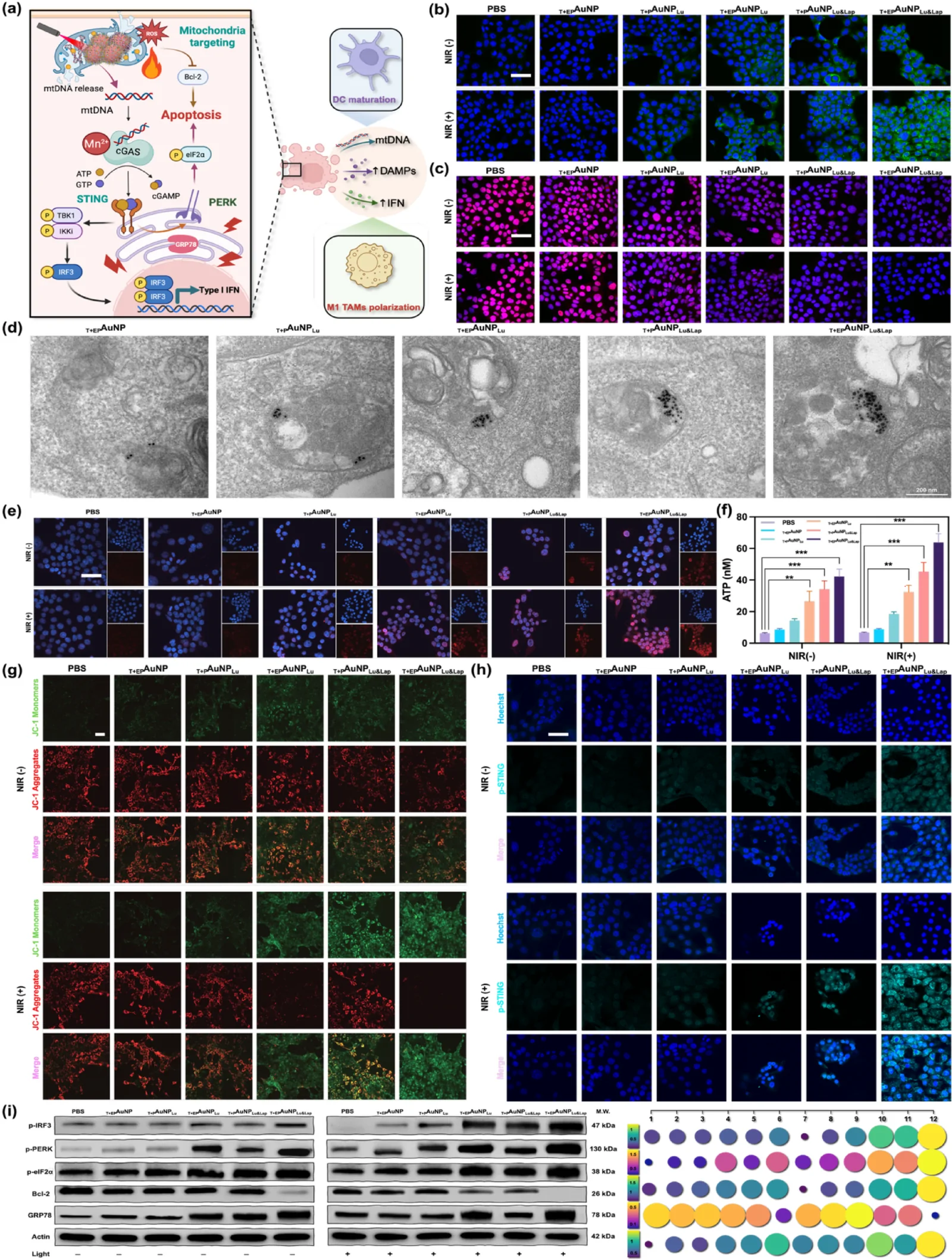
In vitro verification of ER stress and cGAS-STING pathway. (**a**) Schematic demonstration of induction of ER stress and activation of cGAS-STING pathway. CLSM images of (**b**) CRT exposure, and (**c**) HMGB1 migration after different treatment. (**d**) Representative bio-TEM images to visualize intracellular _T_AuNP_Lu&Lap_ and _EP_AuNP_Lu&Lap_ aggregation and their counterparts. (**e**) Verification of intracellular DNA release. γ-H2AX staining CLSM images for double-stranded breaks in DNA (**f**) Detection of released ATP using ATP assay kit. (**g**) JC-1 staining images for mitochondria membrane integrity assessment. (**h**) p-STING staining CLSM images for activation of cGAS-STING pathway. (**i**) Western blot analysis of ER stress- and cGAS-STING-related proteins, with quantitative visualization. Quantitative data are presented as mean ± SD (*n* = 3). Scale bar: 20 $\:\mu\:$m

A clear mechanism between ER stress and its enhancement to cGAS-STING activation is illustrated in Fig. 5a. The key chaperone GRP78 dissociates from ER under stress, activating PERK and inducing its phosphorylation (p-PERK). This triggers the phosphorylation of eIF2α (p-eIF2α), leading to tumor cell apoptosis [48, 49]. ROS- and heat-induced mitochondrial dysfunction suppresses anti-apoptotic Bcl-2, elicits ICD and DAMP release, and promotes mtDNA leakage into the cytosol. In the presence of Mn^2+^, cytosolic mtDNA activates cGAS and generates 2’3’-cGAMP. The 2’3’-cGAMP binds to ER-resident STING, whose C-terminal domain directly interacts with the kinase domain of PERK on ER membrane for its activation via a non-canonical pathway. Additionally, STING recruits TBK1 and IKKi to phosphorylate and activate IRF3 (p-IRF3), driving type I IFN production. The released mtDNA, after internalization by TAMs, facilitates M1-type polarization via cGAS-STING pathway activation [50]. 

Effective hypoxia relief is conducive to an immunostimulatory environment, which was first evidenced by O_2_-sensitive Ru(dpp)_3_Cl_2_ staining outcome (Figure S26). To evaluate ICD in tumor cells induced by _T+P_AuNP_Lu&Lap_, DAMPs such as CRT, HMGB1 and ATP were detected. Abundant CRT was detected on the cell membranes in _T+EP_AuNP_Lu&Lap_ (+ NIR) groups (Fig. 5b). Regarding HMGB1, intense red fluorescence overlapping with nuclei diminished in experimental groups and was almost absent in _T+EP_AuNP_Lu&Lap_ (+ NIR) groups (Fig. 5e), indicating effective release of HMGB1 from neclei. Extracellular ATP content was assessed using ATP assay kit, and results showed that _T+EP_AuNP_Lu&Lap_ (+ NIR) induced enormous extracellular ATP release, 6.8-times and 9.5-times with NIR more than PBS groups (Fig. 5f). These results evidently confirmed that _T+EP_AuNP_Lu&Lap_ triggered large-scale ICD in tumor cells after NIR irradiation.

Given EGCG’s intrinsic mitochondria targeting ability, MitoTracker staining fluorescence was used to visualize the targeting ability of _T_AuNP_Lu&Lap_ (FITC dyed) and _EP_AuNP_Lu&Lap_ (Cy5.5 dyed) and its counterparts. The EGCG-containing _T+EP_AuNP, _T+EP_AuNP_Lu_, and _T+EP_AuNP_Lu&Lap_ groups exhibited strong fluorescence overlap with mitochondria, with _T+EP_AuNP_Lu&Lap_ showing the highest degree of co-localization. In contrast, the EGCG-free T+PAuNPLu&Lap group showed negligible mitochondrial fluorescence overlap, confirming the critical role of EGCG in mitochondrial targeting and supporting the enhanced aggregation capability of _T+P_AuNP_Lu&Lap_ in mitochondria-rich regions (Figure S27). To assess ER localization due to the close connection between mitochondria and ER, ER Tracker was used with _T_AuNP_Lu&Lap_ (Rhodamine dyed) and _EP_AuNP_Lu&Lap_ (Cy5.5 dyed) and their counterparts. Similarly, _T+EP_AuNP_Lu&Lap_ showed the most prominent overlap and intense fluorescence, confirming its ER localization ability to augment ER stress (Figure S28).

To further visualize aggregation, bio-TEM tests were conducted. As shown in Figure 5d, no aggregation occurred intracellularly in 4T1 cells incubated with _T+EP_AuNP. Limited aggregation was observed in _T+P_AuNP_Lu_, while stronger aggregation occurred in _T+EP_AuNP_Lu_ and _T+P_AuNP_Lu&Lap_. In sharp contrast, significant nanoaggregate formation was visible in _T+EP_AuNP_Lu&Lap_ treated cells, confirming intracellular Tz/PpIX click reactions enhanced by Lap in the presence of Lu. To prove that aggregation was specifically triggered by the TME rather than a mere concentration-induced artifact, we performed parallel bio-TEM imaging on L929 cell lines incubated with the exact same concentration of the same groups (Figure S29). The rationale is that normal cells lack the hallmark characteristics of the TME, specifically, the highly elevated endogenous H_2_O_2_ and overexpressed NQO1 enzymes, that are absolutely required to drive the luminol chemiluminescence and initiate the subsequent bio-orthogonal Tz/PpIX click cycloaddition. The results indicated no obvious aggregation and nearly mono-dispersity was observed for all groups, ruling out the possibility that the clustering was simply due to high local particle abundance and confirming that the aggregation was a TME-responsive, click-chemistry-driven event.

The effect of _T+EP_AuNP_Lu&Lap_ on cellular DNA was explored via γ-H2AX staining, a marker of DNA damage. CLSM images revealed that the fluorescence of γ-H2AX appeared the most intense within cells treated with _T+EP_AuNP_Lu&Lap_ + NIR, indicative of severe mitochondria and DNA damage (Fig. 5e). Quantitatively, fluorescence of γ-H2AX in cells treated with _T+EP_AuNP_Lu&Lap_ + NIR was 1.6-, 2.2-, and 1.45-fold higher than that in _T+EP_AuNP_Lu&Lap_, _T+P_AuNP_Lu&Lap_ and _T+P_AuNP_Lu&Lap_ + NIR groups, respectively (Fig. 30). The results firmly demonstrate the potency of introduction of mitochondria-specific PTT. Additionally, bio-TEM images demonstrated severe ER stress and mitochondria swelling in the _T+EP_AuNP_Lu&Lap_ + NIR group (Figure S31).

We anticipated the observed therapeutic amplification arises from synergistic coupling rather than the simple additive effect of independent dominant modules. The modules compensate for each other’s thermodynamic and kinetic limitations: PTT accelerates the rate-limiting CDT, while the combined oxidative stress from CDT and Type I PDT comprehensively disrupts tumor cell defenses. This massive, coordinated local damage maximizes the release of DAMPs to robustly prime the subsequent systemic immunotherapy cascade. A value of *CI* as low as 0.2 mathematically substantiates that the mutually reinforcing cycle maximizes the tumoricidal response far beyond the any of its individual components.

Mitochondrial membrane integrity was assessed via JC-1 staining. The combination of PTT induced more severe mitochondria damage as compared to those without NIR irradiation. The _T+EP_AuNP_Lu&Lap_ + NIR group exhibited the most intense JC-1 monomer/aggregate ratio, indicating the most severe mitochondrial dysfunction (Fig. 5g and S32). Reasonably, abundant mtDNA thus released could dramatically activate cGAS-STING pathway, validated via p-STING fluorescence. As depicted in Fig. 5h and S33, p-STING fluorescence exhibited similar results, in line with those of JC-1 and γ-H2AX staining. _T+EP_AuNP_Lu&Lap_ + NIR induced strongest STING activation, due to the sensitization of cGAS toward the released mtDNA, facilitating ER stress in addition to the ROS and heat production. WB analysis was conducted to assess relevant protein expression. As illustrated in Fig. 5i, expression levels of GRP78, p-PERK and p-eIF2α were most elevated in _T+EP_AuNP_Lu&Lap_ + NIR group, showing severe ER stress induced. Furthermore, p-IRF3 was also notably upregulated, indicating strong STING activation and thus type I IFN release. Anti-apoptotic Bcl-2 expression was suppressed and downregulated, denoting mitochondrial dysfunction.

### Click addition induced aggregation of AuNP in vivo

**Fig. 6:**
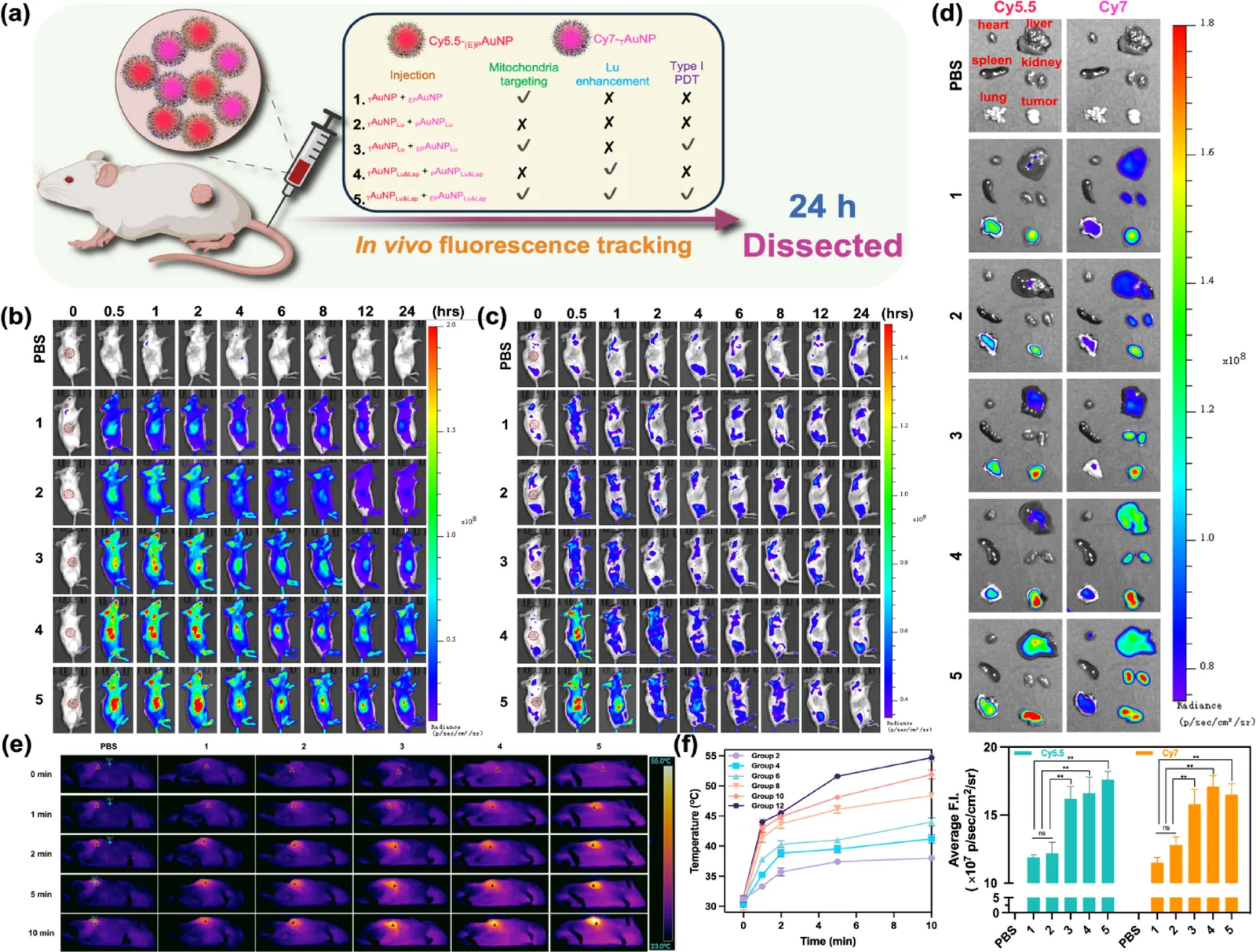
In vivo confirmation of aggregation of _T_AuNP_Lu&Lap_ and _EP_AuNP_Lu&Lap_. (**a**) Schematic illustration of probing aggregation ability of _T_AuNP_Lu&Lap_ and _EP_AuNP_Lu&Lap_ and their counterparts. (**b**) In vivo fluorescent images of mice injected with Cy5 labelled _T_AuNP_Lu&Lap_ or its counterparts. (**c**) In vivo fluorescent images of mice injected with Cy7 labelled _EP_AuNP_Lu&Lap_ or its counterparts. (**d**) Fluorescent images of dissected mice organs (top), and quantitative analysis of fluorescent intensity on dissected tumor (bottom). (**e**) Thermal images and (**f**) temperature elevation record of mice injected with different formula of AuNP receiving irradiation of 808 nm NIR laser for 10 min. Quantitative data are presented as mean ± SD (*n* = 3).

 Subsequently, in vivo studies were conducted to examine the PTT effect of _T+EP_AuNP_Lu&Lap_ realized by the click-induced aggregation of AuNP triggered by Lu luminescence. Hemolysis assay was first performed to ensure biosafety. The results confirmed negligible red blood cells lysis up to 800 µg mL^− 1^ (Figure S34), underscoring its reliable biocompatibility. The retention of AuNP at tumor site is anticipated to be markedly prolonged due to the formation of nanoaggregates. To testify this, five experimental groups were established, denoted as (1) _T_AuNP + _EP_AuNP; (2) _T_AuNP_Lu_ + _P_AuNP_Lu_; (3) _T_AuNP_Lu_ + _EP_AuNP_Lu_; (4) _T_AuNP_Lu&Lap_ + _P_AuNP_Lu&Lap_; (5) _T_AuNP_Lu&Lap_ + _EP_AuNP_Lu&Lap_, where _(E)P_AuNP was labelled with Cy5.5, and _T_AuNP with Cy7 (Fig. 6a). Tumor-bearing BALB/c mice were intravenously injected with the components of each group. In vivo fluorescent images were taken at different time points after injection. Groups 4 and 5 showed stronger Cy5.5 and Cy7 tumor signals than the non-Lap counterparts throughout the experiment (Fig. 6b and c). This indicates Lap-enhanced H_2_O_2_ enrichment and AuNP aggregation, as supported by prolonged fluorescence retention and ICP-MS Au quantification (Figure S35). To better contextualize tumor selectivity, we calculated tumor-to-organ accumulation ratios, including tumor/liver and tumor/spleen ratios. The quantitative accumulation ratios clearly demonstrated that the tumor-to-organ ratios, including tumor/liver and tumor/spleen ratios, significantly escalated for _T+P_AuNP_Lu&Lap_ and _T+EP_AuNP_Lu&Lap_ group due to the specific prevention of tumor efflux, robustly proving their higher degree of tumor selectivity (Figure S36). Fluorescent intensity 24 h post-injection in the dissected tumors was measured. Dissected tumors showed stronger Cy5.5 and Cy7 signals than major organs in all groups, whereas kidney, lung, and liver showed weaker signals and heart and spleen showed no visible signals. These results suggest favorable biosafety and tumor retention (Fig. 6 d). The pharmacokinetic parameter (*t*_1/2_) of _T_AuNP, _T_AuNP_Lu_, _T_AuNP_Lu&Lap_, _EP_AuNP, _P_AuNP_Lu_, _EP_AuNP_Lu_, _P_AuNP_Lu&Lap_ and _EP_AuNP_Lu&Lap_ was calculated as 7.29 h, 6.80 h, 6.22 h, 7.9 h, 5.46 h, 7.62 h, 5.61 h and 6.32 h (Figure S37), respectively. Ex vivo time-dependent biodistribution results also demonstrated that all AuNP mainly accumulated in tumors, with some distribution in liver and spleen (Figure S89 and S39). Notably, we observed a steady decline of both Au and Mn in the renal and hepatobiliary systems over time, indicating effective clearance and mitigating concerns regarding permanent heavy/transition metal accumulation.

Encouraged by these results, the in vivo photothermal conversion effect of AuNP aggregates was examined by profiling temperature elevation at tumor sites. In group 5 that received regimen of treatment of _T+EP_AuNP_Lu&Lap_, the local tumor temperature increased drastically to 54.7 °C after 10 min of 808 nm laser irradiation (1.25 W cm^− 2^), while the local temperature of tumor in group 4 receiving _T+P_AuNP_Lu&Lap_ was elevated to 51.6 °C. In sharp contrast, tumors in mice without AuNP injection remained merely below 45 °C under the same laser conditions (Fig. 6e and f).

These findings firmly testified that Lap-mediated H_2_O_2_ enrichment in tumors facilitates Tz/PpIX-induced AuNP aggregation triggered by Lu luminescence, resulting in a significantly enhanced PTT effect. Moreover, Type I ROS generation could further augment NQO1 expression, thereby promoting its reaction with Lap to induce higher tumor H_2_O_2_ concentration. This was substantiated by in vivo fluorescence imaging and the prolonged retention of AuNP aggregates in tumors.

### *In vivo* validation of therapeutic efficacy

**Fig. 7:**
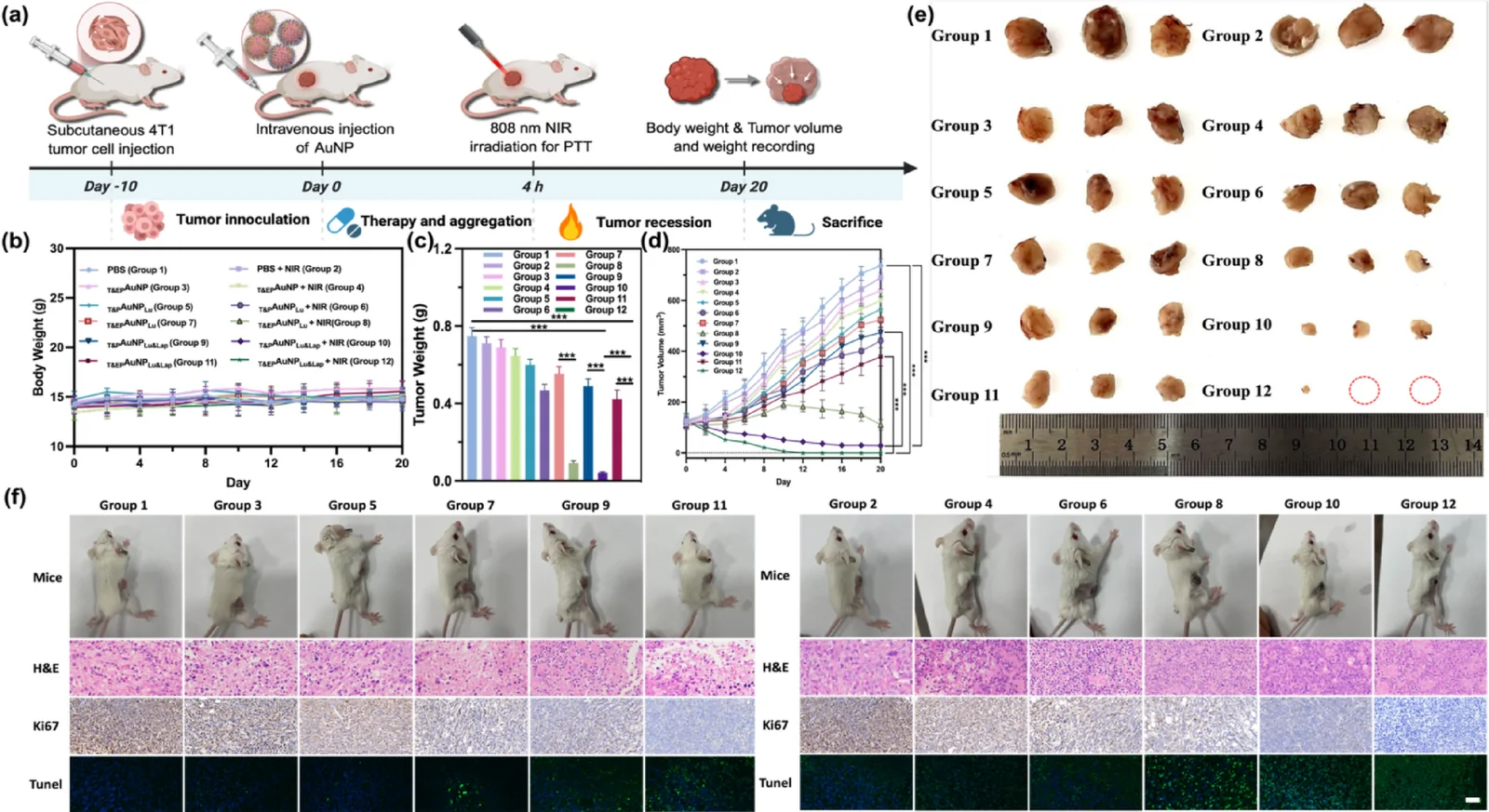
Evaluation of in vivo therapeutic efficacy on 4T1 tumor-bearing BALB/c mice models. (**a**) Therapeutic schedule and timeline for 4T1 tumor-bearing mice. (**b**) Body weight, (**c**) tumor weight, and (**d**) tumor volume records of mice from day 1 to 20. (**e**) Images of the dissected tumors at the end of treatment. (**f**) H&E staining, Ki67 IHC, and TUNEL staining images of tumor sections from each group of mice. Quantitative data are presented as mean ± SD (*n* = 3). Scale bar: 50$\:\:\mu\:$m

 Preceding investigations exhibited remarkable therapeutic efficacy of _T+EP_AuNP_Lu&Lap_ with multimodal synergy. Its feasibility was further evaluated in vivo using BALB/c mice bearing 4T1 tumors. When tumor volumes reached approximately 120 mm^3^, the mice were divided into 12 groups with distinct treatment regimens: Group 1: PBS; Group 2: PBS + NIR; Group 3: _T+EP_AuNP; Group 4: _T+EP_AuNP + NIR; Group 5: _T+P_AuNP_Lu_; Group 6: _T+P_AuNP_Lu_ + NIR; Group 7: _T+EP_AuNP_Lu_; Group 8: _T+EP_AuNP_Lu_ + NIR; Group 9: _T+P_AuNP_Lu&Lap_; Group 10: _T+P_AuNP_Lu&Lap_ + NIR; Group 11: _T+EP_AuNP_Lu&Lap_; Group 12: _T+EP_AuNP_Lu&Lap_ + NIR. NIR irradiation was applied 4 h post-injection to allow sufficient aggregation of AuNPs. Mice body weight, tumor volume, and excised tumor mass were tracked over 20 days (Fig. 7a).

As elucidated in Fig. 7b-e, mice maintained stable body weight throughout the treatment period, reflecting negligible systemic toxicity. Tumor progression remained largely unrestrained in Groups 1, 2, 3, and 4. Moderate tumor inhibition was observed in Groups 5 and 6, while Groups 7 and 8 exhibited stronger inhibition, indicating EGCG significantly enhanced therapeutic efficacy. Notably, Groups 11 and 12 showed the most pronounced tumor suppression, outperforming Groups 9 and 10. This was evidenced by near-complete tumor eradication and significantly reduced tumor volume and mass, suggesting that Lap augmented H_2_O_2_ production and stimulated interplay among therapeutic modalities. These results underscore robust therapeutic efficacy and synergistic effects of _T+EP_AuNP_Lu&Lap_. Histopathological assessment following treatment, as evidenced by hematoxylin and eosin (H&E), Ki67 and (TdT-mediated dUTP nick-end labeling) TUNEL staining, revealed extensive nuclear pyknosis, necrosis, and apoptosis in tumors from Group 12, in stark contrast to the densely packed malignant cells observed in its comparative groups (Fig. 7f). Importantly, H&E staining images of major organs disclosed no detectable pathological abnormalities or metastasis (Figure S40). Moreover, comprehensive hematological and biochemical analyses confirmed no signs of systemic toxicity of either _T+EP_AuNP_Lu&Lap_ or its counterparts (Figure S41).

### Remodeling tumor microenvironment to potentiate antitumor immunity

**Fig. 8:**
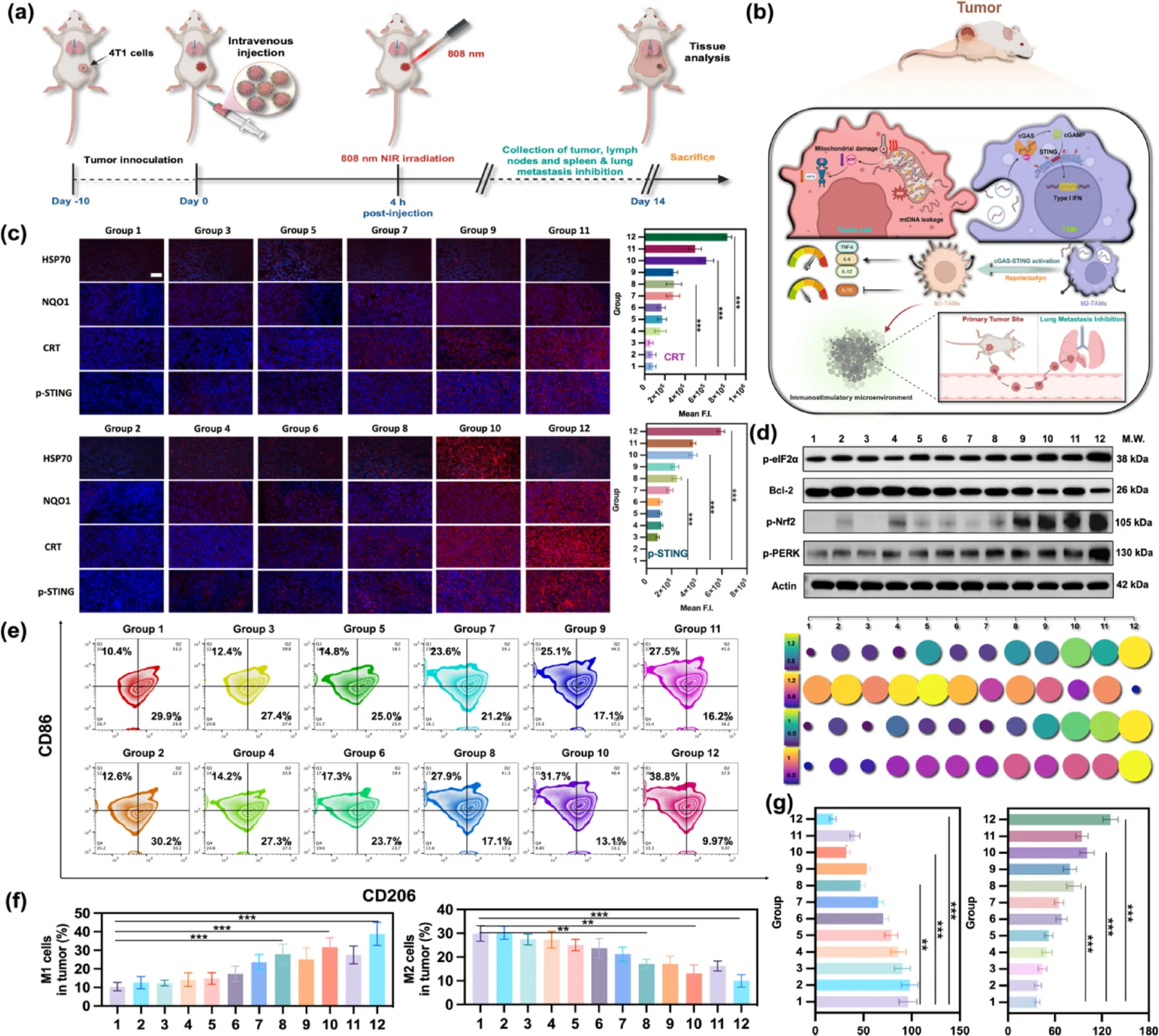
Assessment of TAMs polarization and DC maturation in vivo. (**a**) Schematic representation of in vivo experiment for analysis of TAMs polarization and secretion of associated cytokines. (**b**) Illustration of mitochondria damage for cGAS-STING induced M1 macrophage polarization and corresponding cytokines secretion for lung metastasis inhibition. (**c**) Immunofluorescence of HSP70, NQO1, CRT and p-STING expression. (**d**) Western blot analysis of ER stress related proteins expression and quantitative visualization. (**e**) Flow cytometry plots and the corresponding quantification of (**f**) M1 TAMs (CD86^+^CD206^−^) and M2 TAMs (CD86^−^CD206^+^) in tumor. Secretion levels of (**g**) IL-10 (left) and IL-12 (right) in the supernatant after different treatments. Quantitative data are presented as mean ± SD (*n* = 3). Scale bar: 50$\:\:\mu\:$m

 To validate antitumor immune responses, tumor-bearing BALB/c mice were intravenously injected with _T+EP_AuNP_Lu&Lap_ and its counterparts (same as the 12 groups set previously). After 14 days of treatment, organs and tumors were collected for analysis (Fig. 8a). ROS and heat induced mitochondria damage resulted in the cytosolic release of mtDNA. TAMs internalized extracellular mtDNA from tumor tissues, which activated cGAS-STING signaling and promoted M1-like polarization. This phenotype was associated with increased IL-6, IL-12, and type I IFN (e.g., IFN-α) and reduced anti-inflammatory IL-10. Consequently, this dynamic interplay between mtDNA-mediated innate immune activation and macrophage polarization reprogrammed TME (Fig. 8b).

Immunofluorescence results demonstrated the modulation of HSP70, NQO1, CRT, and cGAS-STING activation in tumor tissues (Fig. 8c and S42). Immunofluorescence of HSP70 diminished significantly in _T+EP_AuNP_Lu&Lap_ group, owing to severe mitochondrial damage thus ATP depletion. Nonetheless, _T+EP_AuNP_Lu&Lap_ + NIR showed limited HSP70 expression compared with _T+P_AuNP_Lu&Lap_ + NIR group. Conversely, NQO1 fluorescence was markedly strengthened in response to mitochondrial dysfunction, activating Nrf2 expression. Phosphorylated Nrf2 translocates into nuclei and induces transcription of antioxidant NQO1 gene. The observed NQO1 protein upregulation therefore reflects a compensatory cellular adaptation to oxidative stress. CRT expression was most elevated in _T+EP_AuNP_Lu&Lap_ + NIR group, indicating effective induction of ICD, in addition to a prominent activation of cGAS-STING pathway, evidenced by intensified p-STING fluorescence. WB analysis further confirmed these findings, with p-PERK and p-eIF2α levels showing the most significant increases in _T+EP_AuNP_Lu&Lap_ + NIR group (Fig. 8 d), indicating effective PERK activation in response to ER stress. Enhanced p-Nrf2 expression reflected heightened antioxidant signaling. In contrast, most prominently suppressed Bcl-2 expression in _T+EP_AuNP_Lu&Lap_ + NIR, signifying a loss of mitochondrial integrity and a shift toward apoptotic susceptibility.

TAMs polarization from the M2 to M1 phenotype is a critical strategy for alleviating tumor-mediated immunosuppression [51]. As demonstrated in Fig. 8e and f, the proportion of M1- (CD86^+^CD206^−^) and M2-type (CD86^−^CD206^+^) TAMs in mouse tumors were analyzed. Compared to Group 1, M2-type TAMs were clearly reduced in Group 12 (29.9%–9.97%), whereas the percentage of M1-type TAMs increased from 10.4% to 38.8%. We further detected the cytokine level in the supernatant of tumor homogenates. As shown in Fig. 8g, the inflammation-suppressing IL-10, secreted by M2-type TAMs, was reduced by 5.27-fold in Group 12 when compared to Group 1, and the proinflammatory cytokine IL-12 secreted by M1-type TAMs increased by 3.50-fold, suggesting that the _T+EP_AuNP_Lu&Lap_ was able to polarize tumor growth-promoting M2-type TAMs into tumor-suppressing M1-type TAMs, magnifying antitumor immunity and therapeutic efficacy. Furthermore, the maturation rate of DCs in tumor-infiltrating lymph nodes was also significantly higher in Group 12, as shown in Figure S43.

### Activated immune response and lung metastasis inhibition

**Fig. 9:**
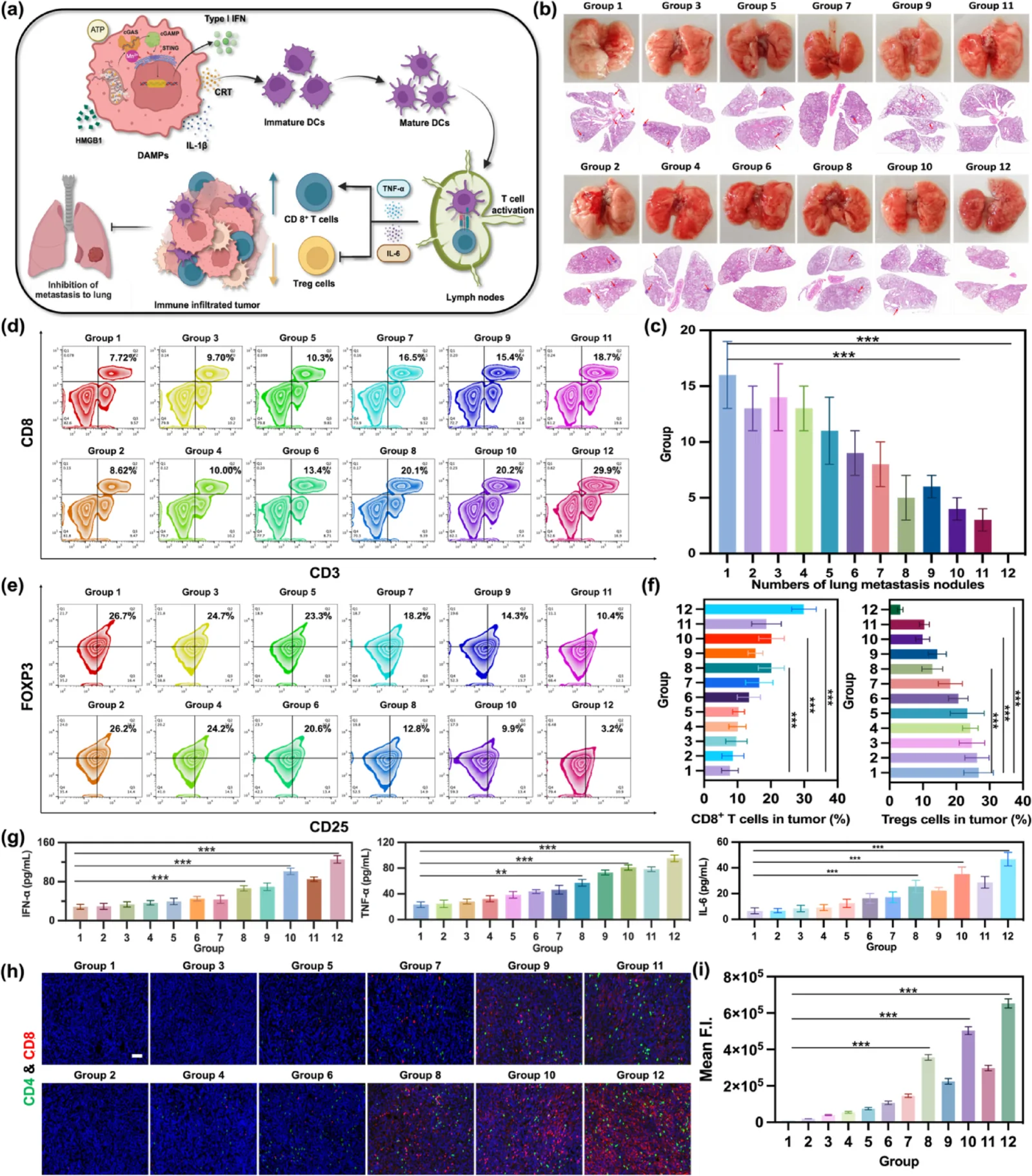
Appraisal of immune stimulation effect in vivo. (**a**) Schematic presentation DC maturation, enhanced CD8^+^ T cell activation, and lung metastasis inhibition. (**b**) Representative dissected lung tissues and H&E staining images, with (**c**) quantification of numbers of lung metastasis nodules. The populations of (**d**) CD8^+^ T cells and (**e**) Tregs and their (**f**) quantitative analysis in tumor tissues after different treatments. (**g**) Evaluation of secretion levels of immune-related cytokines IFN-α, TNF-α and IL-6 in different groups after treatment. (**h**) Immunofluorescence of CD4^+^ T cells and CD8^+^ T cells in tumor tissues after different treatments and (**i**) Quantitative analysis. Quantitative data are presented as mean ± SD (*n* = 3). Scale bar: 50$\:\:\mu\:$m

 In addition to potent DC maturation inducer IFN-α elicited by cGAS-STING activation, DAMPs that are released from stressed or dying cells are recognized by DCs, triggering their activation and maturation. Mature DCs serve as potent antigen-presenting cells that efficiently prime and activate CD8⁺ T cells. IL-6, produced by activated DCs, enhances the survival, proliferation, and effector differentiation of CD8⁺ T cells. Tumor necrosis factor-α (TNF-α) secreted by DCs and macrophages acts synergistically to potentiate T cell activation and facilitate the recruitment and retention of CD8⁺ T cells within lymph nodes. These activated CD8⁺ T lymphocytes are pivotal not only for the battle of tumors, but also for the targeted recognition and destruction of metastatic tumor cells within the pulmonary microenvironment. Concurrently, the inhibition of Tregs alleviates immunosuppressive barriers, thereby augmenting CD8^+^ T cell activity and promoting robust antitumor immunity. The synergistic interplay between DC maturation, CD8⁺ T cell activation, and Treg suppression is thus critical to tumor eradication and curtailing pulmonary metastasis (Fig. 9a).

To demonstrate cancer inhibition ability of _T+EP_AuNP_Lu&Lap_, lung metastasis mice models were established by intravenous injection of 4T1 cells. Figure 9b and c show almost no pulmonary tumor nodules in Group 12, whereas other groups had visible nodules. This suggests that the combined treatment and activated immunotherapy are essential for lung-metastasis eradication. Given that DC maturation is pivotal for T-cell priming, we employed flow cytometry to assess splenic CD8^+^ T cell activation after treatment. the upregulation of CD8^+^ T cells represents a direct manifestation of systemically activated immune responses. CD8^+^ T cells populations were markedly increased by 22.18% and 33.1% in tumor tissues and spleen, respectively (Fig. 9 d and S44), reflecting enhanced adaptive immune responses.

In parallel, the accumulation of Tregs, critical mediators of immunosuppression and tumor progression, was evaluated. Notably, downregulation of Treg cells prevalence in tumor tissues and spleen was confirmed, with Group 12 exhibiting the most pronounced reduction in Tregs from 26.7% to 3.2%, and 21.5% to 1.78%, respectively (Fig. 9e and S45). Quantitative analysis was presented in Fig. 9f. Moreover, quantification of proinflammatory cytokines, as demonstrated in Fig. 9g, revealed that IFN-α, TNF-α and IL-6 secretion reached maximal levels in Group 12, which exhibited 4.40-fold, 4.07-fold and 7.09-fold higher than those in the Group 1, respectively, indicative of a highly activated immune milieu. Figures 9 h and 9i further show the strongest CD4^+^ and CD8^+^ T-cell immunofluorescence in Group 12, confirming robust immune activation and increased T-cell infiltration. Overall, the treatment modalities acted synergistically to ablate tumors and inhibit lung metastasis. Removing any pathway weakened tumor killing, DAMP/cytokine release, and pro-inflammatory immune activation.

## Conclusion

In this study, we developed a multifunctional _T+EP_AuNP_Lu&Lap_ nanoplatform that seamlessly integrates Type I PDT, CDT, PTT, and immunomodulation to overcome and remodulate multifaceted challenges of immunosuppressive TME. By employing an electrochemical pairing of PpIX and EGCG, _T+EP_AuNP_Lu&Lap_ achieved efficient Type I PDT, characterized by sustained O_2_^•^⁻ generation and oxygen-independent activation. This effectively addressed the hypoxia-associated limitations of orthodox Type II PDT.

The inclusion of Mn^2^⁺-mediated redox cycling ensured robust •OH production, enhancing CDT. Simultaneously, Lap-driven H_2_O_2_ elevation amplified luminol chemiluminescence, triggering a more complete click cycloaddition between Tz and PpIX. This facilitated deep-tissue PTT, while further enhancing CDT and PDT efficacy.

Importantly, EGCG-enabled mitochondria-targeted delivery induced severe mitochondrial dysfunction through the massive ROS and heat generated within mitochondria. This resulted in ATP depletion, downregulation of chaperone HSP70, and enhanced ICD, which was further amplified by GSH depletion via EQ. The subsequent release of mtDNA robustly activated the cGAS–STING pathway, which in turn exacerbated ER stress through non-canonical STING–PERK signaling. In addition, factors such as ROS and heat culminated in eIF2α phosphorylation, ultimately leading to tumor cell apoptosis. This cascade promoted the release of critical DAMPs and cytokines, driving DC maturation for potent anti-gen presentation and CD8⁺ T cell activation. Crucially, this multimodal approach reprogrammed the immunosuppressive TME into a pro-inflammatory milieu, evidenced by hypoxia relief, M1-type TAM polarization, and Treg suppression. As a result, the nanoplatform achieved near-complete eradication of both primary tumors and pulmonary metastases, while inducing durable systemic antitumor immunity. Building upon the foundational proinflammatory effects demonstrated here, this study provides an instructive method for exploring the broader “cold-to-hot” tumor transition paradigm.

Overall, the _T+EP_AuNP_Lu&Lap_ nanoplatform represents a promising nano-immunotherapeutic strategy by integrating EGCG-assisted Type I PDT, Lu-triggered bio-orthogonal click aggregation of AuNPs, Lap-enhanced TME regulation, and aggregation-amplified PTT. The calculated combination index value of 0.20, which is markedly lower than 1, supports a highly synergistic interaction among the integrated therapeutic modalities. Importantly, the upregulation of p-STING and downstream immune-related factors, including p-IRF3 and IFN-β, indicates activation of the cGAS-STING signaling pathway. This localized innate immune activation further contributes to adaptive immune remodeling, including enhanced cytotoxic T-cell infiltration and reversal of the immunosuppressive TME. Nevertheless, we acknowledge that future studies using STING- or cGAS-deficient models will be valuable for definitively establishing the pathway dependency of the observed immune activation.

## Supplementary Information

Supplementary material 1

## Data Availability

No datasets were generated or analysed during the current study.
