# Supplementary material for: Self-supplying hydrogen peroxide-driven gold nanoparticle aggregation via bio-orthogonal click reaction activates cGAS-STING-PERK pathway for multimodal tumor therapy
Source: J Nanobiotechnology. 2026 Jun 27;24:873. doi: 10.1186/s12951-026-04741-3 (PMC13576277; doi:10.1186/s12951-026-04741-3)
Supplement: Supplementary file 1 [file 12951_2026_4741_MOESM1_ESM.pdf]

Supporting Information

Self-Supplying Hydrogen Peroxide-Driven Gold Nanoparticle Aggregation via Bio-orthogonal Click Reaction Activates cGAS-STING-PERK Pathway for Multimodal Tumor Therapy

LI Junyou[a], HU Kaiqi[a], LEE Man Lung[a], LI Ting[a], CHEN Pinyou[a], WANG Chengke[b,], LI Hung-Wing[a,*].*

[a] Department of Chemistry, The Chinese University of Hong Kong, Shatin, Hong Kong, China

[b] College of Food Engineering, Ludong University, Yantai, Shan Dong Province, P. R. China.

[a,*] Email: hungwingli@cuhk.edu.hk

[b,*] Email: chengkewang@163.com

1. Experimental Methods

1.1 Chemicals and Reagents

Tetrachloroauric(III) acid ($HAuCl_4 \cdot 3H_2O$, 99.9% trace metals basis), 4-formylbenzoic acid, trifluoroacetic acid (TFA, 99%), Manganese (II) chloride quadrahydrate ($MnCl_2 \cdot 4H_2O$, 99%), benzenesulfonohydrazine, aniline, sodium citrate tribasic dihydrate, sodium nitrite ($NaNO_2$,

99%), n-hexane, 1-octadecene, adenine, HS-mPEG5000, N-Hydroxysuccinimide (NHS, 98%), 5-ethynyl-2'-deoxycytidine (EDC, 97%) were purchased from Sigma-Aldrich, USA. Protoporphyrin IX (PpIX, 99.61%) and (−)-epigallocatechin-3-o-gallate (EGCG, 99.34%) were purchased from Targetmol, USA. Bovine serum albumin (BSA) and β-Lapachone (Lap, >99%) were purchased from Aladdin, China. Luminol (Lu, >99%) was obtained from Macklin, China. 3-mercaptopropionic acid were purchased from Alfa Aesar, USA. The 4T1 murine breast cancer cells were obtained from the Cell Bank of the Chinese Academy of Sciences (Shanghai, China). Antibodies were procured from Servicebio, China; Invitrogen, USA; Sanying, China, Abcam, UK. Elisa kits were purchased from Thermo Fisher Scientific, USA. All the solvents were purchased from Duksan, South Korea. DI water was used throughout the experiments.

1.2 Equipment

^{1}H-NMR spectra were conducted on Bruker AVANCE III HD 500 NMR Spectrometer. High-resolution mass spectra (HRMS) were recorded using Thermo Q Exactive Focus Orbitrap Mass Spectrometer or Bruker SolariX 9.4T ICR Mass Spectrometer. Electron spin resonance (ESR) spectra were attained with Bruker EMX-EPR Spectrometer. TEM images were recorded on FEI Tecnai G2 F20 TWIN, while elemental mapping images were acquired with Tecnai F20 high resolution microscope, and samples were prepared by dropping correspondingly diluted solutions onto carbon supported copper grids and directly observed without any staining. The UV absorbance and fluorescence emission of samples were respectively measured by Shimadzu UV3600 Plus UV-VIS-NIR Spectrophotometer and CLARIOstar Plus microplate reader or Fluorescent Spectrophotometer (Edinburgh Instruments FS5, UK). FTIR data were obtained utilizing Thermo Nicolet iS10 mid-FTIR spectrometer. Size and Zeta potential of samples were determined by Malvern Mastersizer 2000, Zeta-sizer Nano ZS90. CLSM images

were obtained on the Confocal microscope (Leica TCS SP8 X). Photothermal experiments were implemented by using a 980 nm or 808 nm infrared semiconductor laser (Blueprint, Beijing, China) and temperature change was monitored by an IR camera (Fluke TiS20+ MAX). Data of biodistribution experiments were acquitted with the inductively coupled plasma atomic emission spectrometry (ICP-AES) (ICPE-9000, Shimazu, Japan).

1.3 Methods

Preparation of 13 nm AuNP. AuNP of an average size of 13 nm was prepared according to classical sodium citrate reduction method.[1] Briefly, 20 mg of $HAuCl_4 \cdot 3H_2O$ in 50 mL of DI water was heated to boiling and then 5 mL of sodium-citrate solution (38.8 mM) were added quickly under vigorous stirring. The solution was kept boiling for another 15 min when the mixture turned wine red and was allowed to cool down to room temperature, followed by syringe filtration.

Synthesis of 2,5-diphenyltetrazole (Tz).

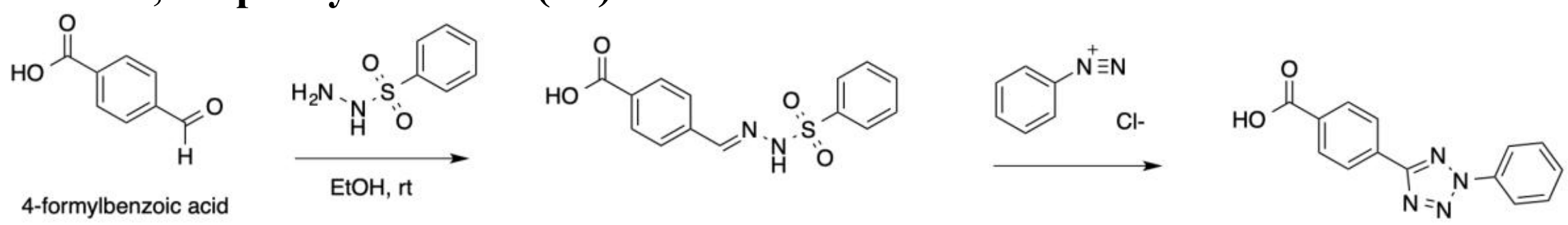

The synthesis of Tz was according to previously reported methods with modifications.[2] 4-formylbenzoic acid (1.00 g, 6 mmol) was dissolved in ethanol (100 mL), followed by addition of benzenesulfonohydrazine (1.16 g, 6 mmol). The mixture was stirred at room temperature for 1 h, then quenched with water (100 mL) and stirred for 15 min. Precipitate was filtered, washed with cold ethanol, and dissolved in pyridine (30 mL) to form solution A. Aniline was then dissolved (0.6 g, 0.60 mL, 5 mmol) in water: ethanol (1:1, 8 mL) and concentrated HCl (1.3 mL). $NaNO_2$ (0.45 g) was dissolved in water (2 mL). Both mixtures were cooled in ice bath for 5 min before addition of NaNO2 solution to aniline solution dropwise in ice bath to form solution B. Solution B was added to solution A dropwise in ice bath. Mixture was then stirred

for 1 h at room temperature. Mixture was extracted with Ethyl Acetate. 3 M HCl (250 mL) was added to combine organic layer and stirred vigorously for 10 min. Solvent was removed and then dissolved in DCM. Product was precipitated with hexanes and washed with cold hexanes to obtain red solid (0.89 g, 49.4%). The ^{1}H NMR spectrum is presented in **Figure S1**.

Preparation of BSA-functionalized AuNP (BSA-AuNP). 20 mg of SH-mPEG and 80 mg of BSA were introduced into 20 mL of aqueous dispersion of AuNP. The reaction mixture was then kept stirring overnight to modify the particle surface with BSA. The resulting BSA coated AuNP, denoted as BSA-AuNP, were purified by repetitive centrifugation (13500 rpm/30 min) to remove the uncoated SH-mPEG and BSA, and then resuspended in Milli-Q water.

Preparation of Tz-modified AuNP with Lu and Lap ($_{T}AuNP_{Lu\&Lap}$). The previously synthesized tetrazole (Tz, 3 mg), NHS (30 mg), EDC (30 mg) were first dissolved into 10 mL of DMSO. After activating for 30 minutes, 1 mL of B-AuNP solution was added into the reaction, and stirred overnight at room temperature. The reaction mixture was subjected to ultrafiltration (13500 rpm/30 min) for three times to afford the desired Tz-decorated AuNP ($_{T}AuNP$). Afterward, 50 mg of Lu was dissolved in 5 mL of 2M NaOH solution, followed by the addition of $_{T}AuNP$. The mixture was allowed for constant shaking for 12 h, which was then subjected to ultrafiltration for three times to afford $_{T}AuNP_{Lu}$. Likewise, 10 μL of 10 mg mL^{-1} Lap in DMSO was added into the above solution and kept stirring overnight, followed by ultrafiltration for three times to obtain the final $_{T}AuNP_{Lu\&Lap}$, which was resuspended in DI water, and stored in 4°C for further experiments.

Preparation of EGCG/PpIX-modified AuNP with Lu and Lap ($_{EP}AuNP_{Lu\&Lap}$). 1 mL of B-AuNP was added into 20 mg of $MnCl_2 \cdot 4H_2O$ dissolved in 5 mL of DI water and kept stirring

overnight, and was washed by ultrafiltration (13500 rpm/30 min) for three times and redispersed in 5 mL of DI water with a pH value of 8 adjusted with 0.2M NaOH aqueous solution. Subsequently, 1 mL of 10 mg/mL EGCG aqueous solution was added and the mixture was allowed for stirring for another 2 h before centrifuge and was redispersed in 1 mL of DI water. Similarly, PpIX (2 mg), NHS (20 mg), EDC (20 mg) were dissolved into 10 mL of DMSO. After activating for 30 minutes, 1 mL of B-AuNP solution was added into the reaction, and stirred overnight at room temperature. The reaction mixture was subjected to ultrafiltration (13500 rpm/30 min) for three times to afford the desired EGCG/PpIX-decorated AuNP ($_{EP}$AuNP). Afterward, 50 mg of Lu was dissolved in 5 mL of 2M NaOH solution, followed by addition of $_{T}$AuNP. The mixture was allowed for constant shaking for 12 h, which was then subjected to ultrafiltration for three times to afford $_{EP}$AuNP$_{Lu}$. Next, 10 μL of 10 mg mL^{-1} Lap in DMSO was added into the above solution and kept stirring overnight, followed by ultrafiltration for three times to obtain the final $_{T}$AuNP$_{Lu\&Lap}$, which was resuspended in DI water, and stored in 4°C for further experiments.

Electron spin resonance. The spatial control experiment utilized a standard transwell plate equipped with permeable membrane inserts. To ensure physical separation of PpIX ang EGCG during the experiment, an insert with a 250 Da MWCO dialysis membrane was employed. This prevents the diffusion and molecular mixing of PpIX and EGCG, while allowing the free exchange of dissolved O_2 and maintaining identical solvent conditions. The lower compartment was loaded with the PpIX in DMSO/H_2O solution. The upper compartment (apical insert) was loaded with EGCG solution.

Calculation of photothermal conversion efficiency (*η*). The solutions containing $_{T+EP}$AuNP, $_{T+EP}$AuNP$_{Lu}$ and $_{T+EP}$AuNP$_{Lu\&Lap}$ (200 μg mL^{-1}) were exposed to 808 nm irradiation (1.25 W

cm^{-2}) for 10 min, and then cooled down to room temperature. The temperature variation during this process was recorded by the IR camera. The photothermal conversion efficiencies (η) were measured according to the established methodology.[3]

$$\eta = \frac{\frac{mC}{\tau_s}(T_{max} - T_{ambient}) - Q_{dis}}{I(1 - 10^{-A_{808}})}$$

$$\ln(T_t - T_{ambient}) = \ln(T_{max} - T_{ambient}) - \frac{t}{\tau_s}$$

where m is the mass of the system (1 g), C is the heat capacity of water (4.2 J/g·K), τ_s is the time constant of the system (123.46 s for $_{T+EP}$AuNP, 263.16 s for $_{T+EP}$AuNP$_{Lu}$ and 232.56 s for $_{T+EP}$AuNP$_{Lu\&Lap}$), T_{max} is the equilibrium temperature of the solution, $T_{ambient}$ represents the surrounding ambient temperature (T_{max}-$T_{ambient}$ = 9.5, 31 and 45.2 °C for $_{T+EP}$AuNP, $_{T+EP}$AuNP$_{Lu}$ and $_{T+EP}$AuNP$_{Lu\&Lap}$, respectively), Q_{dis} represents heat dissipated from the laser mediated by the solvent and container (0.02 J). I is the power density of the 808 nm laser (1.25 W cm^{-2}), and A_{808} is the absorbance of nanoparticles solution at λ = 808 nm (1.32). Plug all the parameters of $_{T+EP}$AuNP, $_{T+EP}$AuNP$_{Lu}$ and $_{T+EP}$AuNP$_{Lu\&Lap}$ into the equation, the following corresponding η can be derived, respectively:

$$\eta_{\text{T+EPAuNP}} = 25.14\%$$

$$\eta_{\text{T+EPAuNP}_{\text{Lu}}} = 39.92\%$$

$$\eta_{\text{T+EPAuNP}_{\text{Lu\&Lap}}} = 68.15\%$$

Detection of ·OH generation. 3,3',5,5'-tetramethyl-benzidine (TMB) was used as an indicator to detect the generation of ·OH. First, 0.3 mL of TMB (3.6 mg mL^{-1}) solution was added to 4.3 mL of solution with H_2O_2 (200 mM) and NQO1/NADH for incubation. Afterward, 0.4 mL of aqueous dispersion (20, 50,100, 200, 500 μg mL^{-1}) of $_{P}$AuNP$_{Lu}$, $_{EP}$AuNP$_{Lu\&Lap}$, $_{EP}$AuNP$_{Lu}$ and $_{EP}$AuNP$_{Lu\&Lap}$ were subsequently added. For time-dependent detection, 200 μg mL^{-1} of AuNP

was applied and UV-vis absorbance was examined at different time points (0, 60, 120, 180, 240, 300 s). The electron-spin-resonance (ESR) spectroscopy was further used to examine the production of ·OH using 5, 5-dimethyl-1-pyrroline N-oxide (DMPO) as the trapping agent.

Detection of $^1O_2$ generation. The fluorescence singlet oxygen sensor green (SOSG) was employed to evaluate the $^1O_2$ generation of $_{P}AuNP_{Lu}$, $_{EP}AuNP_{Lu\&Lap}$, $_{EP}AuNP_{Lu}$ and $_{EP}AuNP_{Lu\&Lap}$. Briefly, a 500 μL of PBS containing AuNP (50, 100, 200, 500 μg mL^{-1}) was mixed with 500 μL of SOSG (2 μM) in Tris-HCl buffer with 200 mM of H_2O_2 and NQO1/NADH for incubation. In regard to time-dependent detection, 200 μg mL^{-1} of AuNP was applied and fluorescence was detected at different time points (0, 60, 120, 180, 240, 300 s). The ESR spectroscopy was further leveraged to examine the production of $^1O_2$ using 2,2,6,6-tetramethylpiperidine (TEMP) as the trapping agent.

Evaluation of GSH depletion ability. Aqueous suspensions of $_{P}AuNP_{Lu}$, $_{EP}AuNP_{Lu\&Lap}$, $_{EP}AuNP_{Lu}$ and $_{EP}AuNP_{Lu\&Lap}$ (20, 50, 100, 200, 500 μg mL^{-1}) were mixed with GSH (10 mM) in a PBS solution containing 200 mM of H_2O_2 and NQO1/NADH for different duration of incubation. Afterward 1 mM DTNB was added to detect the remaining GSH. The absorbance changes at 412 nm were recorded with a microplate reader. For time-dependent detection, 200 μg mL^{-1} of AuNP was applied and UV-vis absorbance was examined at different time points (0, 60, 120, 180, 240, 300 s).

Validation of $O_2^{\bullet-}$ production ability. A mixture of 158 μL of detection buffer, 1 μL of NBT and 1 μL of enzyme solution was prepared to obtain a 160 μL NBT/enzyme working solution with 200 mM of H_2O_2 and NQO1/NADH, followed by the addition of of $_{P}AuNP_{Lu}$, $_{EP}AuNP_{Lu\&Lap}$, $_{EP}AuNP_{Lu}$ and $_{EP}AuNP_{Lu\&Lap}$ with final concentrations of 20, 50, 100, 200, 500

μg mL^{-1} for incubation. Afterward, the accordingly diluted initiation solution (40X) was introduced and incubate at 37°C for 30 minutes, and then measure the absorbance at 560 nm. For time-dependent detection, 200 μg mL^{-1} of AuNP was applied and UV-vis absorbance was examined at different time points (0, 60, 120, 180, 240, 300 s).

***In vitro* aggregation verification.** The mixture of $_{T+EP}$AuNP, $_{T+EP}AuNP_{Lu}$ and $_{T+EP}AuNP_{Lu\&Lap}$ (100 μL, 2 mg mL^{-1}) in Tris-HCl buffer containing NQO1/NADH was in parallel exposed to 425 nm laser (1.0 W cm^{-2}) and chemiluminescence generated from the reaction of luminol (2 mg), H_2O_2 (200 mM) and $K_3Fe(CN)_6$ (100 mM), respectively, followed by DLS, TEM and absorption spectra measurements.

***In vitro* photothermal effect.** The aqueous solutions of aggregated $_{T+EP}$AuNP, $_{T+EP}AuNP_{Lu}$ and $_{T+EP}AuNP_{Lu\&Lap}$ at different concentrations (50, 100, 200, 500 μg mL^{-1}) were exposed to continuous irradiation by 808 nm laser with a power density of 1.25 W cm^{-2} for 10 min. Meanwhile, the aqueous solution of $_{T+EP}AuNP_{Lu\&Lap}$ (200 μg mL^{-1}) was treated with continuous irradiation by 808 nm laser with different power density (0.5, 0.75, 1.0, 1.25, 1.5 W cm^{-2}) for 10 min. Then the irradiation was turned off and the temperature cooling curve was continuously recorded and plotted. For the thermal stability test, the cycle was carried out for 3 consecutive times. In a typical experiment, 1 mL reaction aqueous solution (200 μg mL^{-1}) was irradiated by 808 nm laser (1.25 W cm^{-2}) followed by cooling to room temperature naturally.

Cytotoxicity assay. To evaluate the cytotoxicity effect of AuNPs *in vitro*, murine breast carcinoma cell line 4T1 and fibroblast cell line L929 were cultured in DMEM medium, supplemented with 10% fetal bovine serum and 2% penicillin streptomycin at 37°C in a humidified atmosphere of 5% CO_2 (DMEM + 10% FBS + 2% Penicillin medium, or

abbreviated as the medium hereafter). 4T1 and L929 cells were planted respectively in 96-well microplate with a concentration of 5×10^3 cells per well. After 24 h of growing, the cells were incubated with different concentrations of $_{T+EP}$AuNP, $_{T+P}$AuNP$_{Lu}$, $_{T+EP}$AuNP$_{Lu}$, $_{T+P}$AuNP$_{Lu\&Lap}$ and $_{T+EP}$AuNP$_{Lu\&Lap}$ (0, 10, 50, 100, 200 μg mL^{-1}), accompanied by 808 nm laser irradiation (1.25 W cm^{-2}) in 2 h for NIR groups. After further 24 h growing, cell viability was determined by MTT cell proliferation cytotoxicity assay kit.

Synergy coefficient calculation. To quantify the synergistic interactions among the integrated therapeutic modalities, the Combination Index (*CI*) was evaluated using the Chou-Talalay method:

$_{T+EP}$AuNP: $IC_{50(1)} = 100 + \frac{50-92.8}{90.7-92.8} \times (200-100) = 2138.1\ \mu g/mL$

$_{T+P}$AuNP$_{Lu}$: $IC_{50(2)} = 100 + \frac{50-92.1}{88.5-92.1} \times (200-100) = 1269.4\ \mu g/mL$

$_{T+EP}$AuNP + NIR: $IC_{50(3)} = 100 + \frac{50-80.3}{72.8-80.3} \times (200-100) = 504.0\ \mu g/mL$

$_{T+EP}$AuNP$_{Lu\&Lap}$ + NIR: $IC_{50(all)} = 50 + \frac{50-46.7}{28.1-46.7} \times (100-50) = 61.6\ \mu g/mL$

Using the mutually non-exclusive Chou-Talalay equation:

$$CI = \sum_{i=1}^{n} \frac{IC_{50(all)}}{IC_{50(n)}} + \frac{IC_{50(all)}^{n}}{\prod_{i=1}^{n} IC_{50(n)}} = \frac{61.6}{2138.1} + \frac{61.6}{1269.4} + \frac{61.6}{504.0} + \frac{61.6^3}{2138.1 \times 1269.4 \times 504.0} = 0.20$$

As this value is significantly less than 1, it demonstrates very strong synergy among the therapeutic modalities.

***In vitro* live/dead staining experiment.** The murine 4T1 cells were cultured in DMEM + 10% FBS + 2% Penicillin medium and inoculated to 8-well plates at a concentration of 8×10^4 cells per well. After growing for 12 h, the cells were incubated with $_{T+EP}$AuNP, $_{T+P}$AuNP$_{Lu}$, $_{T+EP}$AuNP$_{Lu}$, $_{T+P}$AuNP$_{Lu\&Lap}$ and $_{T+EP}$AuNP$_{Lu\&Lap}$ (0, 10, 50, 100, 200 μg mL^{-1}), accompanied by 808 nm laser irradiation (1.25 W cm^{-2}) in 2 h for NIR groups. Cells added with equivalent

PBS were set as blank control. After incubated for another 12 h, the medium was removed. The cells were washed with PBS for twice. Fresh FBS-free medium, PI and calcein were add into each well. After incubation for another 2 h, medium was removed, the cells were washed with PBS twice. Live-dead staining images were then taken with a fluorescent microscope.

***In vitro* apoptosis assessment.** Apoptosis rates were studied using a dead cell apoptosis kit with Annexin V-FITC and PI (Thermal Fisher Scientific) through flow cytometry following the manufacturer's instructions. Cells were stained with Annexin V-FITC and PI dyes. Cells at the early stage of apoptosis are negative for PI but positive for Annexin V-FITC, whereas dead cells and late-stage apoptotic cells are stained for both PI and Annexin V-FITC. Briefly, after 4T1 cells were incubated with the medium containing $_{T+EP}AuNP$, $_{T+P}AuNP_{Lu}$, $_{T+EP}AuNP_{Lu}$, $_{T+P}AuNP_{Lu\&Lap}$ and $_{T+EP}AuNP_{Lu\&Lap}$ (50 μg mL^{-1}) for 12 h. The selected light groups of the cells were irradiated with 808 nm irradiation (1.25 W cm^{-2}) for 10 min in 2 h of incubation. After another 12 h, cells were harvested and stained with Annexin V-FITC and PI immediately before analysis by flow cytometry.

Visualization of intracellular ROS production. 4T1 cells were seeded on 8-well plates and cultivated for 24 h, and the number of cells reached 2×10^5 cells per well. After incubation with the medium containing $_{T+EP}AuNP$, $_{T+P}AuNP_{Lu}$, $_{T+EP}AuNP_{Lu}$, $_{T+P}AuNP_{Lu\&Lap}$ and $_{T+EP}AuNP_{Lu\&Lap}$ (20 μg mL^{-1}) for 4 h. The selected light groups of the cells were irradiated with 808 nm irradiation (1.25 W cm^{-2}) for 10 min, and then the medium was aspirated, washed three times with PBS solution. Afterward, fresh culture media containing 2',7'-dichlorofluorescin diacetate (DCFH-DA) was added to each well and the mixture was incubated for 20 min at 37 °C. Finally, green fluorescence images were observed by a fluorescence microscope.

Detection of intracellular $^{1}O_2$ generation. 4T1 cells were seeded on 8-well plates and cultivated for 24 h, and the number of cells reached 2×10^5 cells per well. Then, cells were rinsed for three times by PBS and incubated with SOSG (20 μM) PBS solutions for 2 h. After incubation with the medium containing $_{T+EP}$AuNP, $_{T+P}$AuNP$_{Lu}$, $_{T+EP}$AuNP$_{Lu}$, $_{T+P}$AuNP$_{Lu\&Lap}$ and $_{T+EP}$AuNP$_{Lu\&Lap}$ (20 μg mL^{-1}) for 4 h, accompanied by 808 nm laser irradiation (1.25 W cm^{-2}) in 2 h for NIR groups, cells were rinsed for three times by PBS. Finally, fluorescence images were observed by a fluorescence microscope.

Detection of intracellular $O_2^{\bullet-}$ production. 4T1 cells were seeded on 8-well plates and cultivated for 24 h, and the number of cells reached 2×10^5 cells per well. Then, cells were rinsed for three times by PBS and incubated with DHR 123 (20 μM) PBS solutions for 2 h. After incubation with the medium containing $_{T+EP}$AuNP, $_{T+P}$AuNP$_{Lu}$, $_{T+EP}$AuNP$_{Lu}$, $_{T+P}$AuNP$_{Lu\&Lap}$ and $_{T+EP}$AuNP$_{Lu\&Lap}$ (20 μg mL^{-1}) for 4 h, accompanied by 808 nm laser irradiation (1.25 W cm^{-2}) in 2 h for NIR groups, cells were rinsed for three times by PBS. Finally, fluorescence images were observed by a fluorescence microscope.

Detection of the mitochondrial membrane potential (MMP). The variation of MMP was measured by JC-1 assay kit (Beyotime Biotechnology Co., Ltd.). 4T1 cells were seeded on 8-well plates and cultivated for 24 h, and the number of cells reached 2×10^5 cells per well. After incubation with the medium containing $_{T+EP}$AuNP, $_{T+P}$AuNP$_{Lu}$, $_{T+EP}$AuNP$_{Lu}$, $_{T+P}$AuNP$_{Lu\&Lap}$ and $_{T+EP}$AuNP$_{Lu\&Lap}$ (50 μg mL^{-1}) for 4 h. The selected light groups of the cells were irradiated with 808 nm irradiation (1.25 W cm^{-2}) for 10 min. Afterward, 4T1 Cells were incubated with 0.5 mL of JC-1 staining solution (8 mL of ultrapure water, and 50 μL of JC-1 (200X) and 2 mL of JC-1 staining buffer (5X) were homogeneously mixed) for 20 min. Cells were washed by JC-1 buffer solution, and fluorescence images were obtained with a fluorescence microscope.

Evaluation of intracellular O_2 generation. 4T1 cells were vaccinated in 8-well plates, incubated for 24 h at a density of 2 × 10^5 cells per well and treated with $[Ru(dpp)_3]Cl_2$ (30 μM) for 4 h, followed by incubation with 2 mL of fresh medium containing $_{T+EP}$AuNP, $_{T+P}$AuNP$_{Lu}$, $_{T+EP}$AuNP$_{Lu}$, $_{T+P}$AuNP$_{Lu\&Lap}$ and $_{T+EP}$AuNP$_{Lu\&Lap}$ (50 μg mL^{-1}) for 4 h, followed by 808 nm irradiation (1.25 W cm^{-2}) for 10 min, and the red fluorescence image was observed with a fluorescence microscope.

Detection of intracellular GSH depletion. 4T1 cells were seeded on 8-well plates and cultivated for 24 h, and the number of cells reached 2 × 10^5 cells per well, after which, the cells were treated with $_{T+EP}$AuNP, $_{T+P}$AuNP$_{Lu}$, $_{T+EP}$AuNP$_{Lu}$, $_{T+P}$AuNP$_{Lu\&Lap}$ and $_{T+EP}$AuNP$_{Lu\&Lap}$ (50 μg mL^{-1}), ensued by 808 nm irradiation (1.25 W cm^{-2}) for 10 min for NIR groups. After being cultured for another 12 h, the media were removed, and a GSH assay kit (Beyotime Biotechnology Co., Ltd.) was used to detect the intracellular amount of GSH and CLSM images were taken on a fluorescence microscope.

Measurement of extracellular ATP level. To measure the changes of extracellular ATP level, 4T1 cells were seeded in an 8-well plate at a density of 2.0 × 10^5 cells per well in DMEM + 10% FBS + 2% Penicillin medium and cultured for 24 h. Then, the medium was replaced with 500 μL of fresh medium containing $_{T+EP}$AuNP, $_{T+P}$AuNP$_{Lu}$, $_{T+EP}$AuNP$_{Lu}$, $_{T+P}$AuNP$_{Lu\&Lap}$ and $_{T+EP}$AuNP$_{Lu\&Lap}$ (50 μg mL^{-1}), followed by 808 nm irradiation (1.25 W cm^{-2}) for 10 min for NIR groups. After centrifugation, the supernatant was collected and used for measurement of the intracellular ATP level via an ATP testing kit (Beyotime Biotechnology Co., Ltd.).

Western blotting analysis. The expression levels of pertaining proteins were investigated using western blot analysis. Briefly, 4T1cells were seeded on an 8-well plate at a density of 2

× 10^5 cells per well and incubated overnight. Then the incubation media was replaced with fresh mediums including $_{T+EP}AuNP_{Lu\&Lap}$ and its counterparts(50 μg mL^{-1}). The selected light groups of the cells were irradiated with 808 nm irradiation (1.25 W cm^{-2}) for 10 min, further incubated for 12 h. Afterward, cells were collected, and the total protein was extracted. The total protein extracts were subjected to SDS-PAGE. After electrophoresis, the proteins were transferred to poly(vinylidene fluoride) (PVDF) membranes (Millipore). To block non-specific binding sites, the membranes were treated with TBST (Tris-buffered saline with Tween-20) containing 5% milk for 1.5 h. Subsequently, the membranes were incubated with the primary antibody overnight at 4 °C. After washing, the membranes were incubated with the secondary antibody. Subsequently, the expressions of HSP70 were visualized using a chemiluminescence imaging analysis system (Tanon 5200, China).

***In vitro* aggregation visualization via Bio-TEM.** To evaluate whether AuNP can effectively aggregate intracellularly in 4T1 cells, 4T1 cells were seeded in 100 mm × 20 mm dish at a density of 3 × 10^6 cells per dish in 4.0 mL of medium and cultured for 24 h. Then, the medium was replaced with 5.0 mL of fresh medium with $_{T+EP}AuNP_{Lu\&Lap}$ and its counterparts (50 μg mL^{-1}) for 6 h. After washing with PBS, 4T1 cells were treated with trypsin and then centrifuged (1000 rpm) to collect the precipitation. The obtained precipitation was fixed, dehydrated, embedded, sliced, and measured by bio-TEM.

Photothermal ablation of cancer cells. 4T1 cells were seeded in a 12-well plate at a density of 1×10^5/well, and then divided into different groups. The cells were incubated for 24 h and washed with PBS for 3 times. After removing the medium, 1 mL of DMEM + 10% FBS + 2% Penicillin medium was added to each well followed by 808 nm laser irradiation (1.25 W cm^{-2}). The cells were subsequently placed into the incubator for continuous 24 h incubation. The

upper culture medium was removed and washed with PBS. The trypsin was added to digest the adherent cells. The culture medium was added to terminate the digestion, and the cell suspension was collected and centrifuged (1000 rpm, 5 min) to remove the supernatant. The cells were washed twice with PBS, then homogenized with 400 μL binding solution. Annexin V-FITC and PI iodide were mixed and incubated at room temperature for 15 min. After that, they were placed in an ice bath away from light, and the test was completed by flow cytometry within 1 h, and the data were analyzed. Apoptosis is an active programmed cell death that can be activated by external stimulation. The degree of apoptosis was evaluated by using FITC-labelled Annexin V in the apoptotic kit in combination with phosphatidylserine from apoptotic cells. The fluorescence was detected by flow cytometry in necrotic cells stained with propidium iodide or cells that had lost membrane integrity in late apoptosis.

Mitochondria-targeting ability and aggregation via fluorescence imaging. In order to justify the mitochondria-targeting ability of $_{EP}AuNP_{Lu\&Lap}$ and its intracellular aggregation efficacy, $_{T}AuNP_{Lu\&Lap}$ and $_{EP}AuNP_{Lu\&Lap}$, along with their counterparts were used for co-localization with MitoTracker Green. 4T1 cells were maintained in the medium, incubated in 5% CO_2 at 37 °C. Afterward, the medium was replaced with FITC dyed $_{T}AuNP_{Lu\&Lap}$ and Cy5.5 dyed $_{EP}AuNP_{Lu\&Lap}$ and their counterparts (final concentration 50 μg mL^{-1}) diluted in the medium, which was co-cultured for 4 h. The cells were washed three times with PBS three times and prewarmed Mitotracker Green-containing medium was added as described in the manufacturer's guidelines, then washed with PBS three times before imaging with CLSM.

Immunogenic cell death (ICD) of 4T1 cells *in vitro*. AuNP-induced ICDs were identified by *in vitro* detection of calreticulin (CRT) transposition, HMGB1 and ATP release. To detect CRT transposition, 4T1 cells were inoculated into a 24-well plate (5×10^4 cells per well) and

incubated overnight. Then the medium was replaced with $_{T+EP}AuNP_{Lu\&Lap}$ and its counterparts (50 μg mL^{-1}) solution, and co-cultured for 24 hours. The cells were then irradiated with 808 nm laser (1.25 W cm^{-2}) for 10 min, further incubated for 4 hours, then washed twice with PBS and fixed with 4% paraformaldehyde. Cells were perforated using 0.03% triton X-100. These were followed by incubation overnight with anti-CRT antibody and HMGB1 antibody, followed by incubation with Alexa Fluor 488 (green) conjugate or Alexa Fluor 568 (red) conjugate secondary antibody for 2h. Finally, DAPI was used to stain the nuclei for 10 min, washed twice with PBS, and CLSM was used to observe. The ATP release was detected by an ATP kit. In simple terms, 4T1 cells at a density of 7 $\times 10^5$ per well at the logarithmic growth phase were seeded in a 6-well plate overnight and allowed to grow until confluence. After that, $_{T+EP}AuNP_{Lu\&Lap}$ and its counterparts (50 μg mL^{-1}) diluted with blank DMEM + 10% FBS + 2% Penicillin medium were added into each well and co-incubated for 6 hours. The cells were treated with NIR irradiation and left standing for 4 hours. The supernatant was collected directly as the sample for detection. According to the instructions of the ATP detection kit (Beyotime, Beijing, China), a standard solution and ATP detection working solution were prepared, and the ATP content in the supernatant of the samples was calculated using the standard curve drawn by the standard solution according to the results obtained from the microplate reader.

Immunofluorescence staining. Cover slides were placed in the bottom of each well of a 24-well plate, and 4T1 cells (1×10^5) in 1 mL media were added to each well and incubated at 37 °C for 12 h. Cells were then treated with PBS, $_{T+EP}AuNP_{Lu\&Lap}$ and its counterparts (50 μg mL^{-1}) for 12 h, washed with PBS, and fixed in 4% paraformaldehyde solution. Thereafter, cells were blocked with 1% BSA, penetrated by 0.1% Triton and then incubated with γ-H2AX and p-STING primary antibody at 4°C for 12 h, respectively. After washing with PBS for 3 times,

cells were incubated with Alexa Fluor 555-conjugated antibody (ab302576, Abcam) for 3 h. Nuclei were counterstained with Hoechst and then the cells were imaged with CLSM.

Mice tumor model. The mice used for experiments were allocated blindly to treatment groups. All of the relevant protocols were approved by the Animal Care and Use Committee of Laboratory Animal Center, Ludong University (ethics approval number: LDU-IRB2025030002). Adult, 6-week female Balb/c mice were used in the biodistribution assay. The mice were housed in a standard individual ventilation cages in an animal experimental system, where food and water were available ad libitum, and were kept under a 12:12-hour day-night schedule. Female BALB/c mice of 4 weeks were obtained from Beijing Vital River Laboratory Animal Technology. All animals were acclimatized for one week prior to experimentation and maintained under specific pathogen-free conditions. Mice were housed at 25 °C with 55% relative humidity and provided with unrestricted access to standard laboratory chow and water. To establish the 4T1 tumor-bearing mouse model, 5×10^5 4T1 cells suspended in PBS were subcutaneously injected into the right flank of each mouse. Approximately 10 days post-inoculation, mice with tumor volumes around 120 mm^3 were selected and subsequently subjected to the relevant experiments. For the lung metastatic cancer model, the BABL/c mice were intravenously injection with 4T1 cells (100 μL, 1×10^6 cells mL^{-1}).

***In vivo* fluorescence imaging for confirmation of AuNP aggregation and distribution.** The Cy5.5-labeled $_{(E)P}AuNP_{Lu\&Lap}$ and Cy7-labled $_{T}AuNP_{Lu\&Lap}$ and their counterparts (20 mg kg^{-1} body weight) were sufficiently mingled and intravenously injected into the tumor-bearing BALB/c mice. *In vivo* fluorescent images were taken on 0, 0.5, 1, 2, 4, 6, 8, 12, 24 h after the first injection. 24 h after the first injection, all mice were executed and dissected. Fluorescent images of main organs and tumor were taken via IVIS (Spectrum CT, PerkinElmer).

Hemolysis assay. Blood samples from healthy adult BALB/c mice were collected to evaluate the hemocompatibility of AuNP. Red blood cells (RBCs) were isolated by centrifugation and removal of the serum, followed by gentle suction. The RBCs were then washed five times with PBS and subsequently diluted tenfold with PBS. A 0.5 mL aliquot of the diluted RBC suspension was mixed with 0.5 mL of either PBS (negative control), DI water (positive control), or $_{T+EP}AuNP$, $_{T+P}AuNP_{Lu}$, $_{T+EP}AuNP_{Lu}$, $_{T+P}AuNP_{Lu\&Lap}$ and $_{T+EP}AuNP_{Lu\&Lap}$ suspensions in PBS at concentrations ranging from 20 to 1000 μg mL^{-1}. The mixtures were gently shaken and incubated at room temperature for 2 hours. After incubation, the samples were centrifuged at 10,000 rpm for 5 minutes. The supernatants were photographed, and their absorbance was measured at 576 nm. The hemolysis rate was calculated using the formula: $(A-A_0)/(A_1-A_0)\times100\%$, where A was the absorbance of sample group at 576 nm, A_1 was the absorbance of PBS group and A_0 was the absorbance of water group.

Time-dependent biodistribution. Inductively coupled plasma mass spectrometry (ICP-MS) was employed to quantify the gold (Au) content in various tissues for biodistribution analysis (n = 3). Mice were intravenously administered 20 mg kg^{-1} of $_{T+EP}AuNP$, $_{T+P}AuNP_{Lu}$, $_{T+EP}AuNP_{Lu}$, $_{T+P}AuNP_{Lu\&Lap}$, or $_{T+EP}AuNP_{Lu\&Lap}$ via the tail vein. At predetermined time points (0, 4, 12, 24, and 48 h post-injection), major organs and tumor tissues were harvested. The Au/Mn content was measured by ICP-MS to assess Au biodistribution and metabolism, particularly within the reticuloendothelial system (RES), including the liver and spleen. Each tissue sample was digested in 4.0 mL of concentrated HNO_3 overnight. Subsequently, 3.0 mL of 30% H_2O_2 was added, and the mixture was heated to approximately 200 °C until the solution volume reduced to ~0.5 mL and became transparent. The digested sample was then cooled and diluted to 3.0 g with 2% HNO_3. For quantitative calibration, a series of Au standard solutions (0.5, 1, 5, 10, 50, and 100 ppb) were prepared in 2% HNO_3 and used to generate a standard

curve. The Au concentration in each sample was determined and normalized to tissue weight per gram.

Blood Pharmacokinetic Profiles. To investigate the biological half-life of the synthesized AuNP, 4T1 tumor-bearing mice were intravenously injected with $_{T+EP}$AuNP, $_{T+P}$AuNP$_{Lu}$, $_{T+EP}$AuNP$_{Lu}$, $_{T+P}$AuNP$_{Lu\&Lap}$, or $_{T+EP}$AuNP$_{Lu\&Lap}$ (20 mg kg^{-1}). Blood samples were collected at predetermined time points (0, 1, 2, 3, 4, 5, 6, 7, 8, 9, 10, 11, 12, 24, 48, and 180 hours post-injection) following euthanasia and dissection. The collected blood samples were digested using a mixture of concentrated HNO_3 and 30% H_2O_2 (v/v = 4:1), followed by heating to remove residual acid. The digested samples were then reconstituted in 0.01 M HCl for subsequent analysis. ICP-MS was employed to quantify the concentration of Au element in the samples. A single-compartment pharmacokinetic model was applied to fit the data, and the biological half-lives ($t_{1/2}$) were determined as follows: $_{T+EP}$AuNP, $t_{1/2}$ = 3.69 h; $_{T+P}$AuNP$_{Lu}$, $t_{1/2}$ = 3.69 h; T$_{+EP}$AuNP$_{Lu}$, $t_{1/2}$ = 3.69 h; $_{T+P}$AuNP$_{Lu\&Lap}$, $t_{1/2}$ = 3.69 h; $_{T+EP}$AuNP$_{Lu\&Lap}$, $t_{1/2}$ = 3.69 h (n = 3).

***In vivo* photothermal enhancement of aggregated AuNPs.** 15 mice bearing 4T1 tumor were divided into 3 groups. When tumor volume reached 120 mm^3, mice were intravenously injected with PBS, $_{T+EP}$AuNP, $_{T+P}$AuNP$_{Lu}$, $_{T+EP}$AuNP$_{Lu}$, $_{T+P}$AuNP$_{Lu\&Lap}$, or $_{T+EP}$AuNP$_{Lu\&Lap}$ (20 mg kg^{-1} in PBS), respectively. 24 h after the injection, the tumor site of all mice was irradiated by 808 nm laser with a power density of 1.25 W cm^{-2} for 10 min continuously. Thermal images were then taken on 0, 1, 2, 5, 10 min using an infrared camera.

***In vivo* PTT experiments.** The BLAB/c mice bearing 4T1 tumors were divided into 12 groups (n = 3) and then subjected to different treatments, i.e., intravenous injection of PBS (Group 1),

PBS + NIR (Group 2), $_{T+EP}$AuNP (Group 3), $_{T+EP}$AuNP + NIR (Group 4), $_{T+P}$AuNP$_{Lu}$ (Group 5), $_{T+P}$AuNP$_{Lu}$ + NIR (Group 6), $_{T+EP}$AuNP$_{Lu}$ (Group 7), $_{T+EP}$AuNP$_{Lu}$ + NIR (Group 8), $_{T+P}$AuNP$_{Lu\&Lap}$ (Group 9), $_{T+P}$AuNP$_{Lu\&Lap}$ + NIR (Group 10), or $_{T+EP}$AuNP$_{Lu\&Lap}$ (Group 11), $_{T+EP}$AuNP$_{Lu\&Lap}$ + NIR (Group 12), with a dosage of 20 mg kg^{-1} in PBS. The power density of 808 nm laser irradiation was 1.25 W cm^{-2} and the exposure time was 10 min for NIR groups. The therapeutic effects can be clearly seen from the photographs of typical tumors extracted 20 d post-treatment. Tumor size was measured with a calliper and tumor volume was calculated with the following equation: $V = LW^2/2$, where V is the volume of the tumor, L and W are tumor length and tumor width, respectively. The tumor volume was continuously monitored till 20 d post-treatment. The tumor tissues were resected from the mice on 1 d post-treatment for evaluating the therapeutic efficacy of different treatments through hematoxylin and eosin (H&E) and terminal deoxynucleotidyl transferase-mediated dUTP nick-end labelling (TUNEL) staining, as well as Ki67 antibody. The major organ slices were stained with H&E to verify the biosafety.

Flow cytometry (FCM) analysis of the animal tissue. Tumor-bearing BALB/c mice were assigned at random (n = 3) and received $_{T+EP}$AuNP, $_{T+P}$AuNP$_{Lu}$, $_{T+EP}$AuNP$_{Lu}$, $_{T+P}$AuNP$_{Lu\&Lap}$, or $_{T+EP}$AuNP$_{Lu\&Lap}$ with or without 808 nm laser irradiation. Mice were sacrificed at day 14. For macrophage polarization analysis, tumors and spleens were excised from mice, homogenized in PBS, and filtered to obtain a single-cell suspension. The resulting cells were stained with APC-conjugated anti-F4/80, PE-conjugated anti-CD86, and PE-conjugated anti-CD206 antibodies for macrophage phenotype profiling via flow cytometry. For dendritic cell (DC) maturation analysis, the lymph nodes were harvested, homogenized in PBS, and filtered to yield a single-cell suspension. Cells were then stained with FITC-conjugated anti-CD11c, PE-conjugated anti-CD86, and APC-conjugated anti-CD80 antibodies, followed by flow

cytometric analysis to assess DC maturation. For T cell activation analysis, spleens were homogenized in PBS to generate single-cell suspensions, followed by erythrocyte lysis using a red blood cell lysis buffer. The spleen and tumor cell suspensions were then stained with FITC-conjugated anti-CD3, PE-conjugated anti-CD8a, and APC-conjugated anti-CD4 antibodies, and analyzed by flow cytometry. To evaluate regulatory T cells (Tregs), tumor and spleens were processed in True-Nuclear™ Transcription Factor Buffer Set to obtain single-cell suspensions. These were subsequently stained with FITC-conjugated anti-mouse CD4, APC-conjugated anti-CD25, and PE-conjugated anti-mouse FOXP3 antibodies for flow cytometric analysis. The cytokine levels (TNF-α, IFN-α, IL-6, IL-10 and IL-12) in the supernatant were quantified using commercial ELISA kits.

Histological and immunohistochemical analyses. Excision of tumors for histological observation was assessed by standard H&E staining and immunofluorescence (IF) staining. For H&E staining, the excised tumor were fixed in 4% paraformaldehyde solution, embedded in paraffin, sectioned and stained with hematoxylin and eosin. The sections were then observed under a fluorescence microscope. TUNEL staining was applied to evaluate apoptosis of tumor tissues, the procedures were consistent with the manufacturer's protocol, and finally examined by CLSM. For detecting the expression of HSP70, NQO1, CRT and p-STING in tumor tissues, frozen tumor sections were fixed, and blocked with 1% BSA. Then the sections were incubated with primary antibodies against each protein, respectively, overnight at 4 °C, followed by processing with corresponding second antibodies. Nuclei were counterstained with Hoechst, and then the stained sections were imaged by CLSM.

Blood Biochemistry and Hematology Examinations. BALB/c mice were intravenously injected with 20 mg kg^{-1} of AuNP of 12 groups of treatment dispersed in PBS. Blood samples

from mice were collected by eye socket bleeding after injection for 0, 1, 7, and 14 days, and then subjected to blood biochemistry and hematology examinations. (n=3)

Lung metastasis inhibition corroboration. The female tumor-bearing BALB/C mice were first injected with 100 μL of 4T1 (5×10^5 cells mL^{-1}) cells through tail veins to establish lung metastatic mice model. Then, the mice were injected intravenously with different treatment of 12 groups stated previously (20 mg kg^{-1}). On the 14th day of treatment, the lungs of the tumor-bearing mice were excised and the lung metastasis loci were calculated after performing euthanasia on the mice, and each group of mice lungs were subjected to H&E staining to illustrate the metastasis inhibition effects.

HOMO-LUMO calculation. All calculations were carried out using the Gaussian 16 software package. Geometry optimizations and single-point energy calculations were performed using the Becke, 3-parameter, Lee-Yang-Parr (B3LYP) exchange-correlation functional. The 6-311+G(d,p) basis set was employed for all atoms (C, H, O, N) to accurately describe the extended π-conjugated systems and accommodate the potential formation of anionic states. To mimic the physiological aqueous environment relevant to our biological applications, the Solvation Model based on Density (SMD) with water as the solvent was applied during all calculations. Harmonic vibrational frequency calculations were performed at the same level of theory to confirm that the optimized structures represented true energy minima.

2. Supplementary Figures

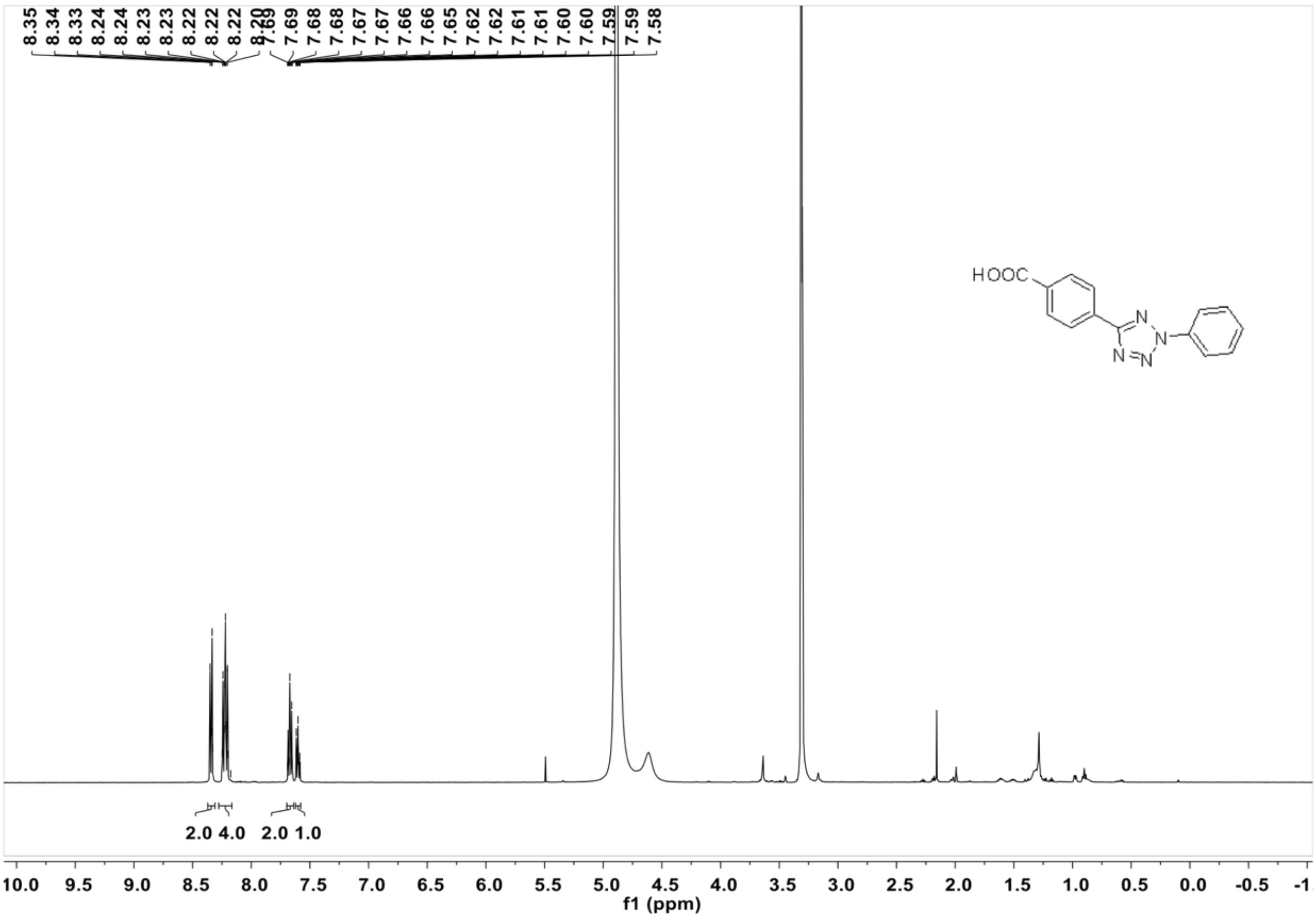

Figure S1. ^{1}H NMR spectra of the synthesized Tz. ^{1}H NMR (500 MHz, DMSO-d6), 8.31 (d, *J* = 8.1 Hz, 2H), 8.18 (t, *J* = 8.2 Hz, 4H), 7.72 (t, *J* = 7.7 Hz, 2H), 7.66 (t, *J* = 7.4 Hz, 1H), 2.09 (s, 2H).

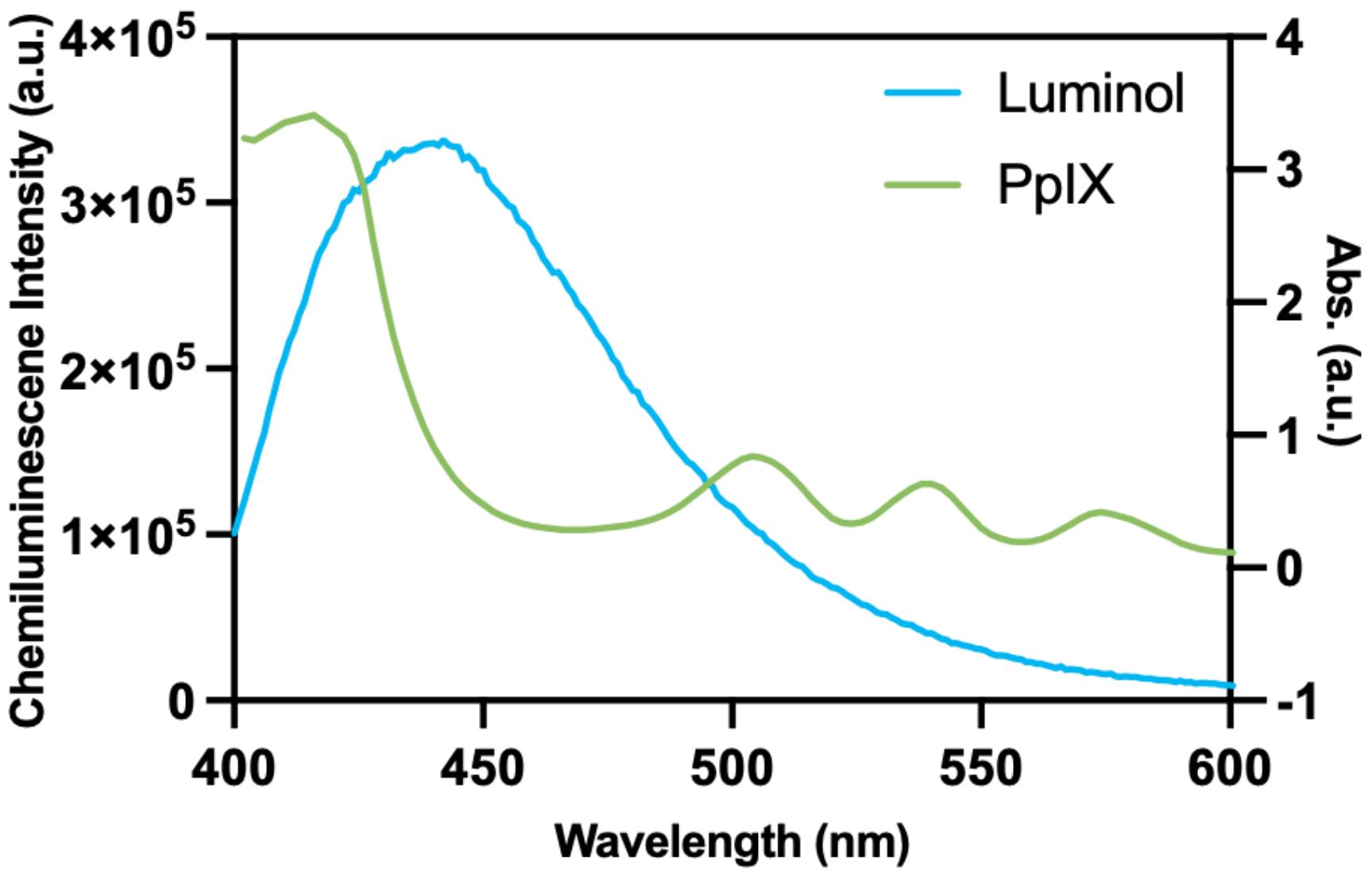

Figure S2. Luminol chemiluminescence spectra (in NaOH solution) and PpIX absorbance spectra (in DMSO), manifesting their overlapping in spectra.

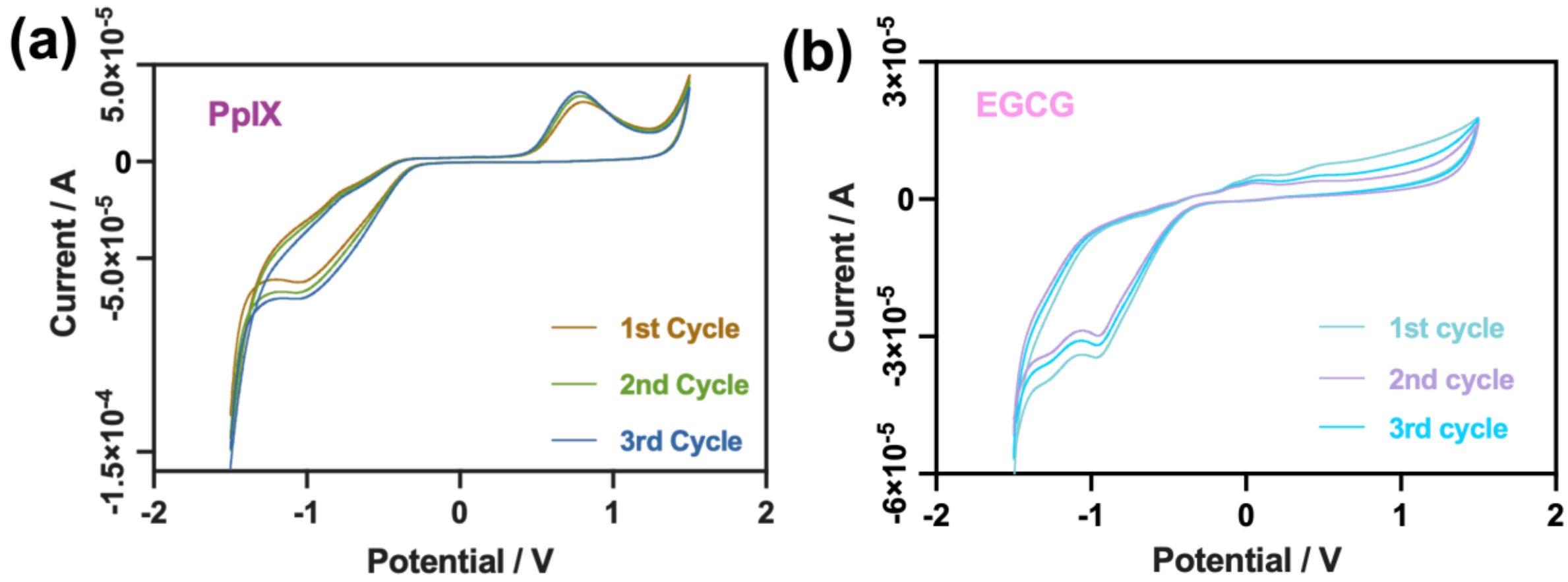

Figure S3. Cyclic voltammetry (CV) spectra of a) PpIX and b) EGCG.

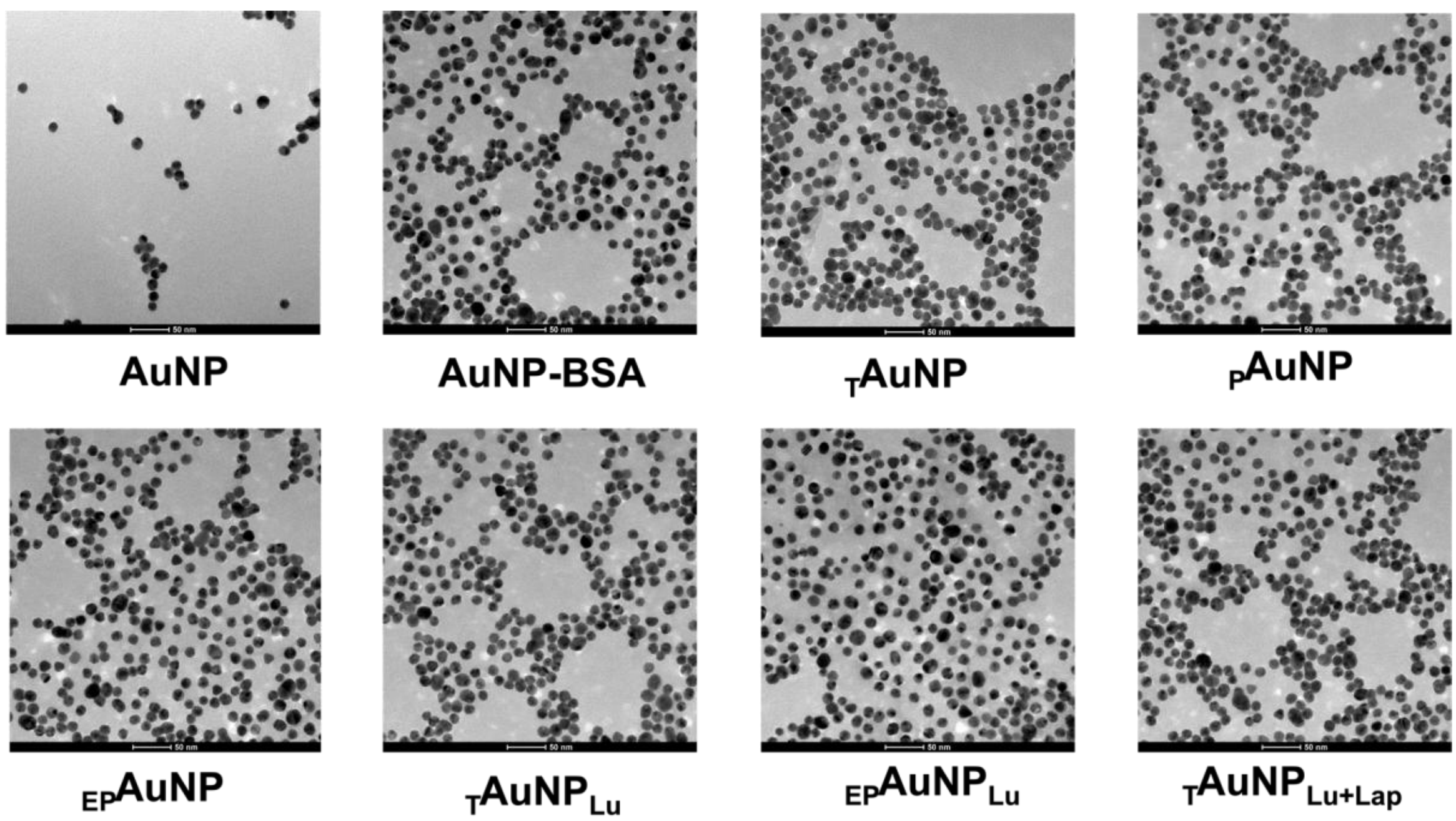

Figure S4. TEM images of AuNP at each step of progressive synthesis.

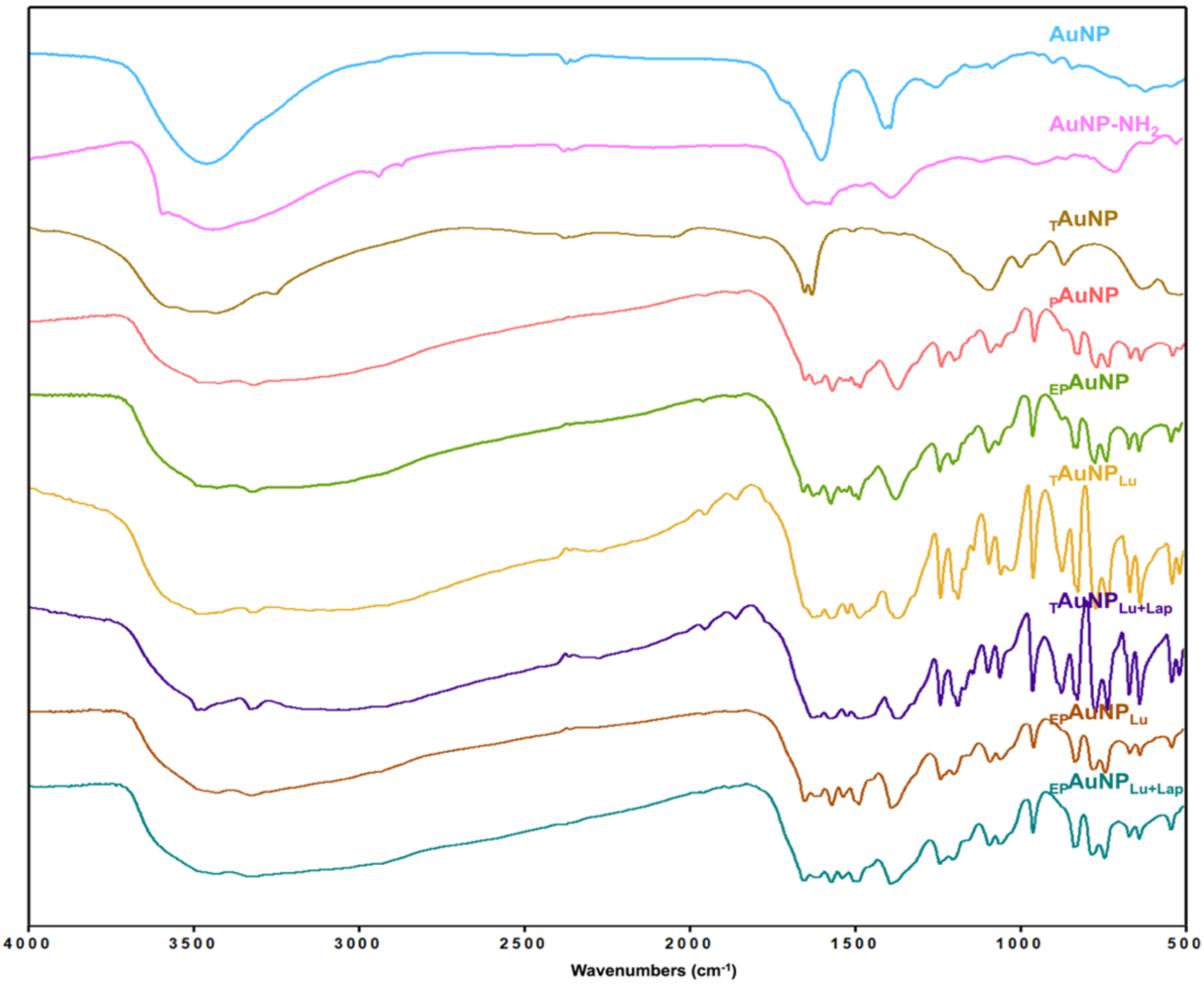

Figure S5. FTIR spectra of $_{EP}$AuNP$_{Lu\&Lap}$ and $_{T}$AuNP$_{Lu\&Lap}$ at each step of synthesis.

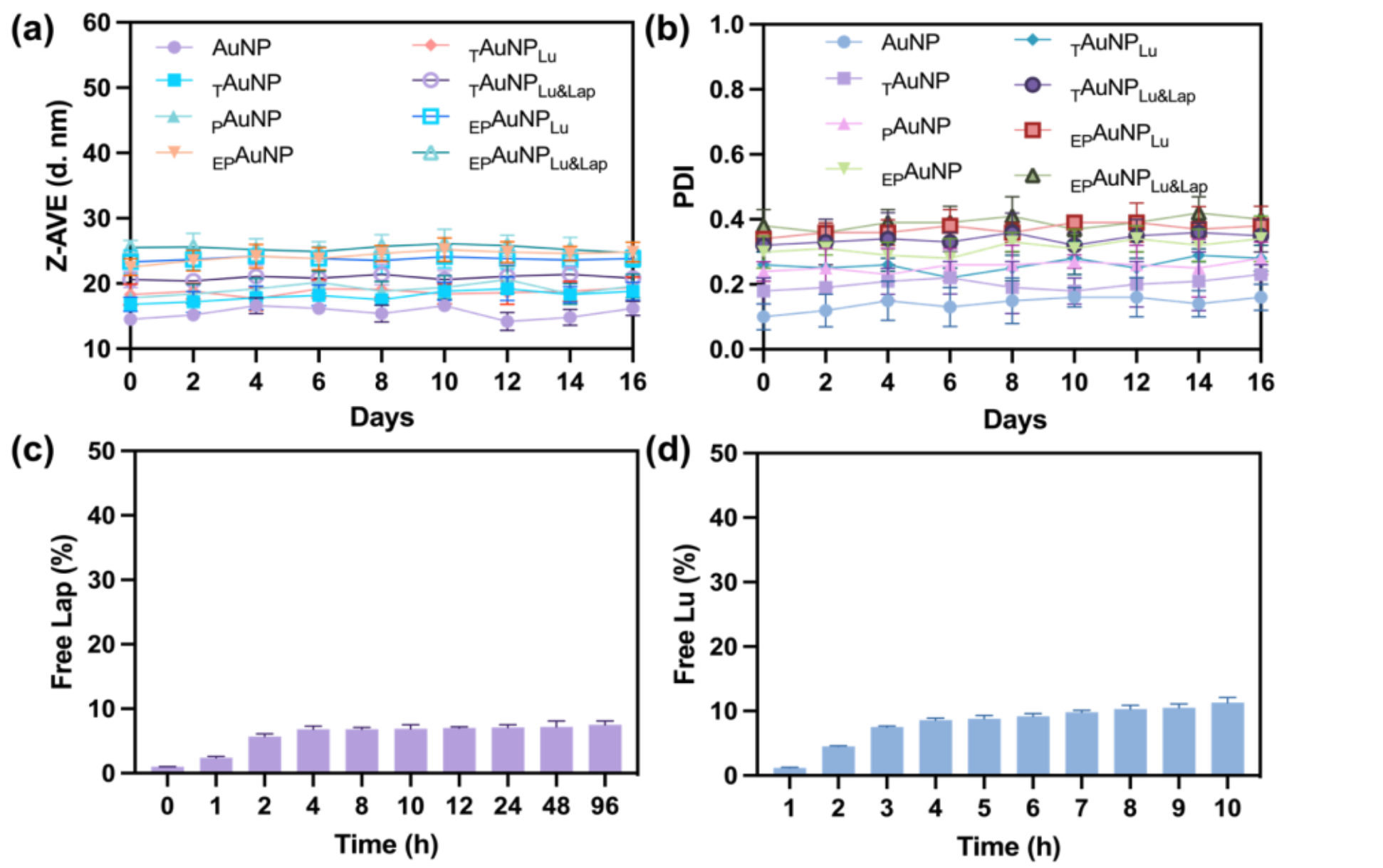

Figure S6. Evaluation of stability of $_{EP}$AuNP$_{Lu\&Lap}$ and its counterparts. a) The changes of hydrodynamic size of AuNP as days progressed. b) PDI of corresponding Z-ave in Tris-HCl buffer. c) The real-time release of Lap and d) Lu released from $_{EP}$AuNP$_{Lu\&Lap}$ over time in aqueous solution, determined by HPLC. Quantitative data are presented as mean ± SD (n=3).

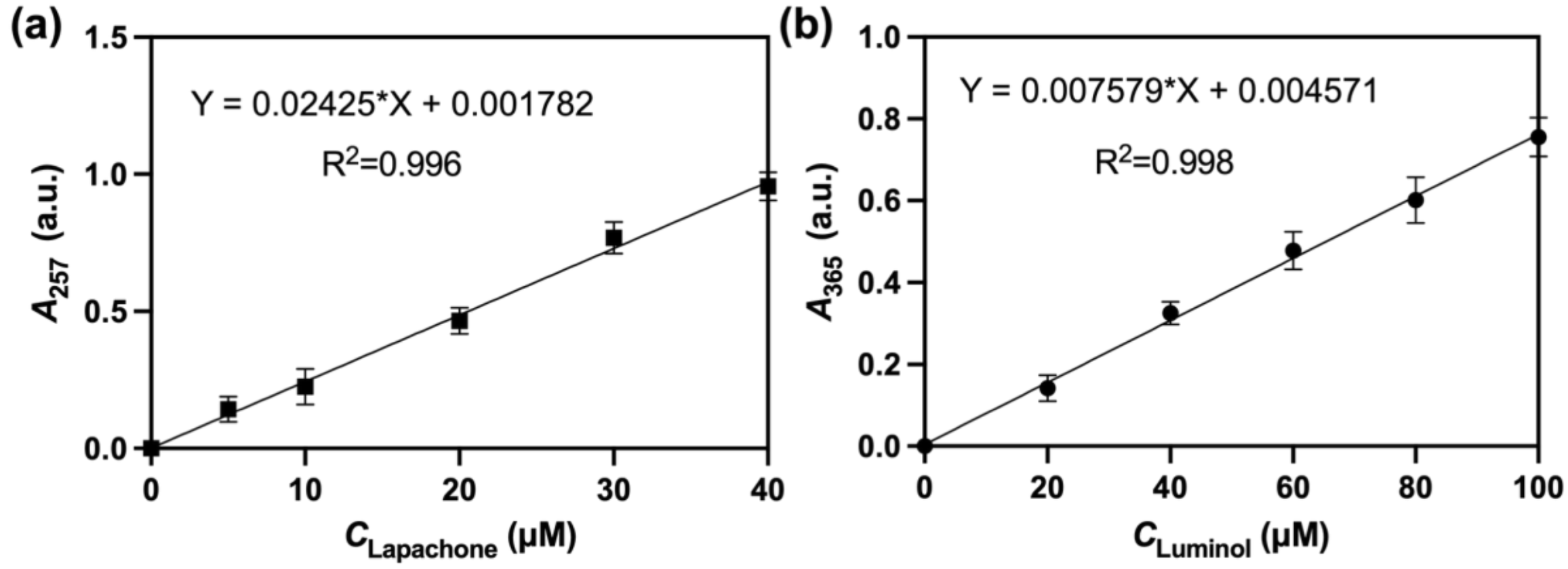

Figure S7. Standard curve for confirmation of a) Lap and b) Lu loading. (Total Lap/Lu initially added: 5.0 mg, total mass of recovered AuNP: 5.0 mg, volume of supernatant: 20.0 mL, A_{257} of free Lap = 0.85, A_{365} of free Lu = 0.45, which results in the EE_{Lap} = 66.1%, EE_{Lu} = 58.2%, and LC_{Lap} = 39.7%, LC_{Lu} = 36.8%). Quantitative data are presented as mean ± SD (n=3).

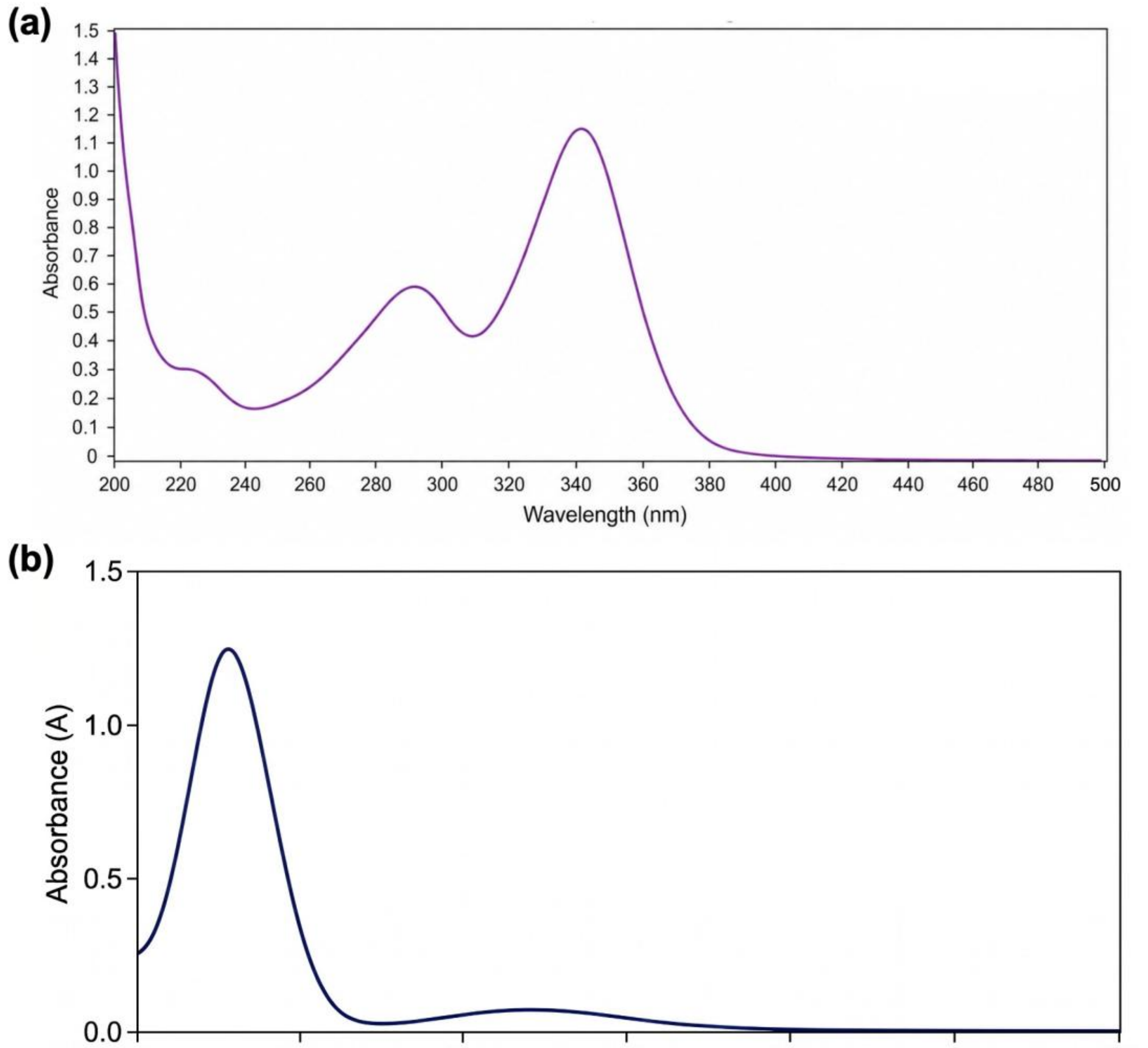

Figure S8. UV-Vis spectra of a) Lu dissolved in 0.1 M NaOH solution and b) Lap in DMSO.

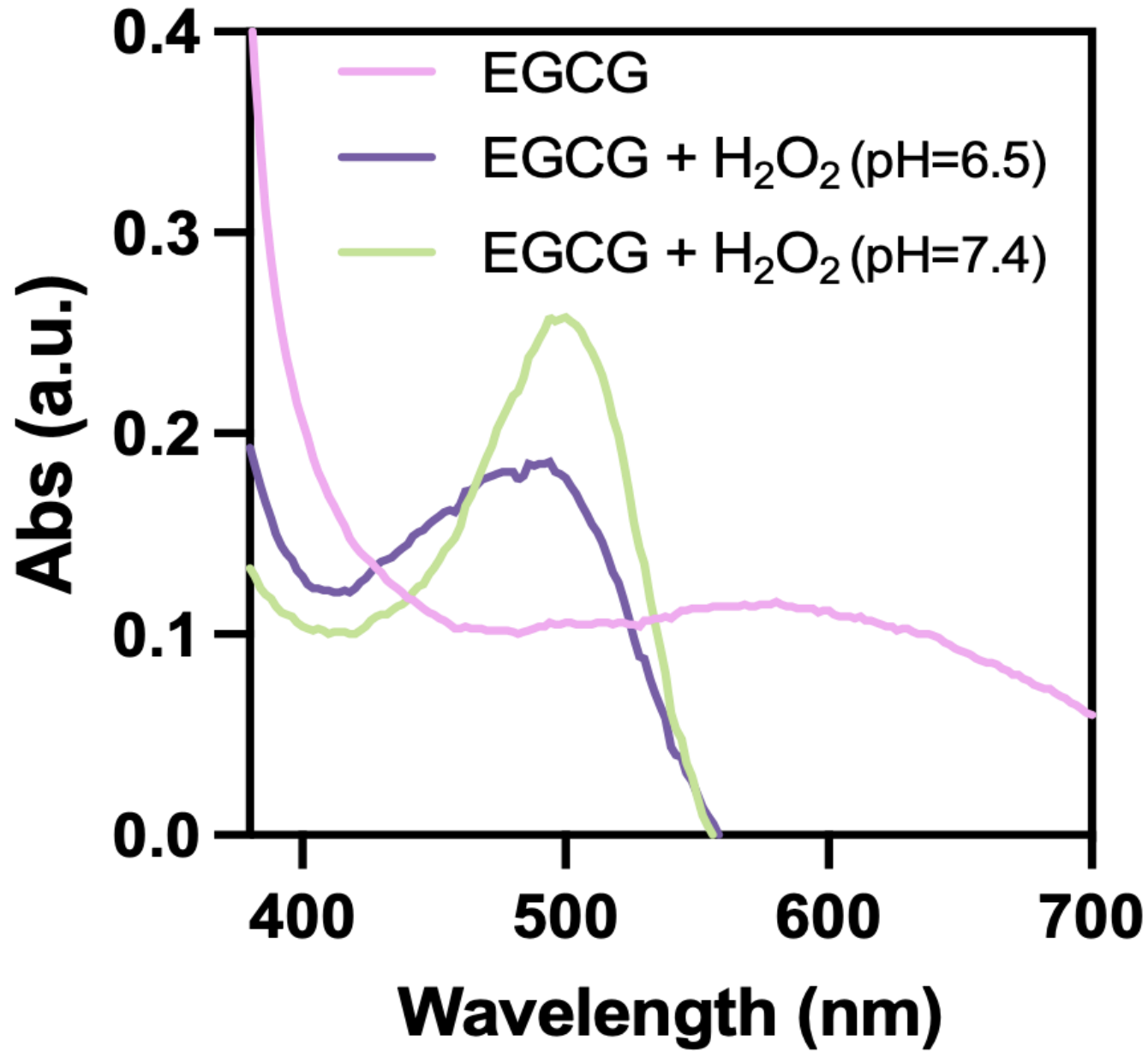

Figure S9. UV-Vis spectra of EGCG, EGCG+H_2O_2 at pH=6.5 and 7.4, respectively for verification of oxidation of EGCG under ROS.

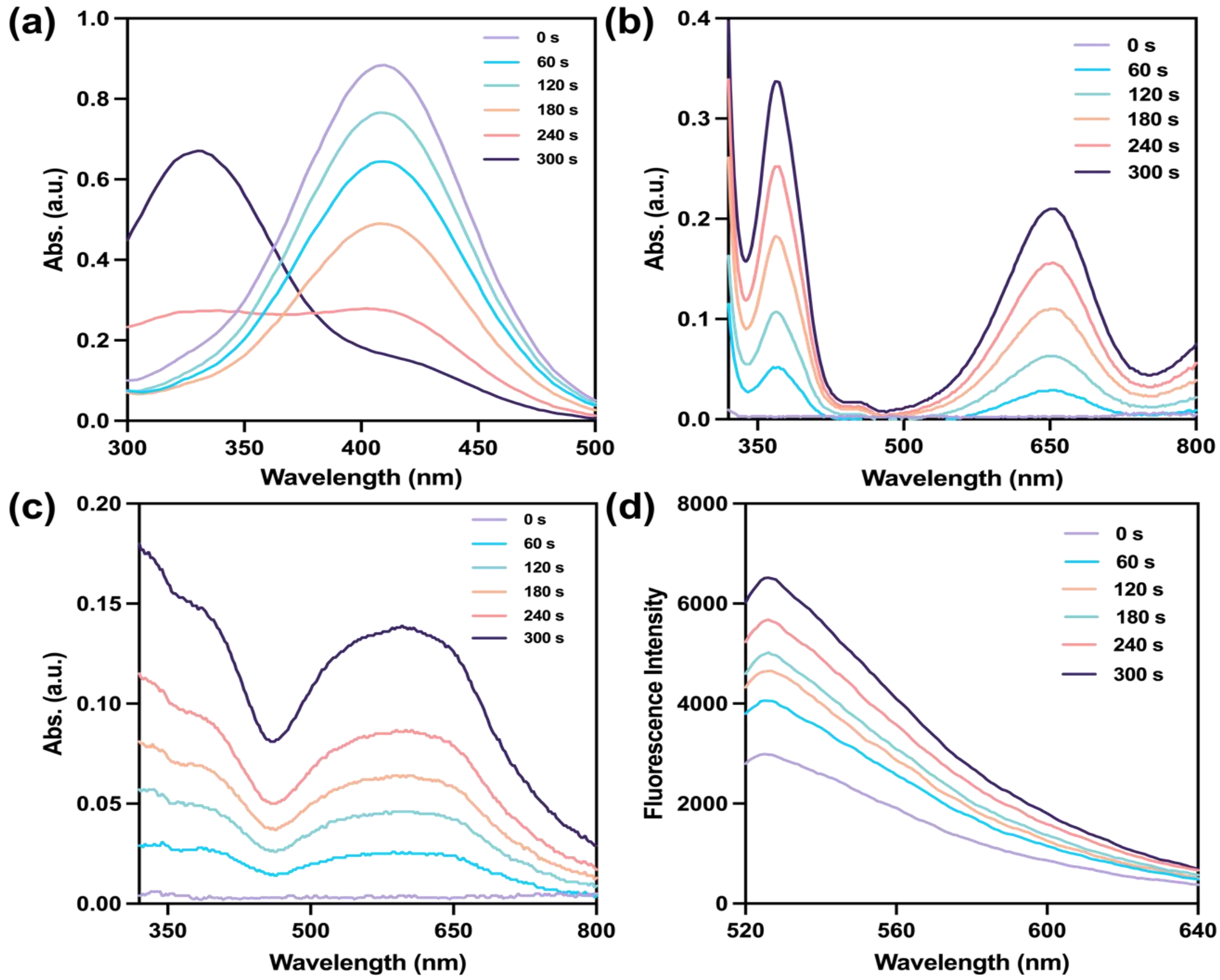

Figure S10. Time-dependent characterizations of ROS generation ability of $_{T+EP}AuNP_{Lu\&Lap}$. a) GSH depletion ability harnessing DTNB. b) ·OH generation ability via TMB oxidation. c) $O_2^{\bullet-}$ production ability by NBT. d) $^1O_2$ yielding ability using SOSG.

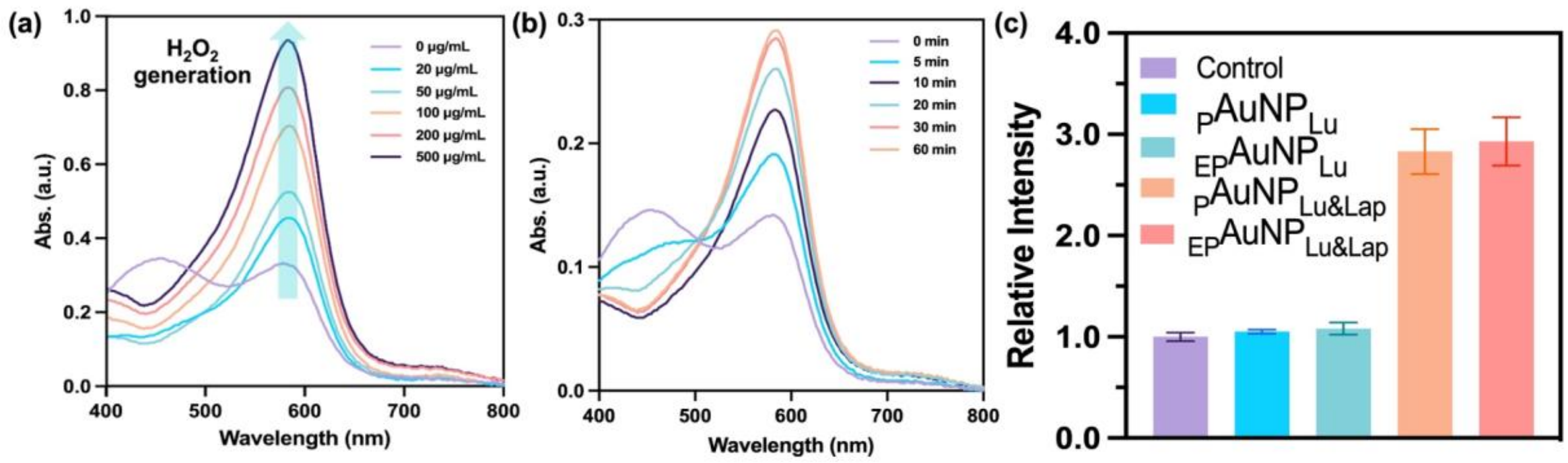

Figure S11. Evaluation of H_2O_2 generation of $_{EP}$AuNP$_{Lu\&Lap}$. a) Concentration-dependent and b) time-dependent H_2O_2 generation incubated with NQO1/NADH enzymes. c) Comparison of the H_2O_2 generation of $_{EP}$AuNP$_{Lu\&Lap}$ and its counterparts. Quantitative data are presented as mean ± SD (n=3).

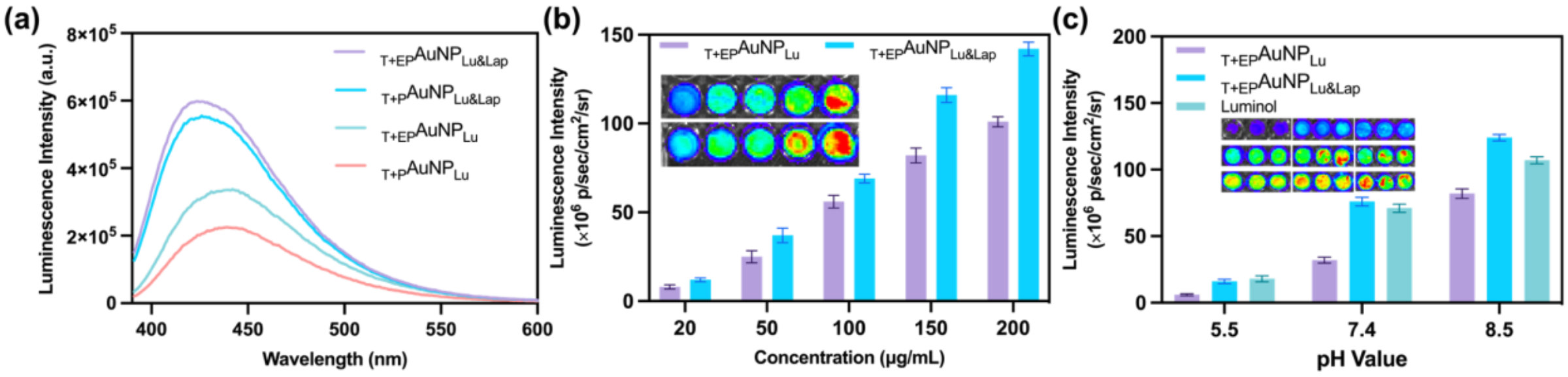

Figure S12. *In vitro* characterization of chemiluminescent properties of $_{T+EP}$AuNP$_{Lu\&Lap}$ and its comparatives. a) Luminescence spectra in the presence of 100 mM H_2O_2 and NQO1/NADH. b) Concentration-dependent changes (20, 50, 100, 150, 200 μg mL^{-1}) and c) pH-dependent (5.5, 7.4, and 8.5) chemiluminescence intensity in Tris-HCl buffer incubated with 100 mM H_2O_2 and NQO1/NADH. Quantitative data are presented as mean ± SD (n=3).

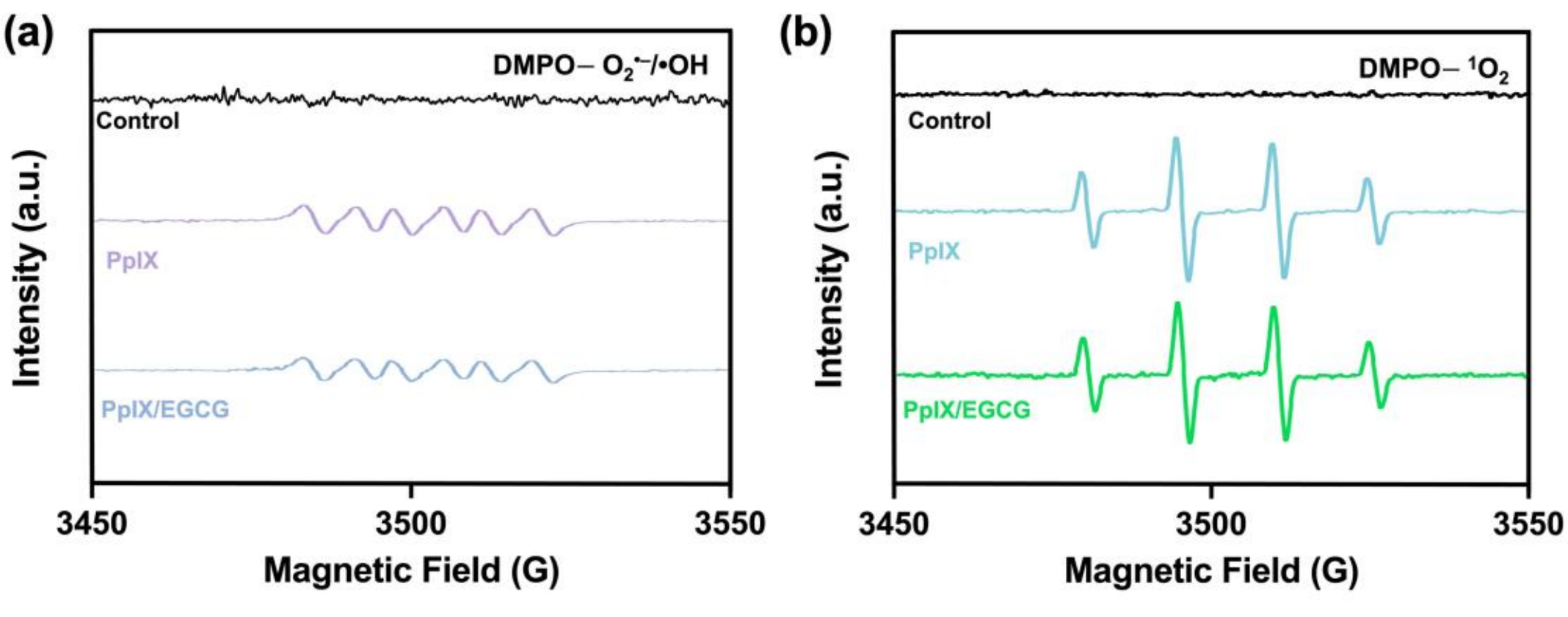

Figure S13. ESR spectra of PpIX and PpIX/EGCG for the detection of a) •OH and $O_2^{\bullet-}$ via DMPO and b) $^1O_2$ via TEMPO utilizing a standard transwell plate equipped with 250 Da MWCO dialysis membrane inserts.

(a)**MS spectra of PpIX**

Molecular formula :	$C_{34}H_{34}N_4O_4$
Experimental Mass [M-H]-:	561.24993
Theoretical Mass [M-H]-:	561.25073
Error (ppm) :	-1.4

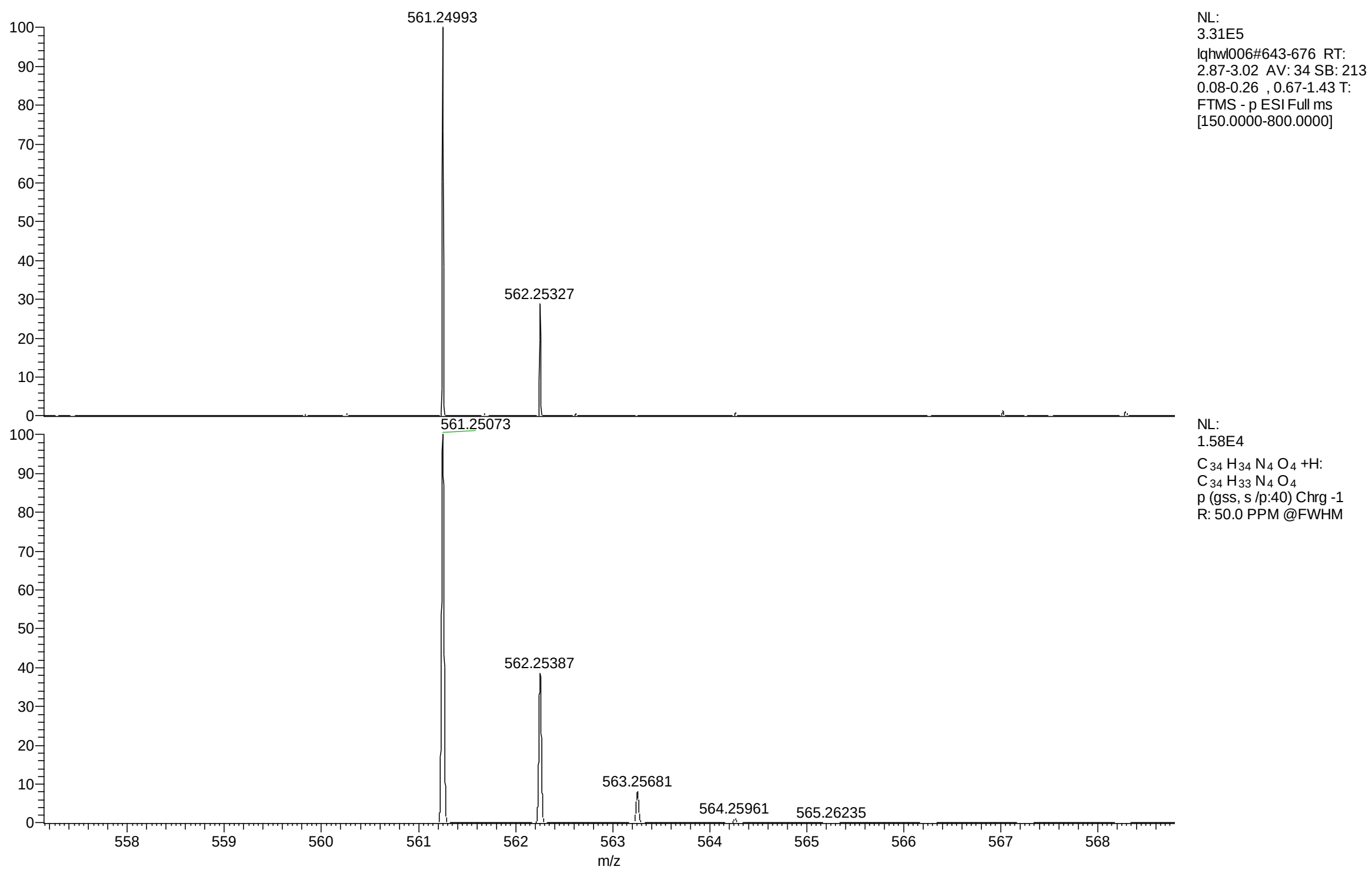

(b)**MS spectra of Tz**

Molecular formula :	$C_{14}H_{10}N_4O_2$
Experimental Mass [M-H]-:	265.07306
Theoretical Mass [M-H]-:	265.07310
Error (ppm) :	-0.2

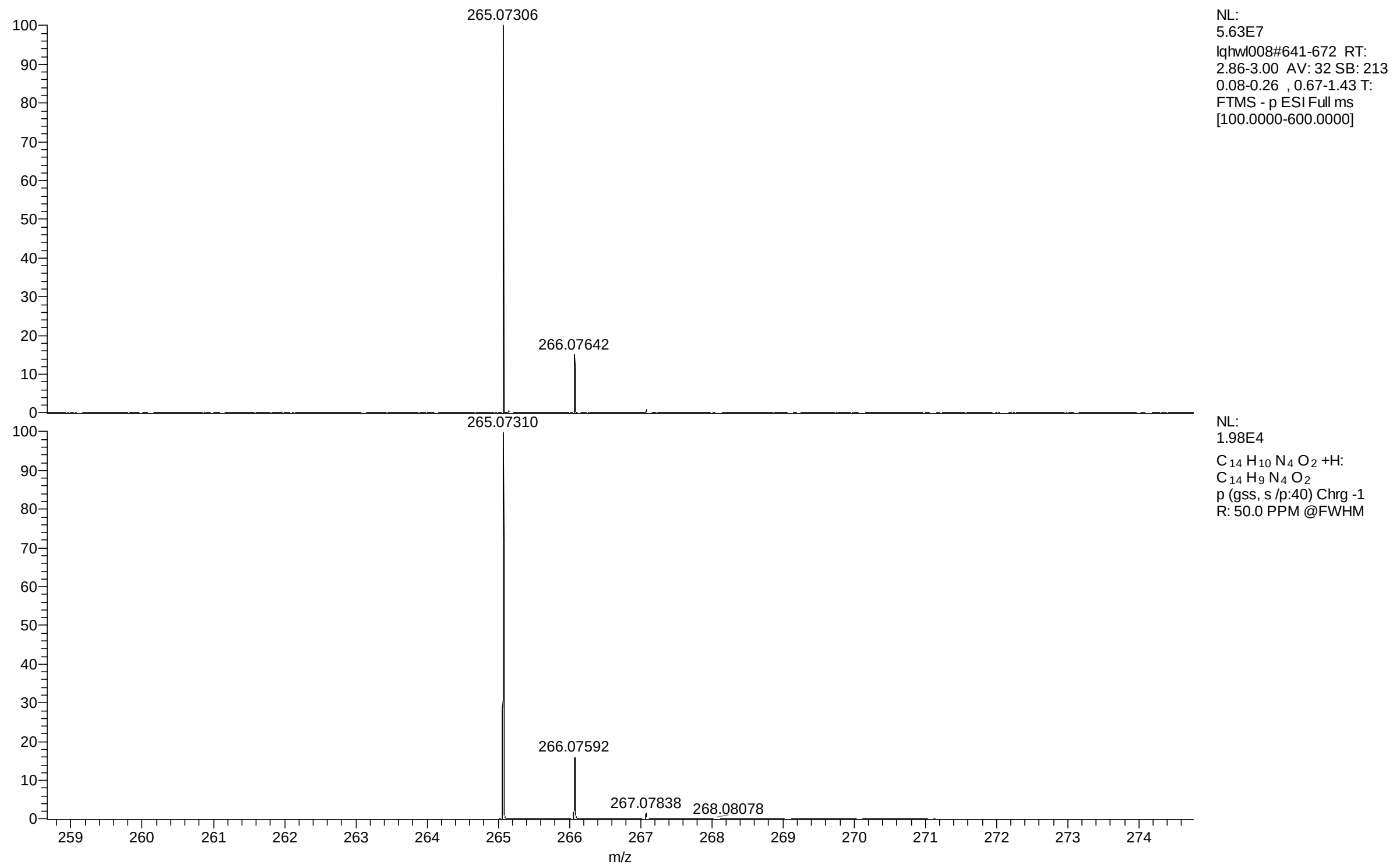

(c)**MS spectra of Tz+PpIX cycloaddition product**

Molecular formula :	$C_{48}H_{44}N_6O_6$ / $C_{62}H_{54}N_8O_8$
Experimental Mass [M-H]-:	799.32624 / 1040.41834
Theoretical Mass [M-H]-:	799.32496 / 1040.41686
Error (ppm) :	1.6 / 1.4

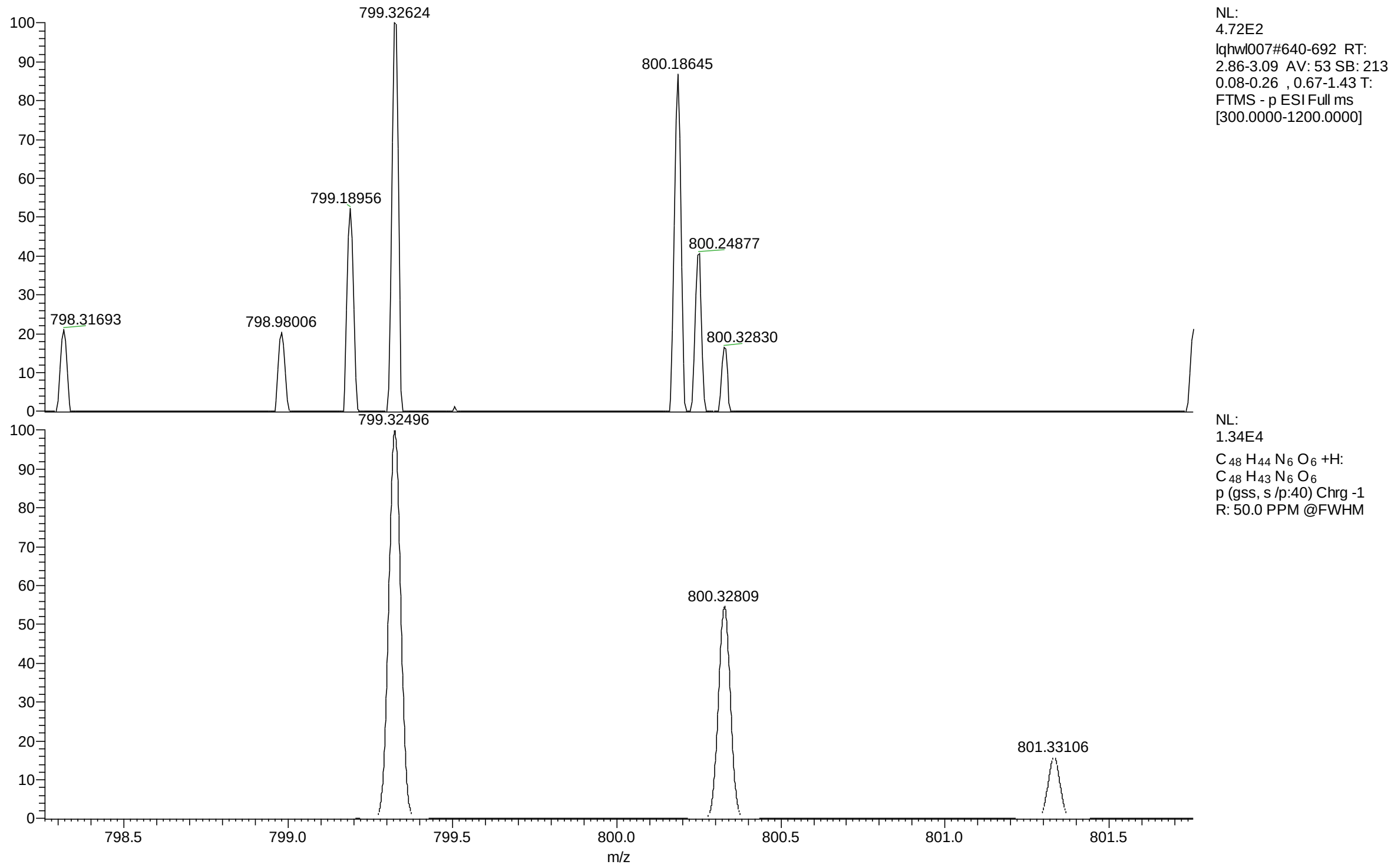

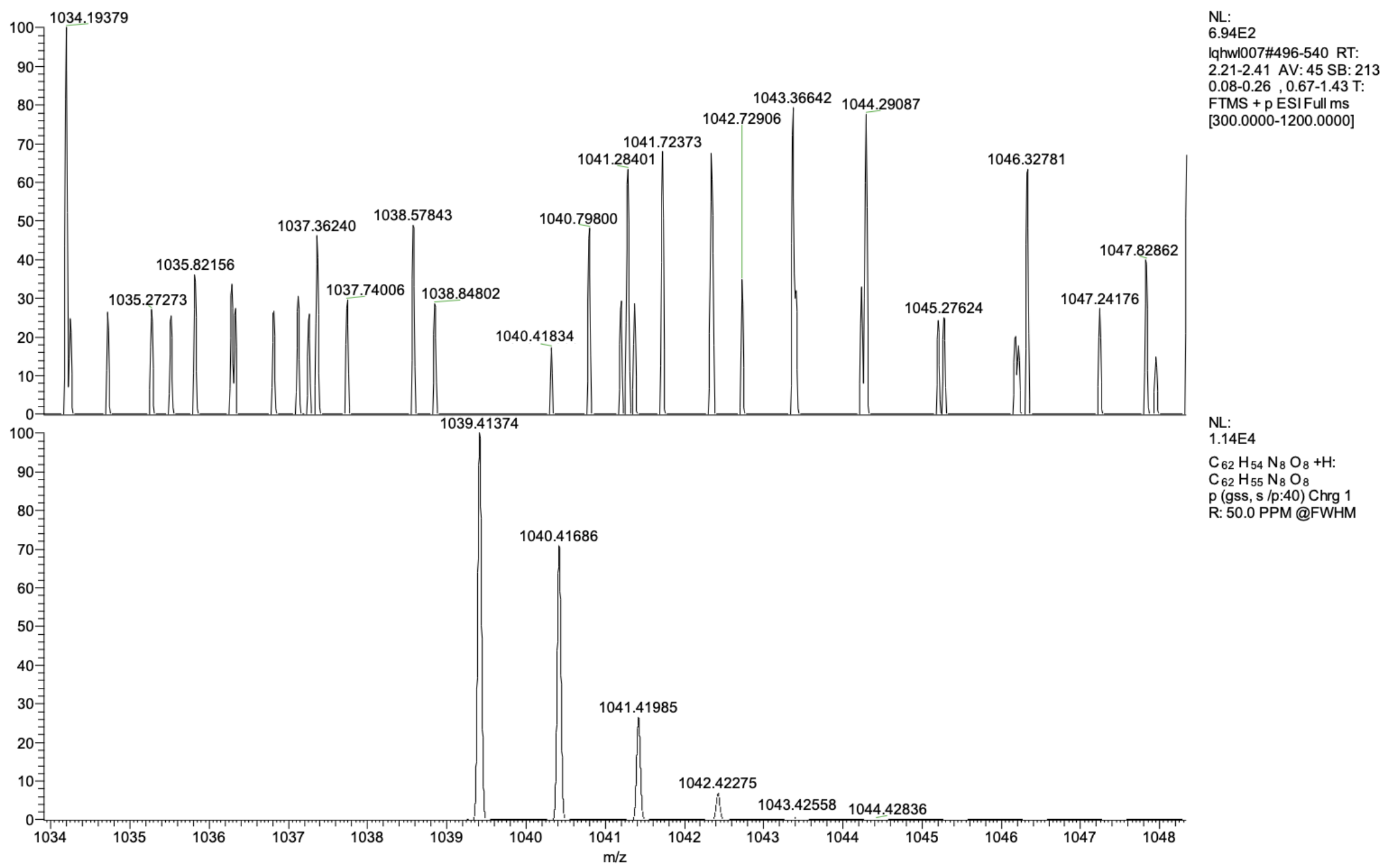

Figure S14. MS spectra for corroboration of cycloaddition between Tz and PpIX under laser irradiation. MS spectra of a) PpIX, b) Tz and c) Tz+PpIX irradiated by 425 nm laser with a power intensity of 0.8 W cm^{-2} for 15 min.

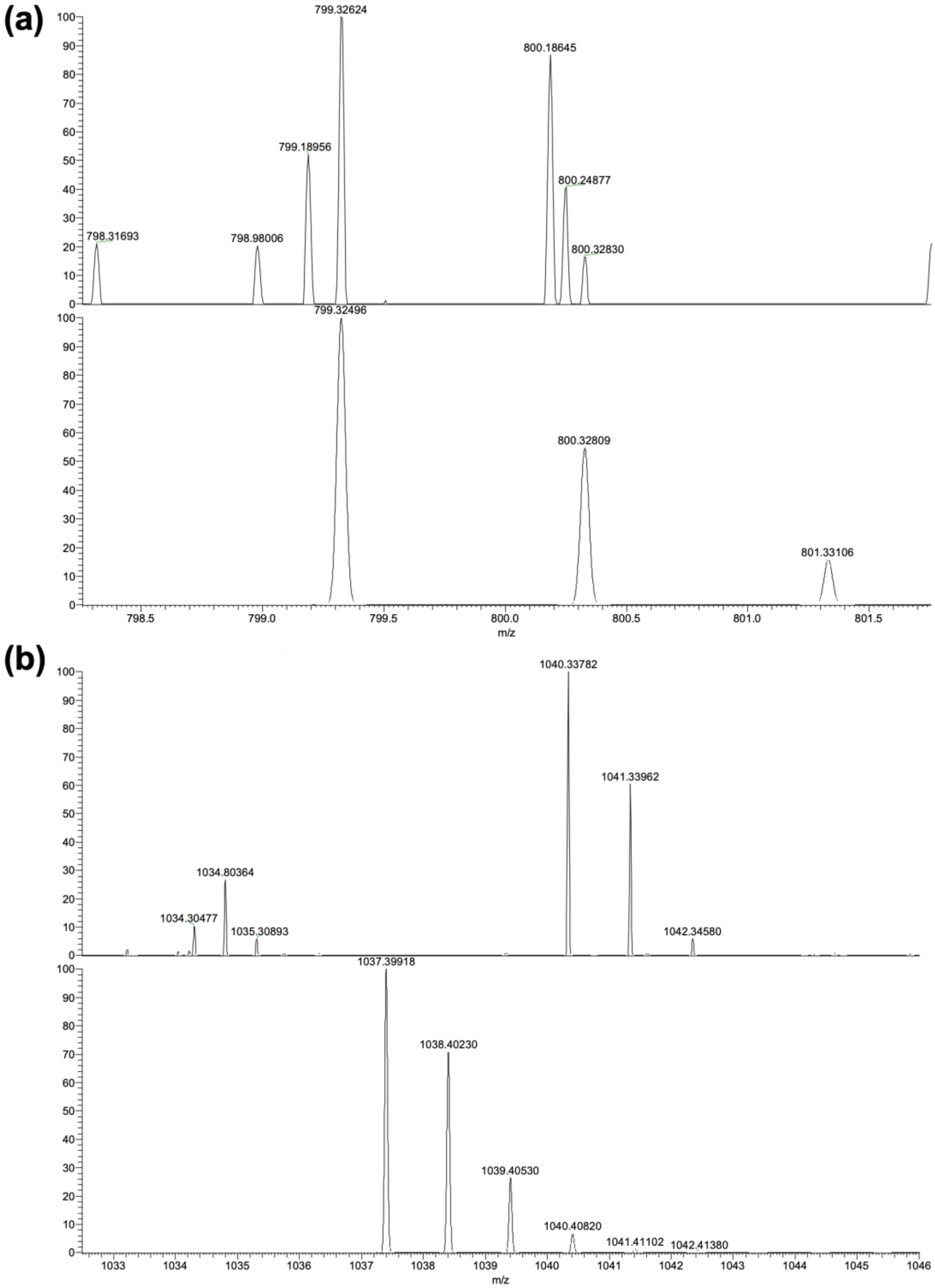

Figure S15. MS spectra of Tz+PpIX for corroboration of cycloaddition between Tz and PpIX under 425 nm laser irradiation with a power intensity of 0.8 W cm^{-2} for 15 min in cellular lysate.

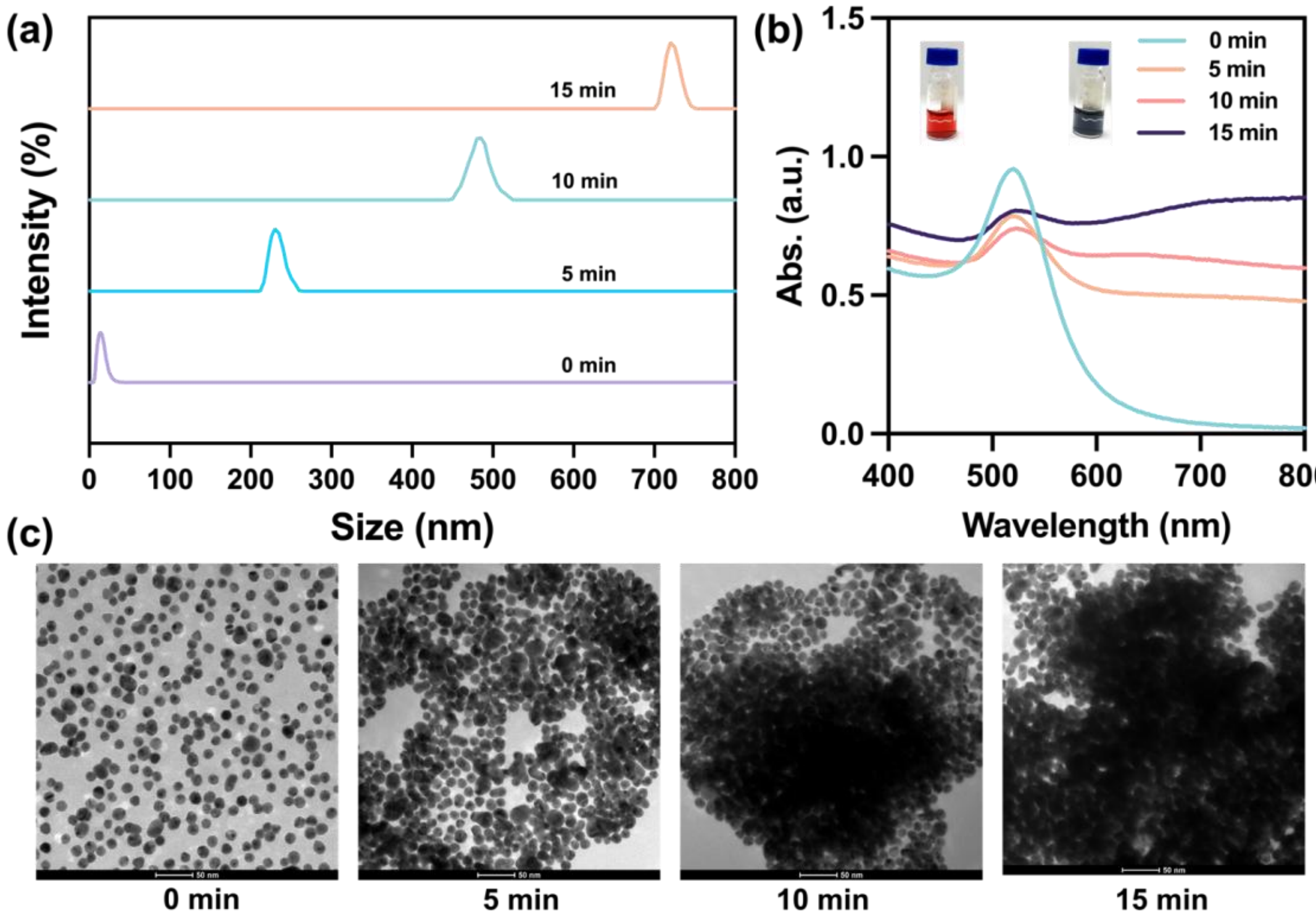

Figure S16. Verification of aggregation behavior of $_{T+EP}$AuNP irradiated by 425 nm laser (0.8 W cm^{-2}). a) DLS analysis, b) corresponding UV-Vis spectra, and c) TEM images of $_{T+EP}$AuNP under laser irradiation at different time points.

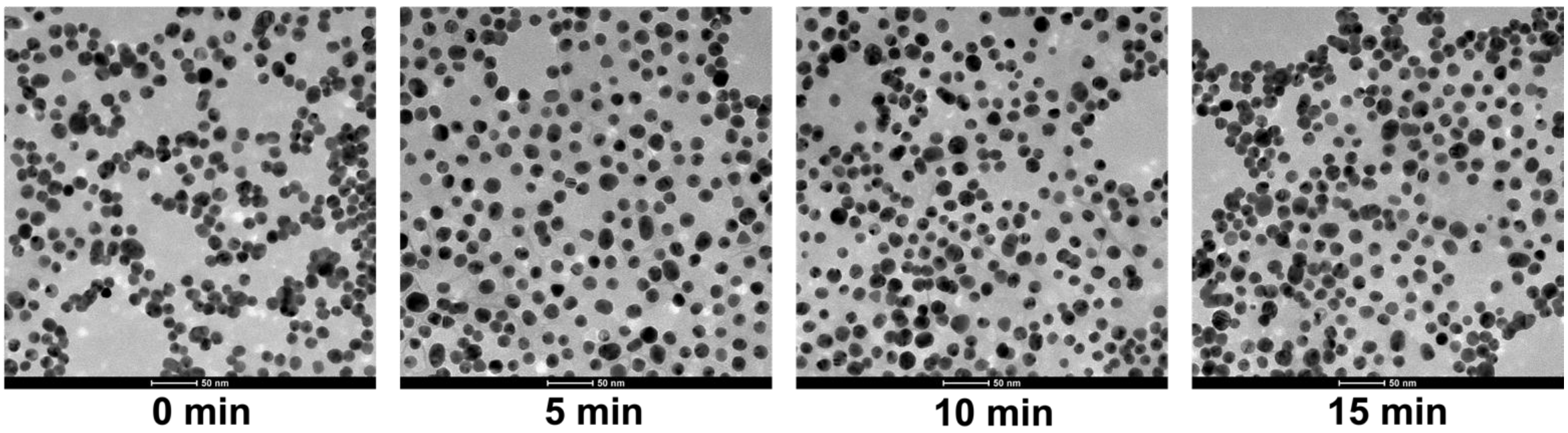

Figure S17. TEM images at different time points of $_{T+EP}$AuNP without 425 nm laser irradiation, demonstrating no visible aggregation over time.

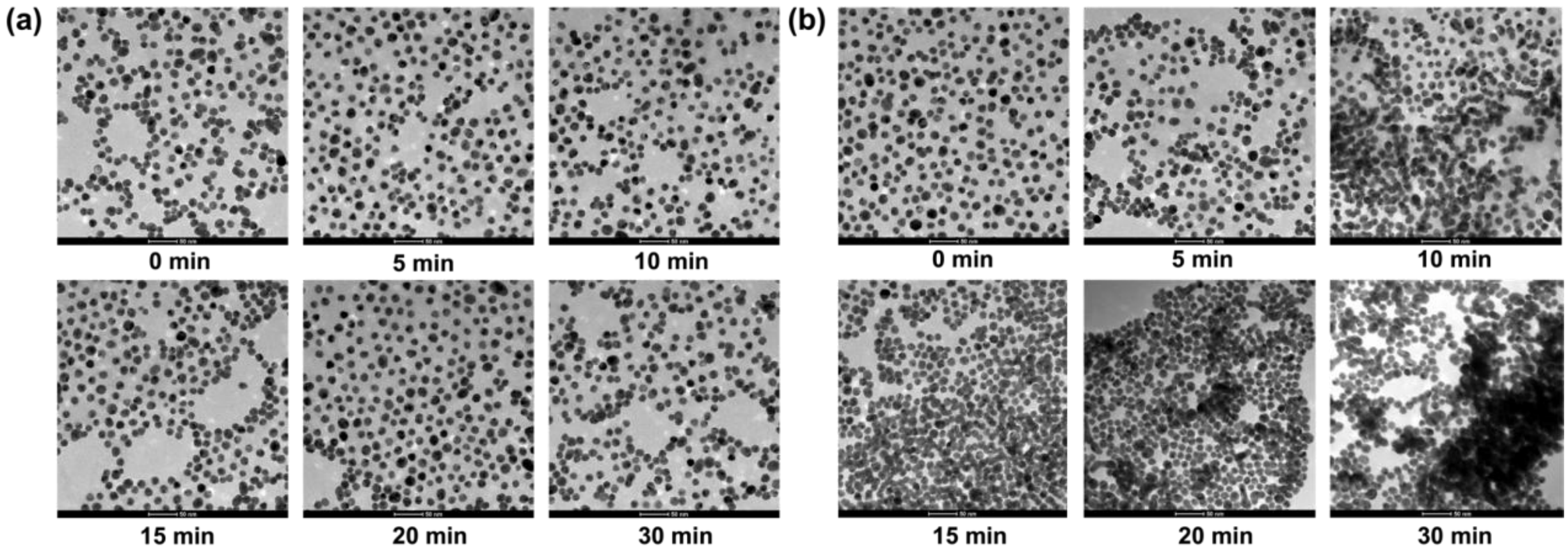

Figure S18. Feasibility confirmation of aggregation of nanoplatform induced by Lu chemiluminescence. TEM images of a) $_{T+EP}AuNP_{Lu}$ and b) $_{T+EP}AuNP_{Lu\&Lap}$ incubated with 100 mM H_2O_2 solution and NQO1/NADH enzymes in Tris-HCl buffer for different time.

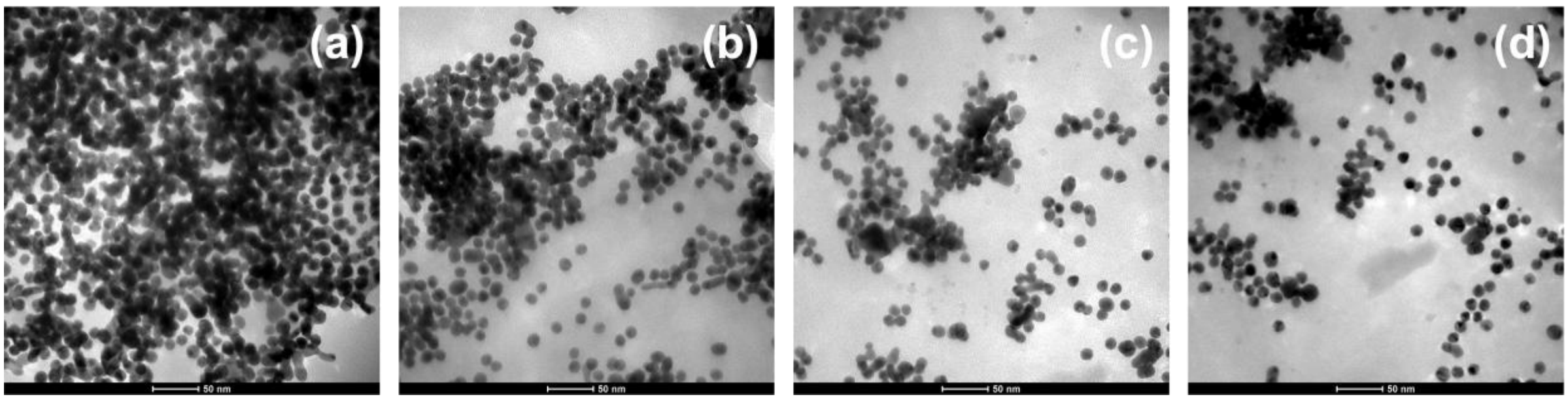

Figure S19. *In vitro* aggregation experiments under different TME conditions. TEM images of $_{T+EP}AuNP_{Lu\&Lap}$ under a) pH 7.4 with low GSH (10 μM); b) pH 6.5 with low GSH (10 μM); c) pH 7.4 with high GSH (10 mM); d) pH 6.5 with high GSH (10 mM) incubated with 100 mM H_2O_2 solution and NQO1/NADH enzymes in Tris-HCl buffer for 30 min.

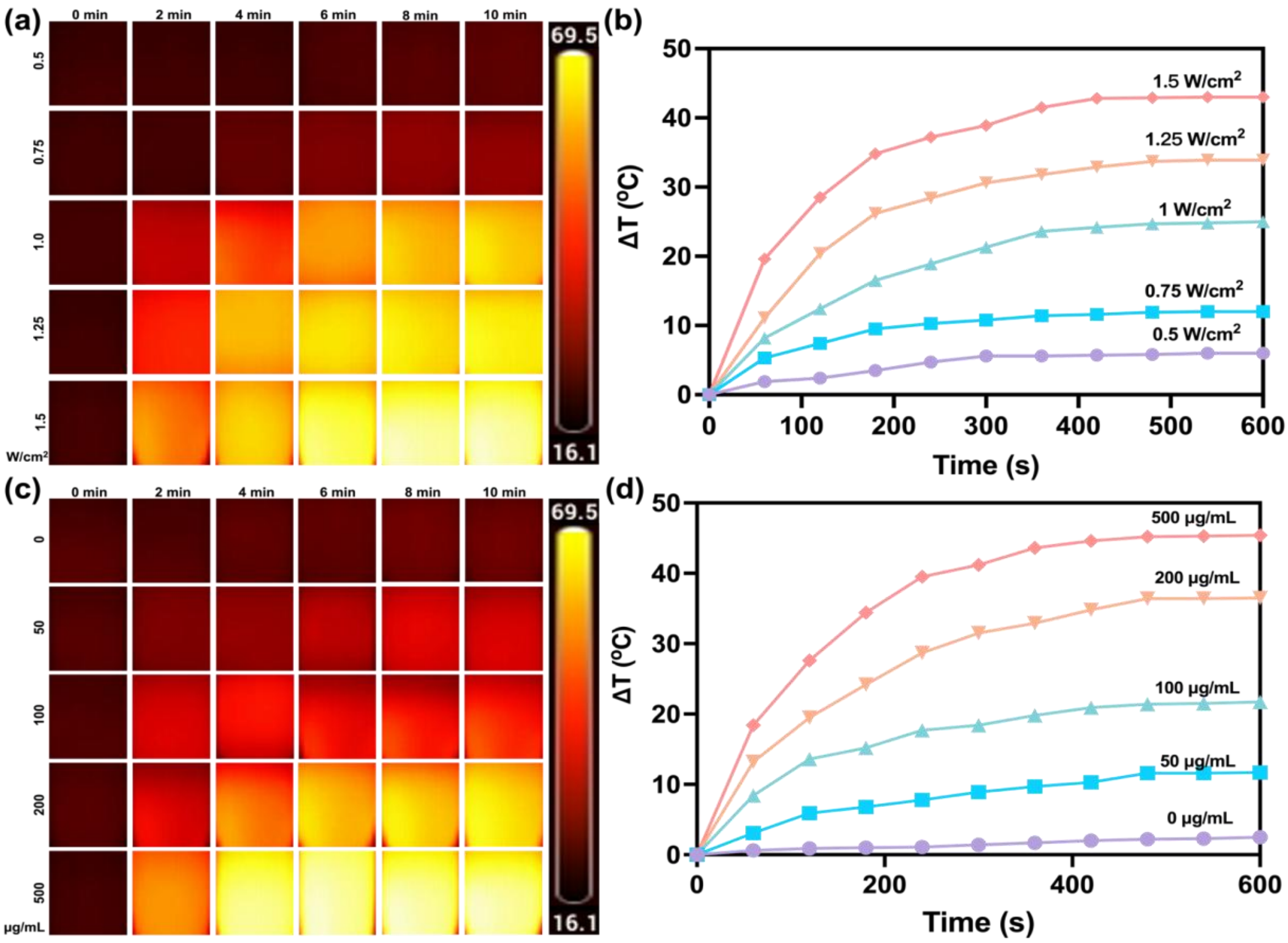

Figure S20. Photothermal performance evaluated by temperature recording of $_{T+EP}$AuNP$_{Lu\&Lap}$. a) Power intensity-dependent photothermal effect of and b) corresponding temperature change of $_{T+EP}$AuNP$_{Lu\&Lap}$ aqueous solution applying different 808 nm laser power intensities (200 μg mL^{-1}). c) Concentration-dependent photothermal effect and d) corresponding temperature change of different concentrations of $_{T+EP}$AuNP$_{Lu\&Lap}$ aqueous solution (1.25 W cm^{-2}).

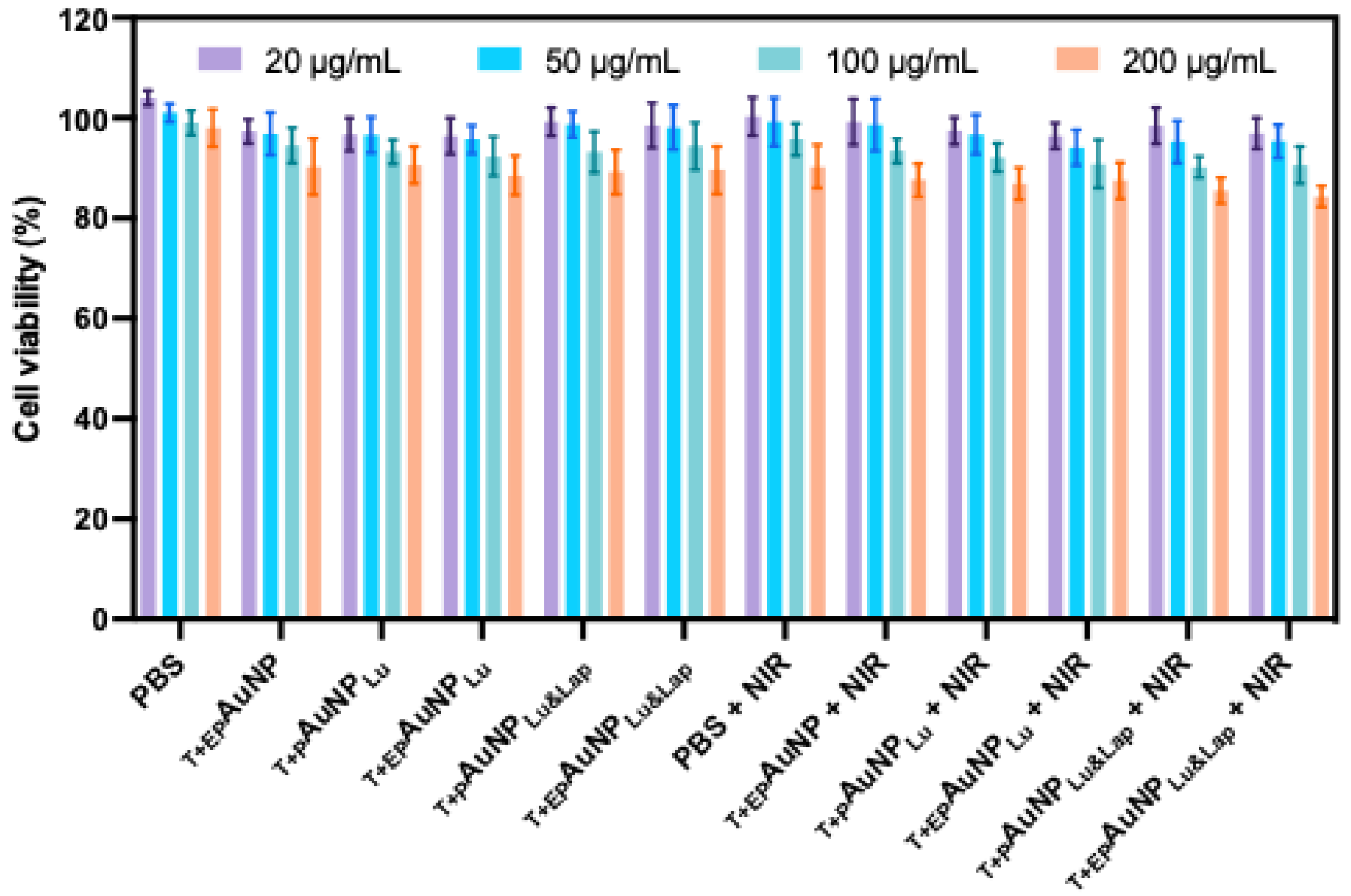

Figure S21. Cell viabilities of $_{T+EP}AuNP_{Lu\&Lap}$ and its counterparts at different concentrations for L929 normal cells with or without 808 nm laser irradiation. The results indicated excellent biocompatibity and negligible cytotoxicity toward non-cancerous normal cells. Quantitative data are presented as mean ± SD (n=3).

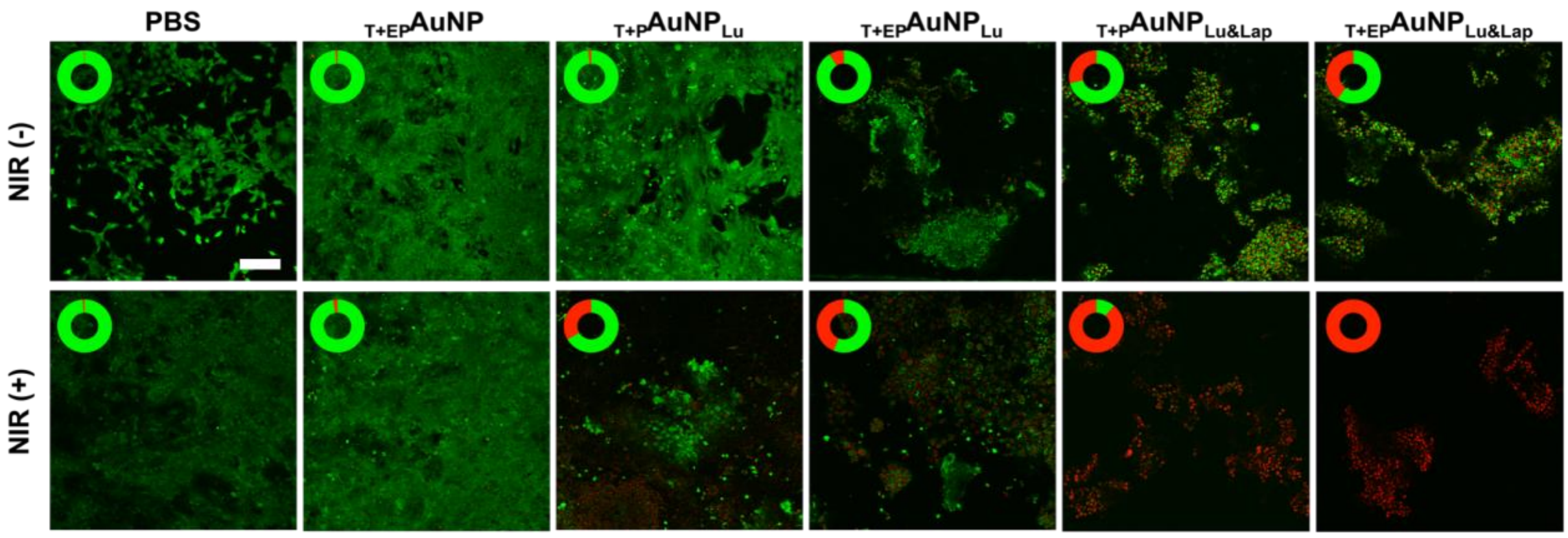

Figure S22. Live-dead staining images of murine 4T1 cells after therapeutic treatment. Scale bar: 100 μm.

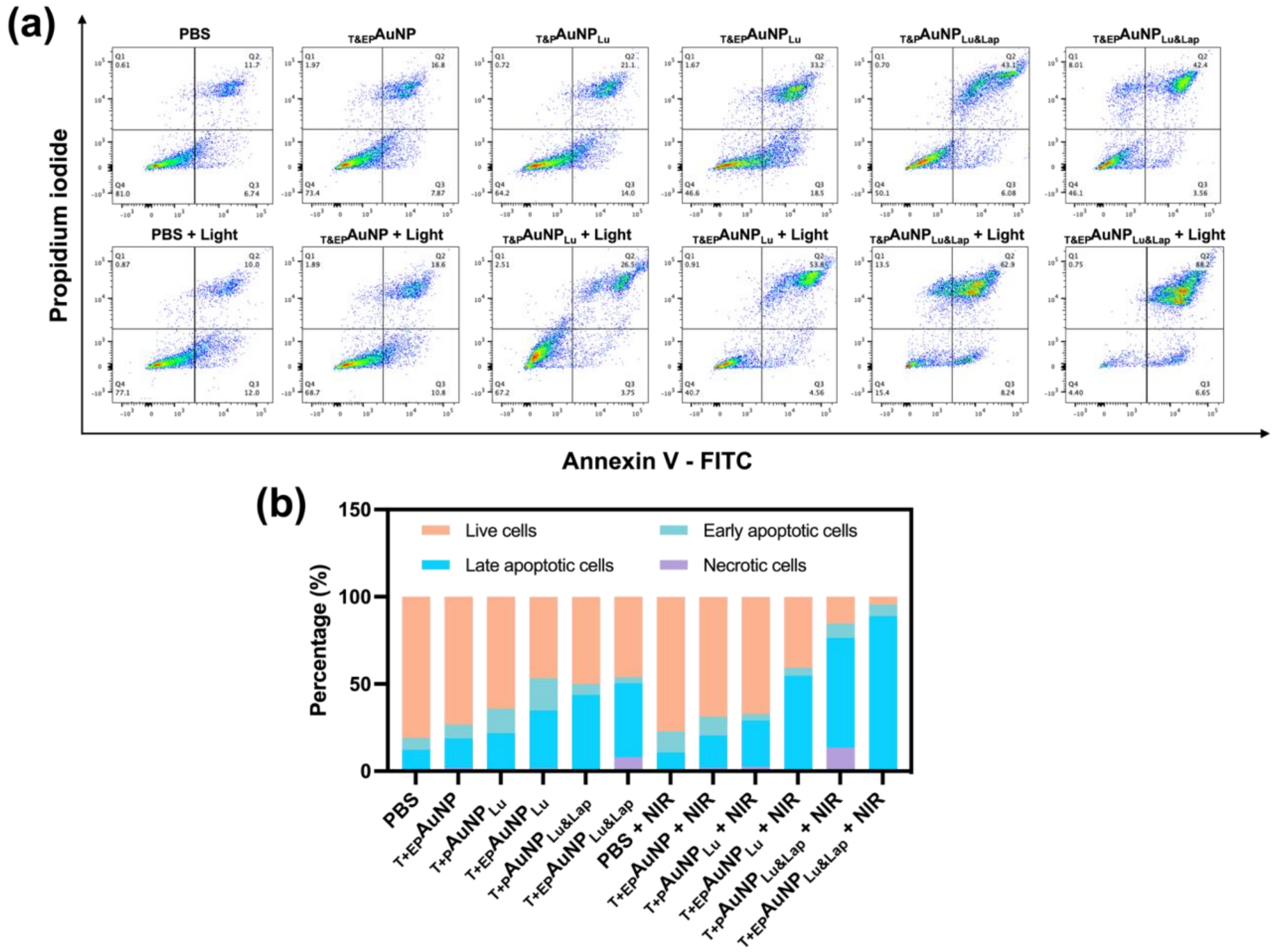

Figure S23. Evaluation of cytotoxicity of $_{T+EP}AuNP_{Lu\&Lap}$ and its counterparts toward tumor cells via flow cytometry analysis. a) Apoptosis rates of 4T1 cells receiving different treatments, determined by flow cytometry after 24 h of co-incubation (100 μg/mL, 808 nm laser: 1.25 W/cm^2, t = 10 min). b) Corresponding quantitative analysis of cell apoptosis percentages.

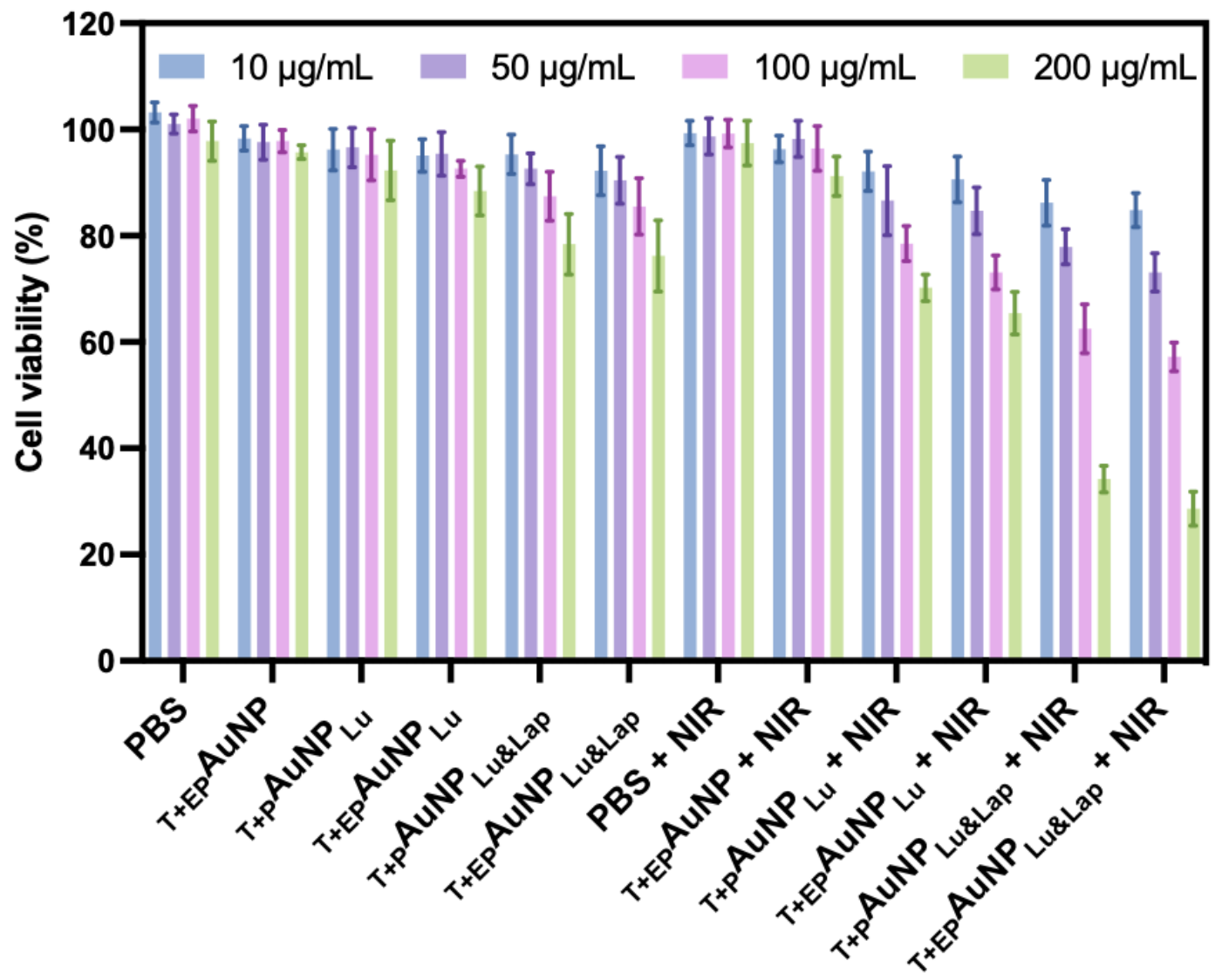

Figure S24. Cell viability of TEMPO-pretreated 4T1 cells after incubation with $_{T+EP}AuNP_{Lu\&Lap}$ and its counterparts with or without 808 nm NIR irradiation for verification of ROS generation and cytotoxicity. Quantitative data are presented as mean ± SD (n=3).

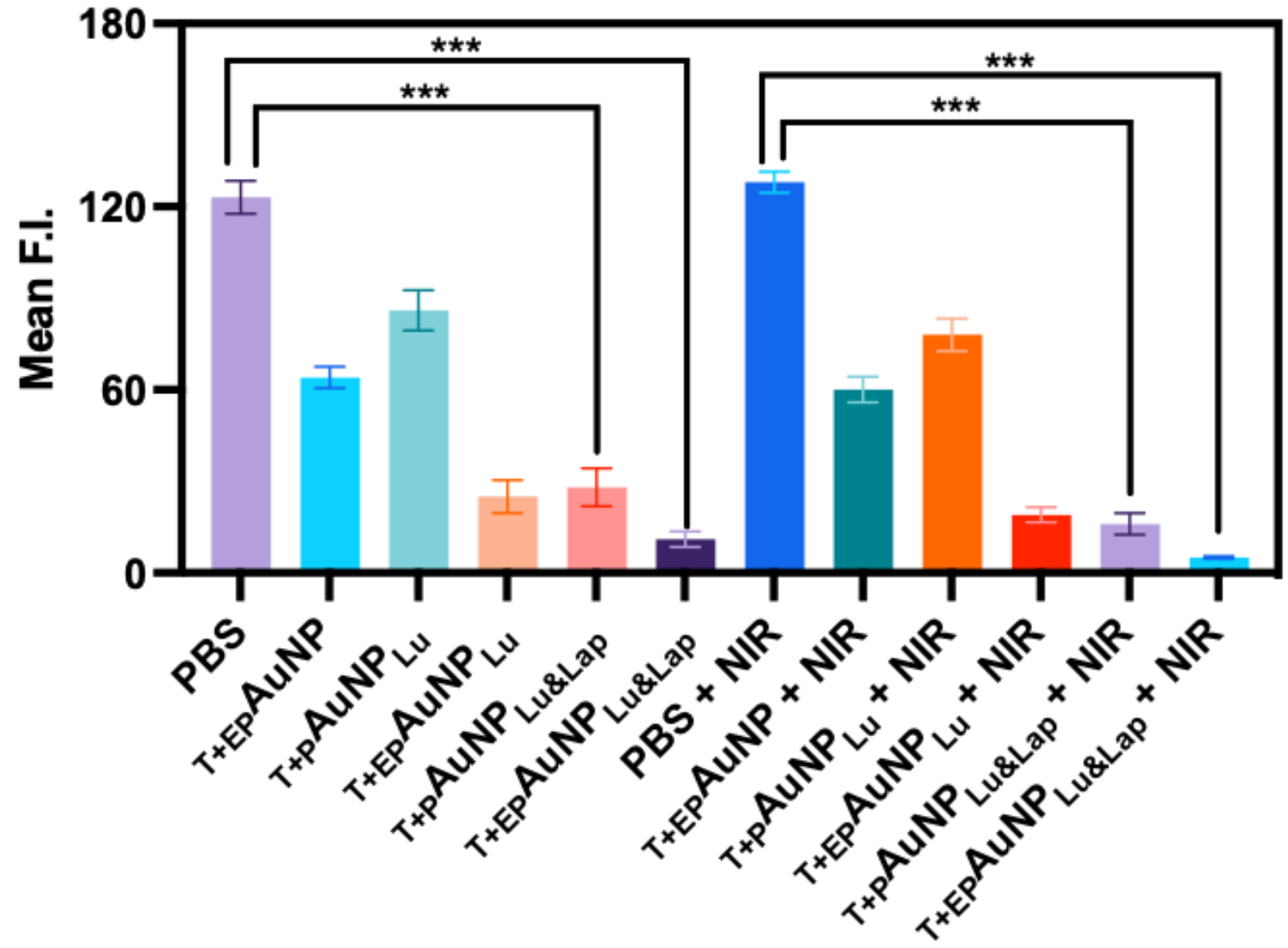

Figure S25. Quantitative analysis of mean fluorescence intensity of the CLSM images of ThiolTracker Violet fluorescence. Quantitative data are presented as mean ± SD (n=3).

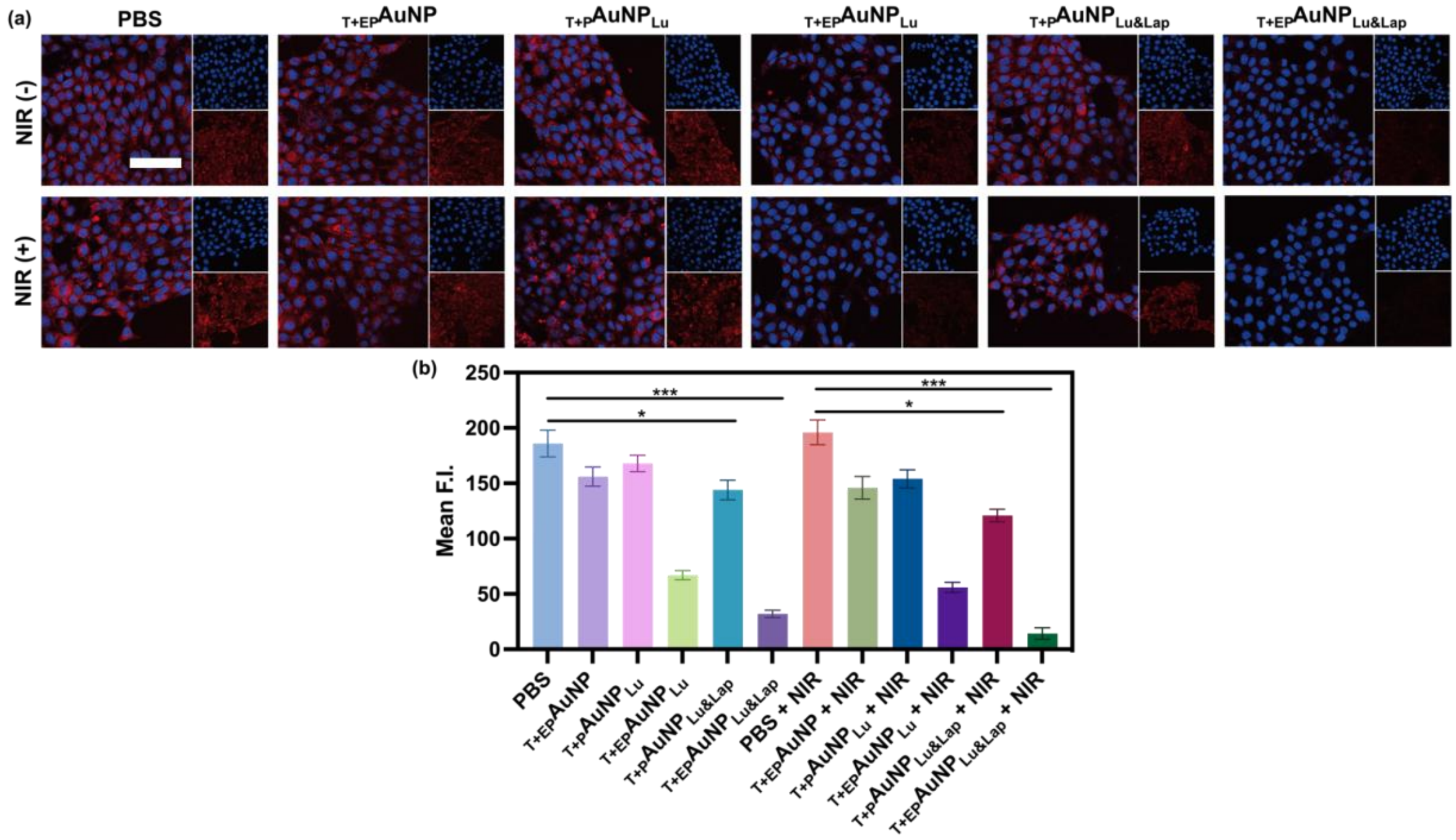

Figure S26. Confirmation of O_2 releasing ability by a) CLSM images leveraging O_2-sensitive Ru(dpp)$_3$Cl$_2$ dye-stained 4T1 cells and b) corresponding quantitative analysis. Quantitative data are presented as mean ± SD (n=3). Scale bar: 50 μm.

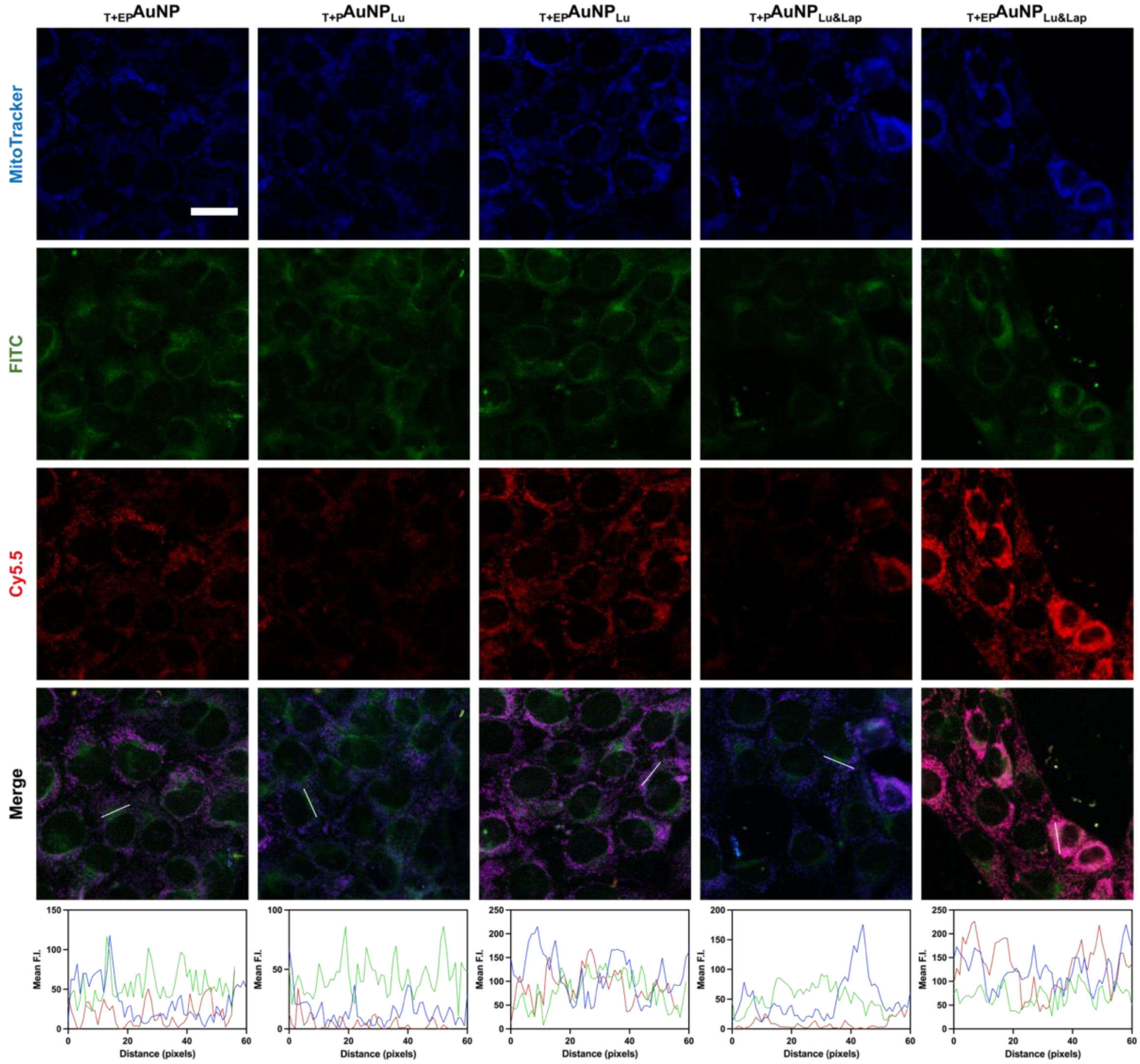

Figure S27. Verification of mitochondria-targeting ability of $_{T+EP}$AuNP$_{Lu\&Lap}$ and its counterparts via CLSM images using commercially-available MitoTracker-dyed 4T1 cells, where $_{T}$AuNP$_{Lu\&Lap}$ was stained by FITC and $_{EP}$AuNP$_{Lu\&Lap}$ (and its counterparts) by Cy5.5. Overlapped fluorescence with MitoTracker indicated mitochondria-targetability, while strong inclination to aggregation was exhibited by overlapped fluorescence of FITC and Cy5.5 dyes, valdated by co-localization analysis. Scale bar: 20 μm.

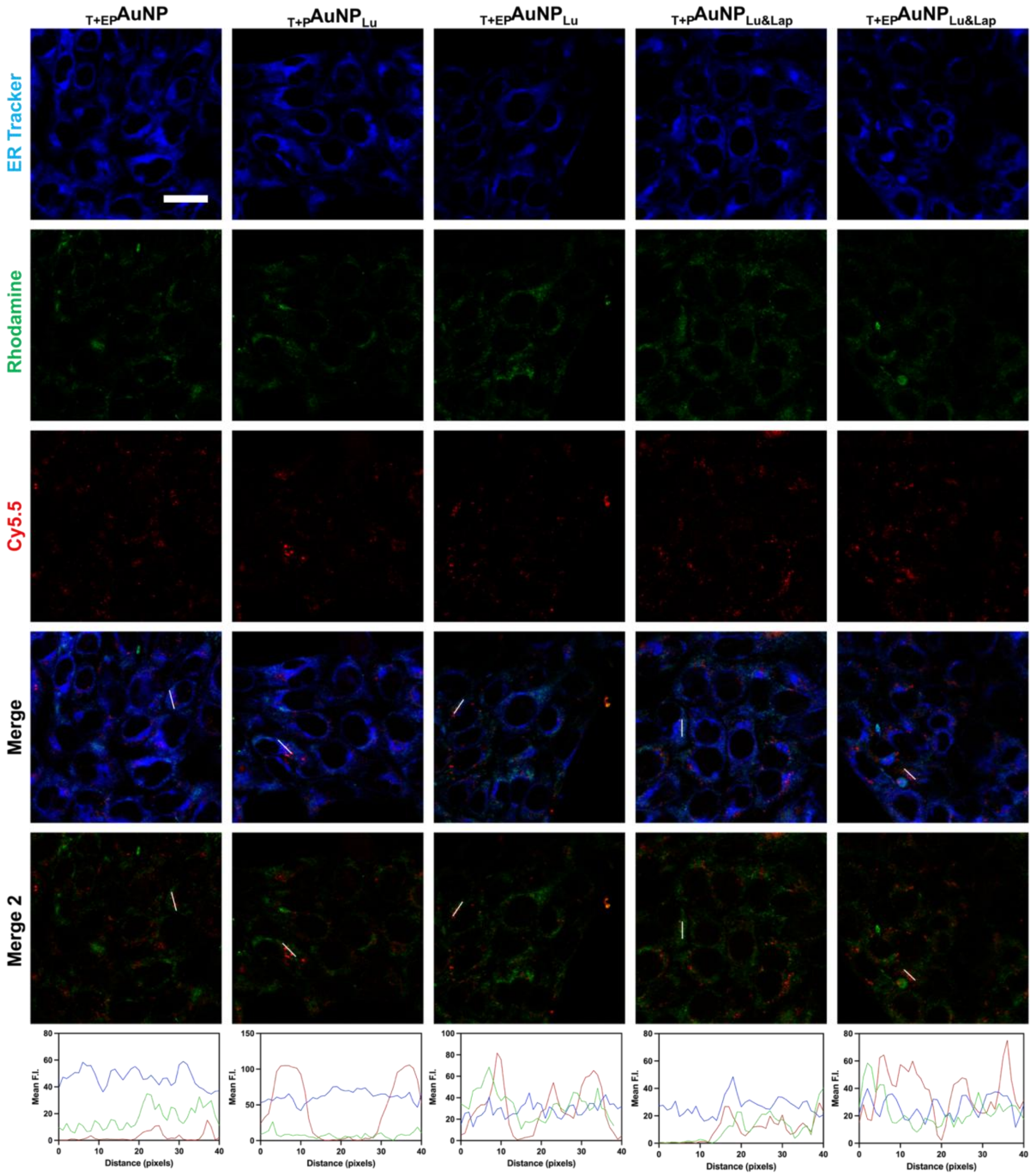

Figure S28. Substantiation of ER-locating ability of $_{T+EP}AuNP_{Lu\&Lap}$ and its counterparts via CLSM images using ER Tracker-dyed 4T1 cells, where $_{T}AuNP_{Lu\&Lap}$ was stained by Rhodamine and $_{EP}AuNP_{Lu\&Lap}$ (and its counterparts) by Cy5.5. Overlapped fluorescence with ER Tracker indicated ER-locating capability (Merge), while proclination to aggregation was manifested by overlapped fluorescence of FITC and Cy5.5 dyes (Merge 2), verified by co-localization analysis. Scale bar: 20 μm.

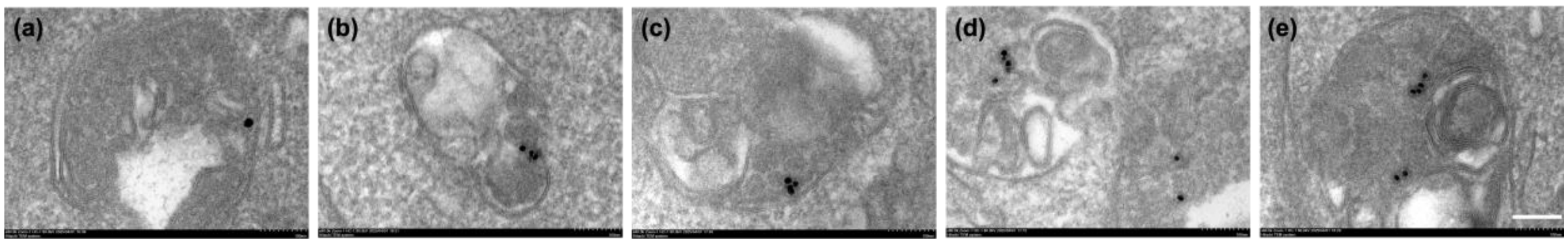

Figure S29. Bio-TEM images of L929 cell lines of a) $_{T+EP}$AuNP, b) $_{T+P}$AuNP$_{Lu,}$ c) $_{T+EP}$AuNP$_{Lu}$, d) $_{T+P}$AuNP$_{Lu\&Lap}$ and e) $_{T+EP}$AuNP$_{Lu\&Lap}$. Scale bar: 100 nm.

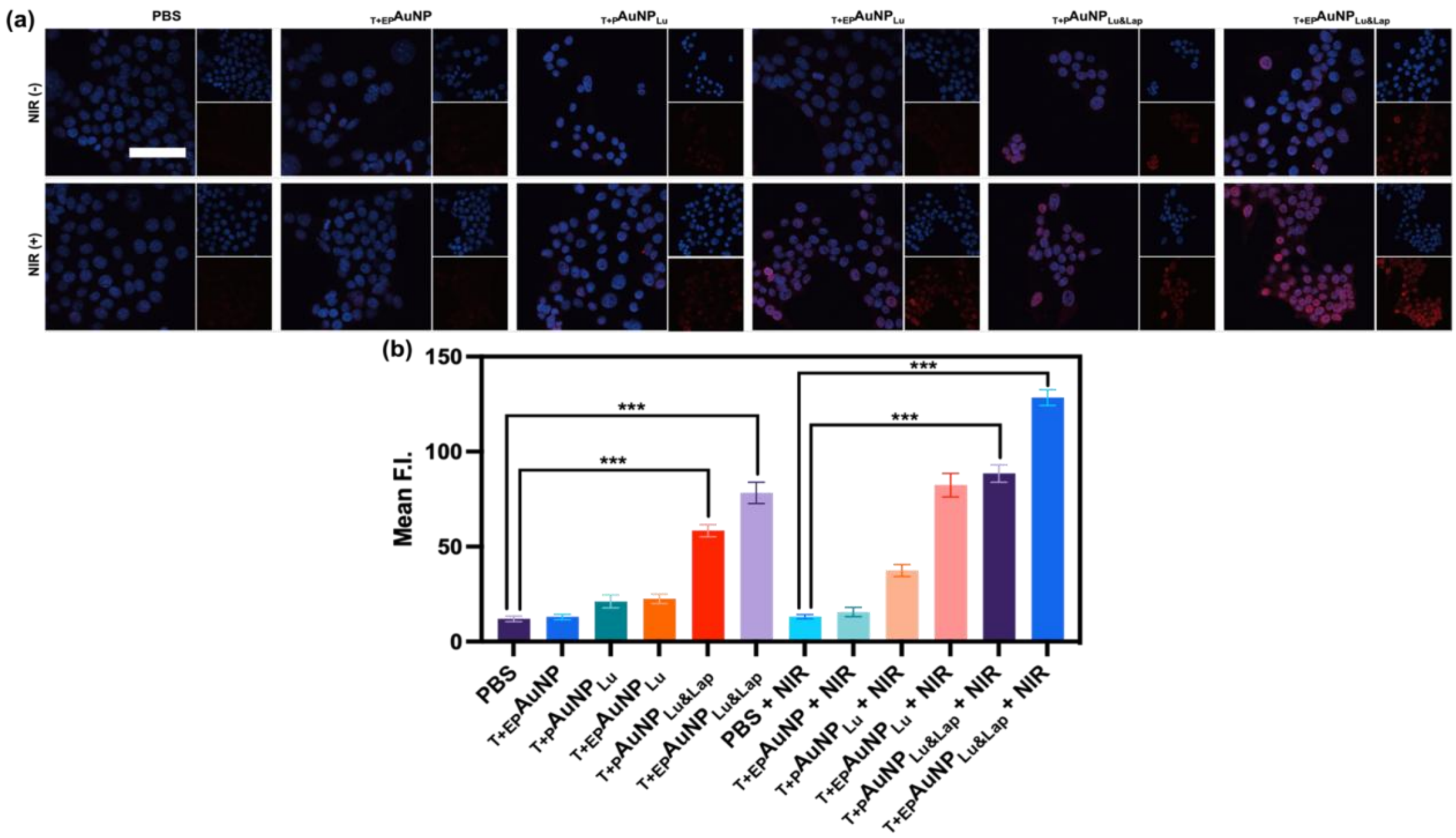

Figure S30. Verification of intracellular DNA release. a) γ-H2AX staining CLSM images for double-stranded breaks in DNA b) Corresponding quantitative analysis of mean fluorescence intensity of the CLSM images of γ-H2AX fluorescence. Data are presented as mean ± SD (n=3). Scale bar: 50 μm.

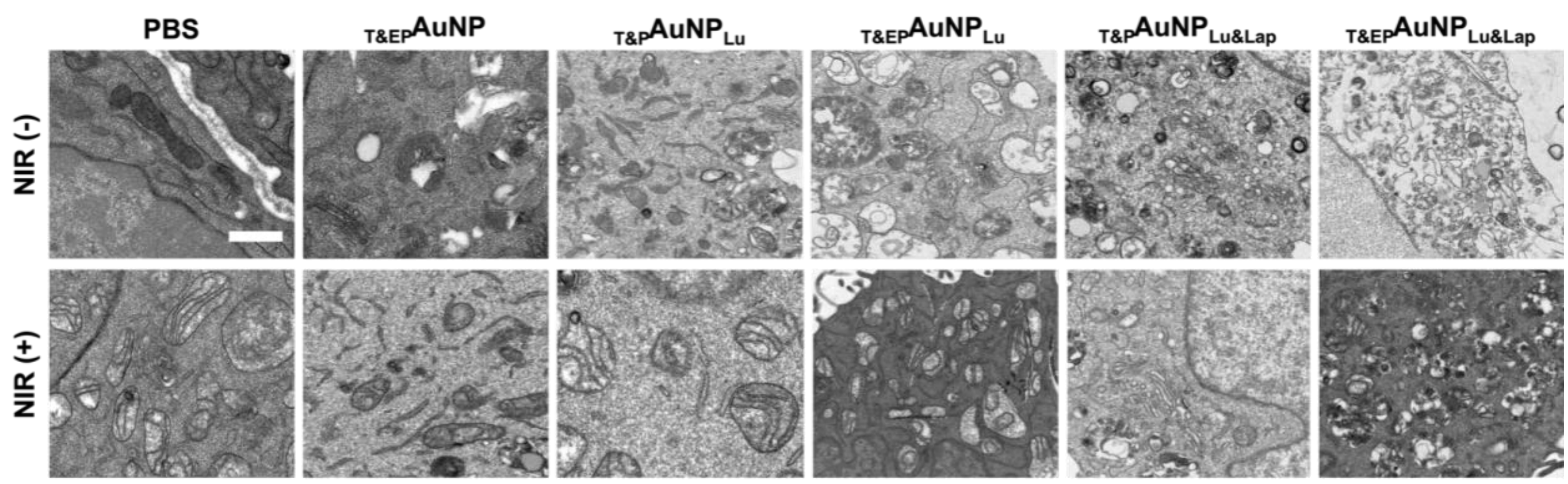

Figure S31. Bio-TEM images of 4T1 cells receiving different combinations of treatment to illustrate the morphological changes of mitochondria and ER. Scale bar: 100 nm.

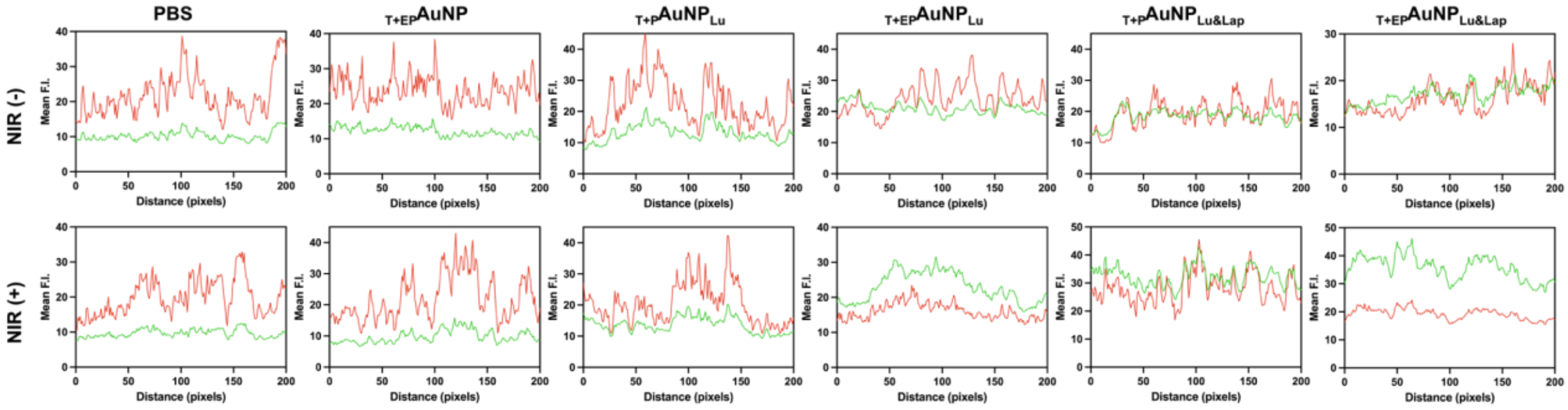

Figure S32. Corresponding quantitative analysis and comparisons of mean fluorescence intensity of the CLSM images of JC-1 fluorescence.

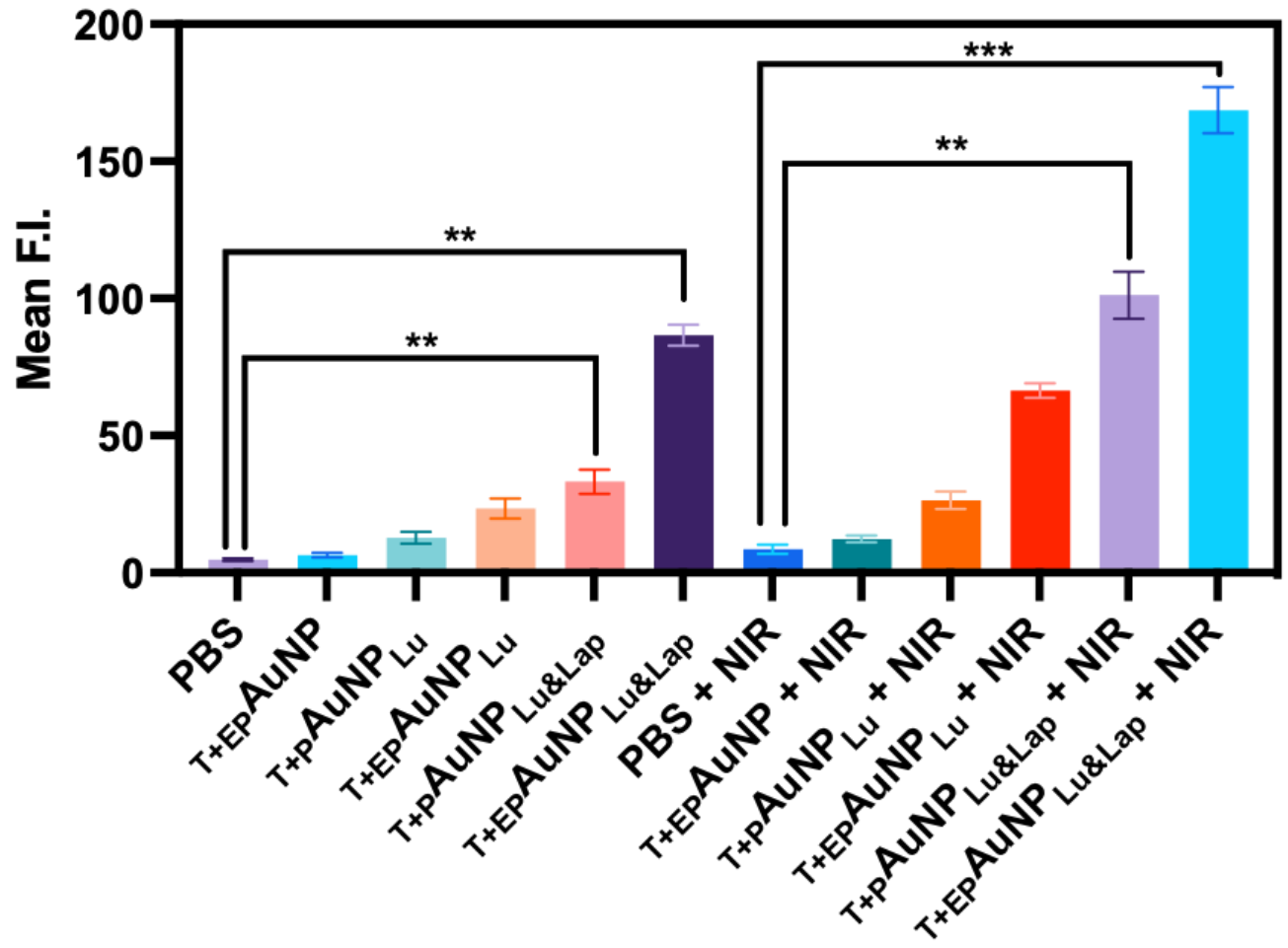

Figure S33. Corresponding quantitative analysis of mean fluorescence intensity of the CLSM images of p-STING fluorescence. Data are presented as mean ± SD (n=3).

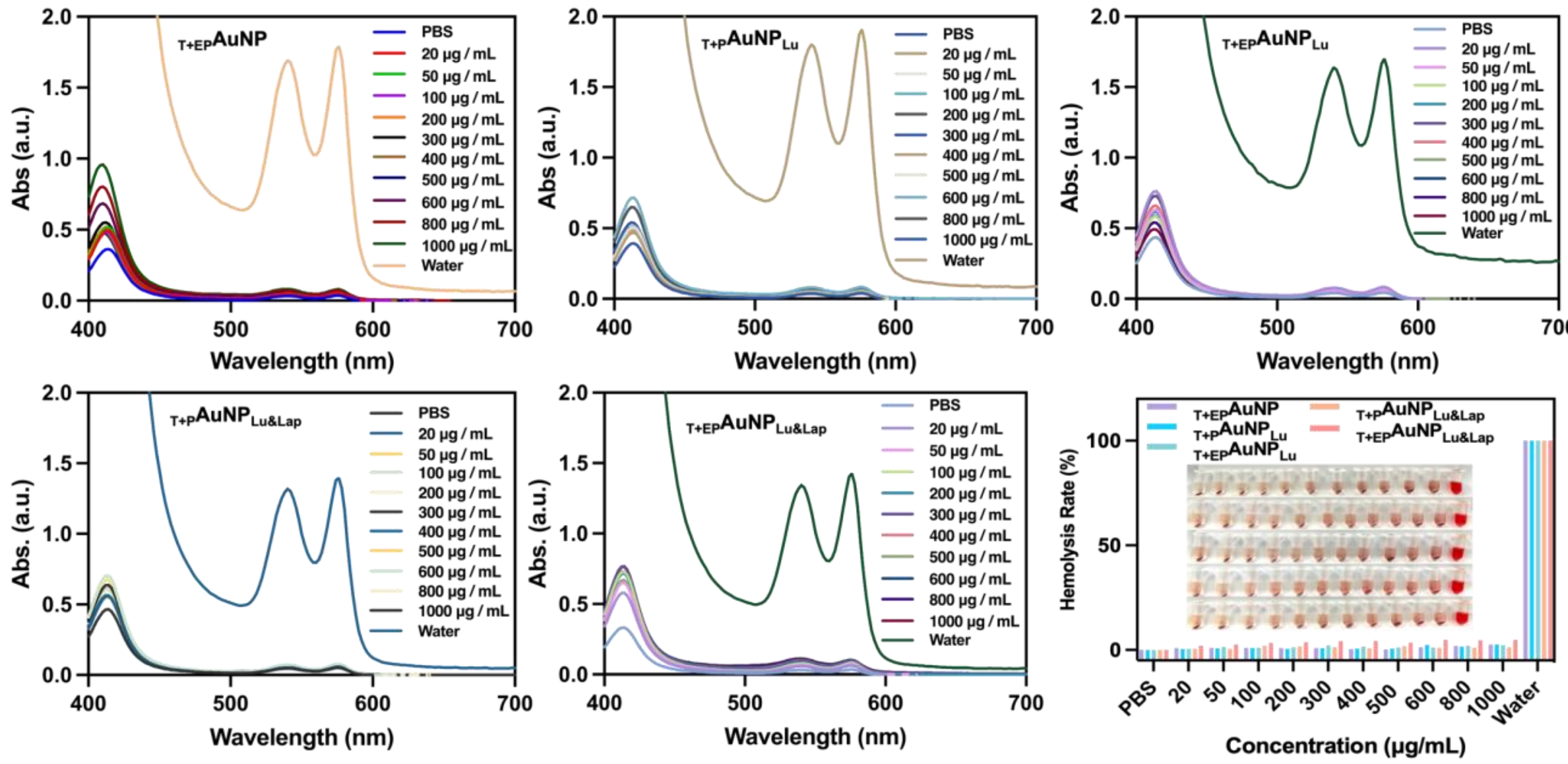

Figure S34. Hemolysis analysis by UV-Vis absorption. Absorption profiles of different concentrations of T+EPAuNP, T+PAuNP$_{Lu}$, T+EPAuNP$_{Lu}$, T+PAuNP$_{Lu\&Lap}$ and T+EPAuNP$_{Lu\&Lap}$.

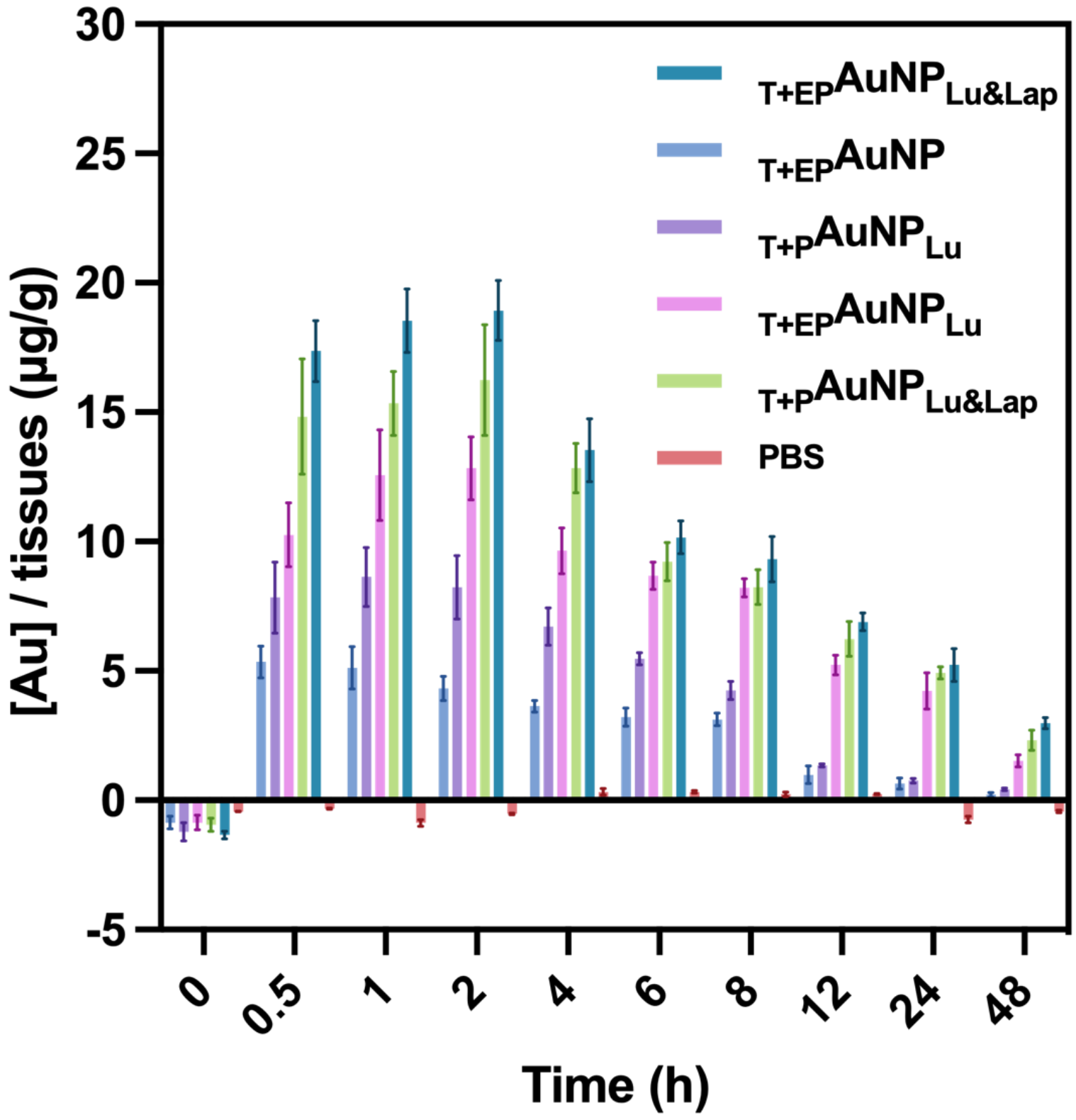

Figure S35. *Ex vivo* ICP-based time-dependent Au content quantification at the tumor site of $_{T+EP}AuNP_{Lu\&Lap}$ and its counterparts. (0, 0.5, 1, 2, 4, 6, 8, 12, 24, 48 h after injection, n=3)

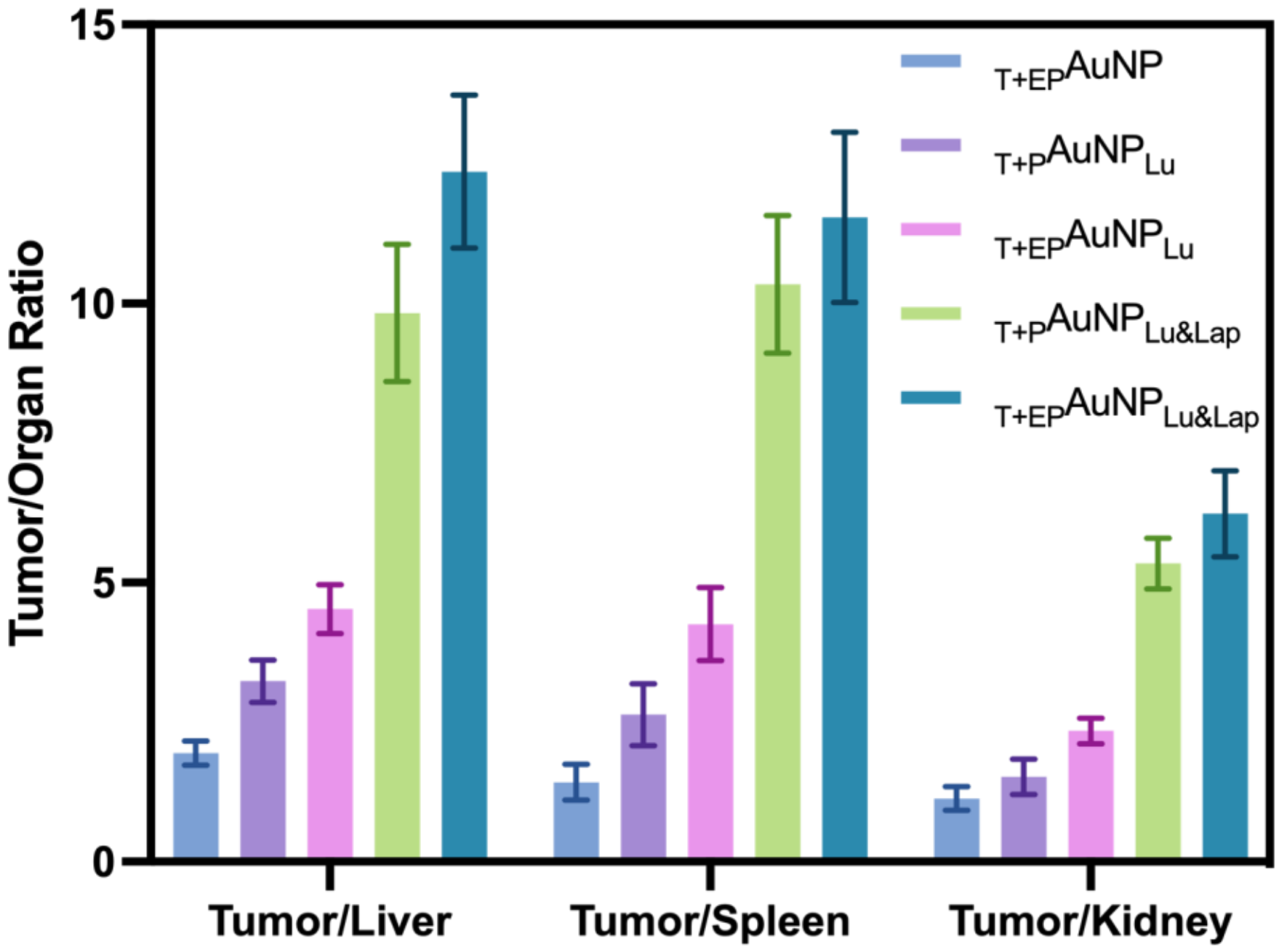

Figure S36. *Ex vivo* ICP-based tumor/organ ratio according to Au content of $_{T+EP}AuNP_{Lu\&Lap}$ and its counterparts 1 h post-injection. Data are presented as mean ± SD (n=3).

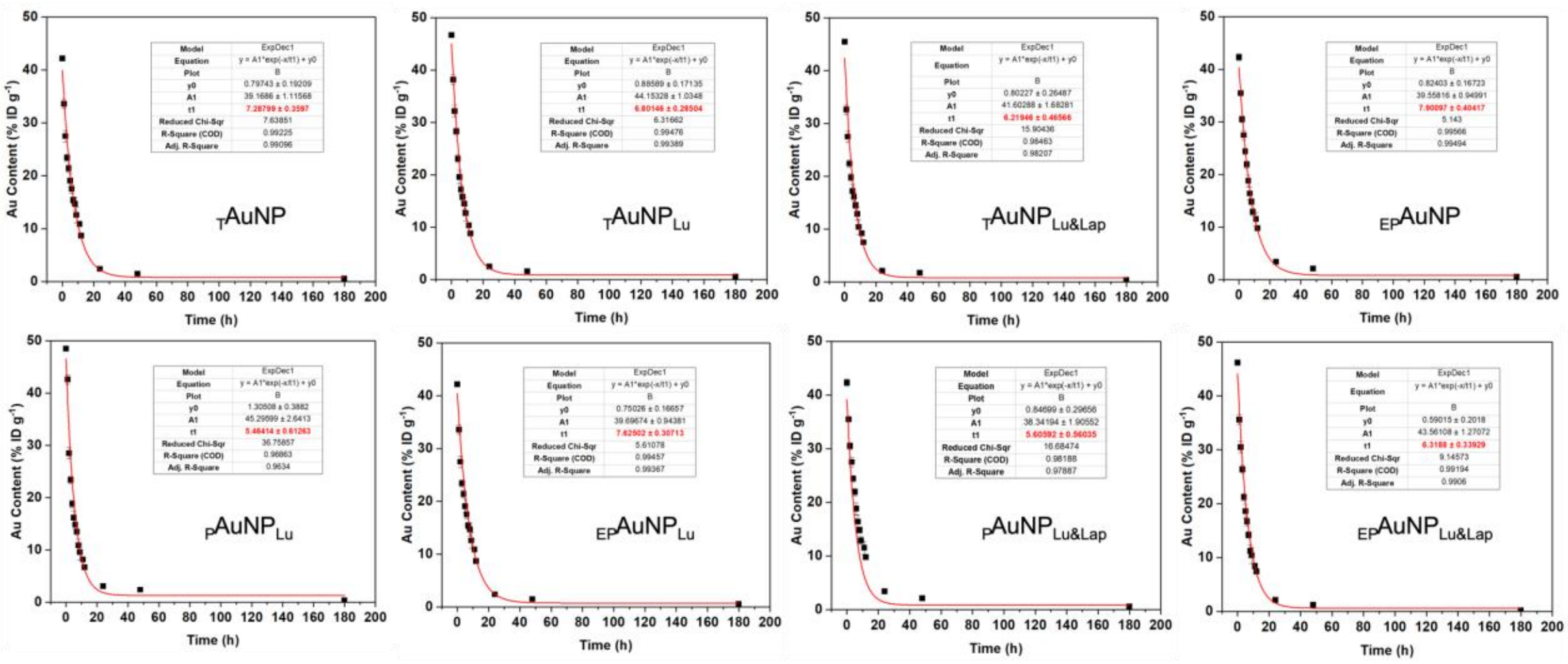

Figure S37. Blood pharmacokinetic profiles of $_{T}AuNP$, $_{T}AuNP_{Lu}$, $_{T}AuNP_{Lu\&Lap}$, $_{EP}AuNP$, $_{P}AuNP_{Lu}$, $_{EP}AuNP_{Lu}$, $_{P}AuNP_{Lu\&Lap}$ and $_{EP}AuNP_{Lu\&Lap}$, which were fitted into a single compartment model. With the main pharmacokinetic parameters listed, it is revealed that $t_{1/2}$ = 7.29 h for $_{T}AuNP$, $t_{1/2}$ = 6.80 h for $_{T}AuNP_{Lu}$, $t_{1/2}$ = 6.22 h for $_{T}AuNP_{Lu\&Lap}$, $t_{1/2}$ = 7.90 h for $_{EP}AuNP$, $t_{1/2}$ = 5.46 h for $_{P}AuNP_{Lu}$, $t_{1/2}$ = 7.62 h for $_{EP}AuNP_{Lu}$, $t_{1/2}$ = 5.61 h for $_{P}AuNP_{Lu\&Lap}$, and $t_{1/2}$ = 6.32 h for $_{EP}AuNP_{Lu\&Lap}$ (n=3).

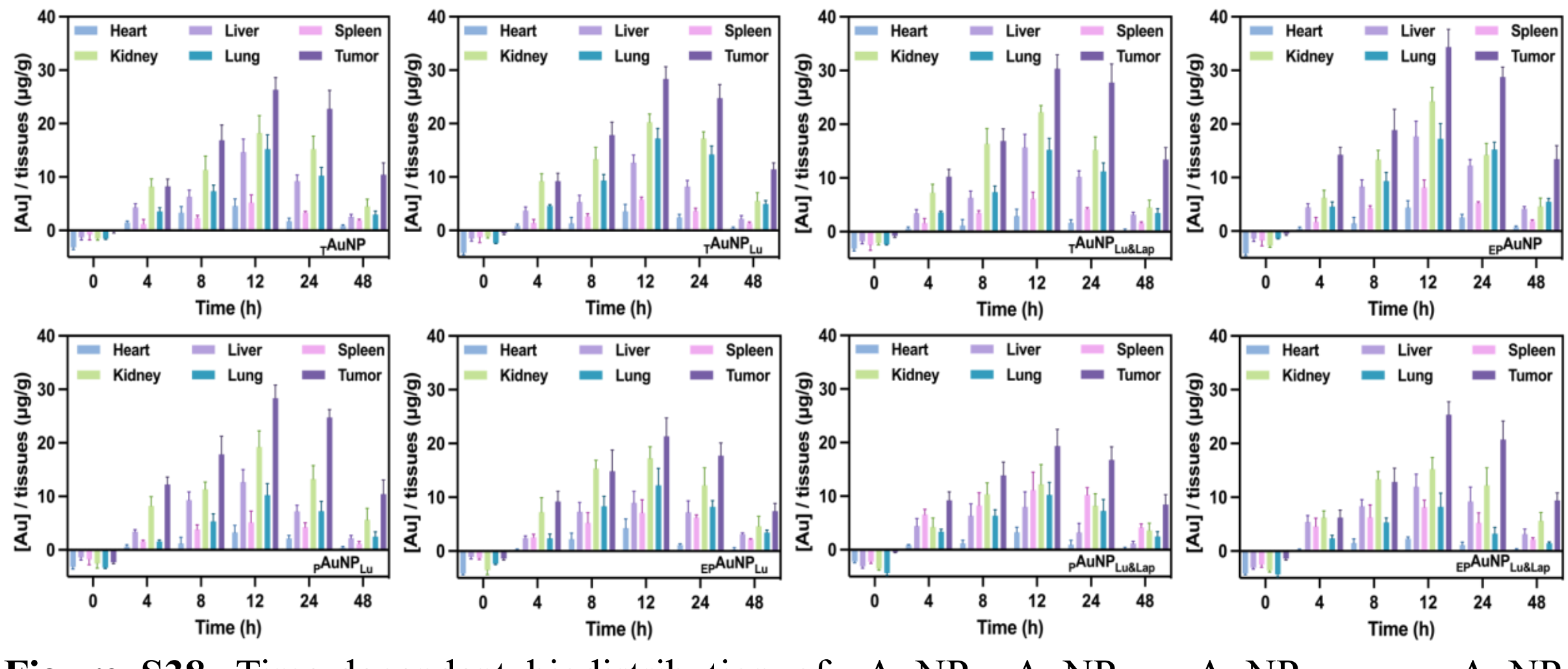

Figure S38. Time-dependent biodistribution of $_{T}AuNP$, $_{T}AuNP_{Lu}$, $_{T}AuNP_{Lu\&Lap}$, $_{EP}AuNP$, $_{P}AuNP_{Lu}$, $_{EP}AuNP_{Lu}$, $_{P}AuNP_{Lu\&Lap}$ and $_{EP}AuNP_{Lu\&Lap}$ in the major organs and tumor tissues at different time points by Au content (0, 4, 8, 12, 24, 48 h after injection, n=3).

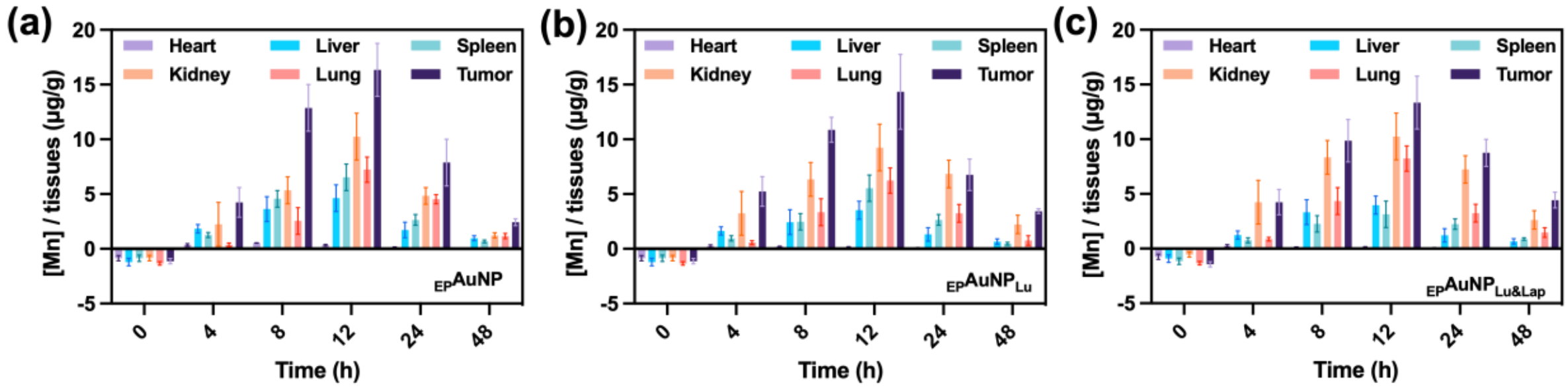

Figure S39. Time-dependent biodistribution of a) $_{EP}$AuNP, b) $_{EP}$AuNP$_{Lu}$, and c) $_{EP}$AuNP$_{Lu\&Lap}$ in the major organs and tumor tissues at different time points by Mn content (0, 4, 8, 12, 24, 48 h after injection, n=3).

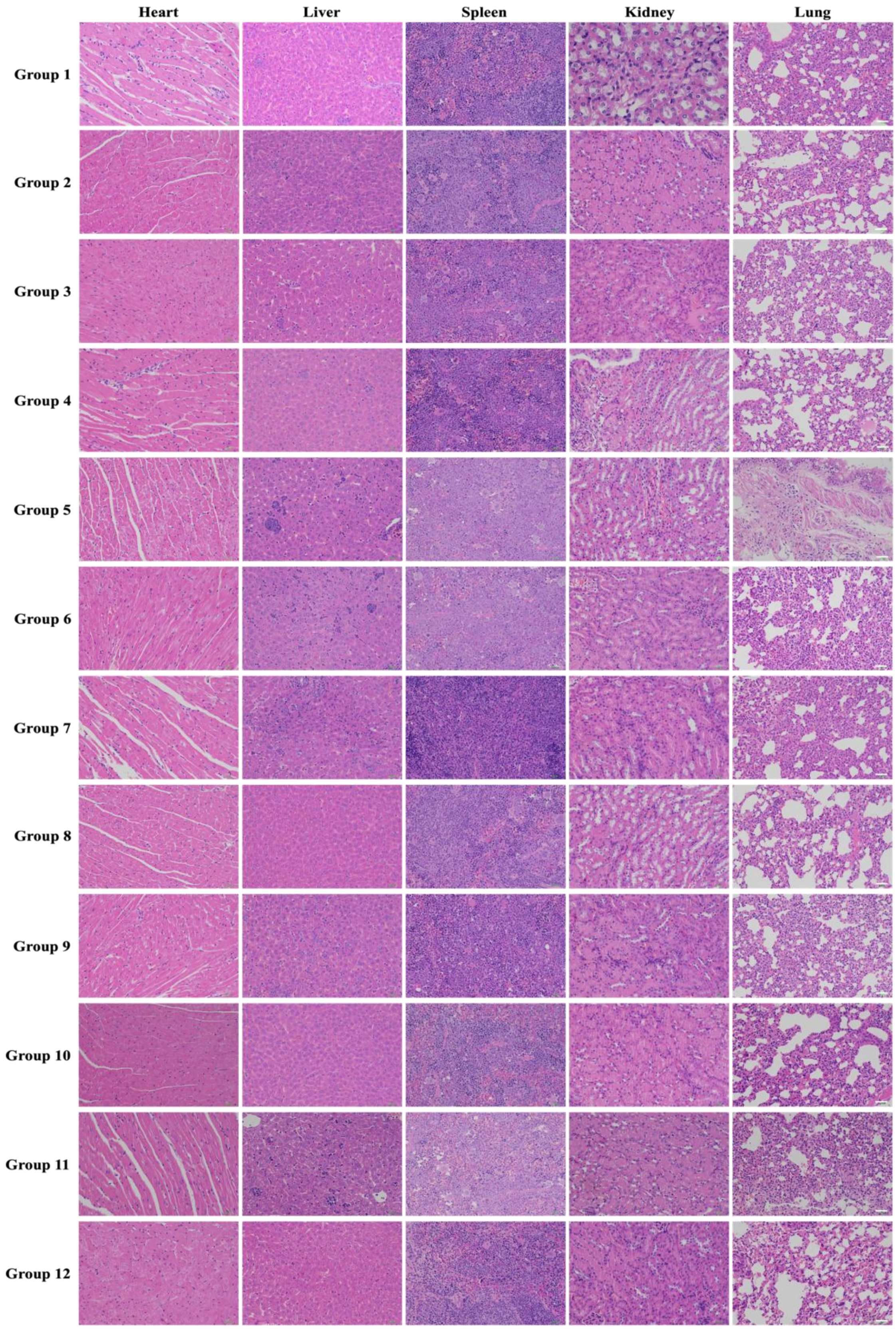

Figure S40. Representative H&E staining images of major organs dissected from different groups after *in vivo* synergistic therapeutic experiments. The results suggest no visible sabotage to the selected major organs of the mice receiving corresponding treatment. Scale bar: 50 μm.

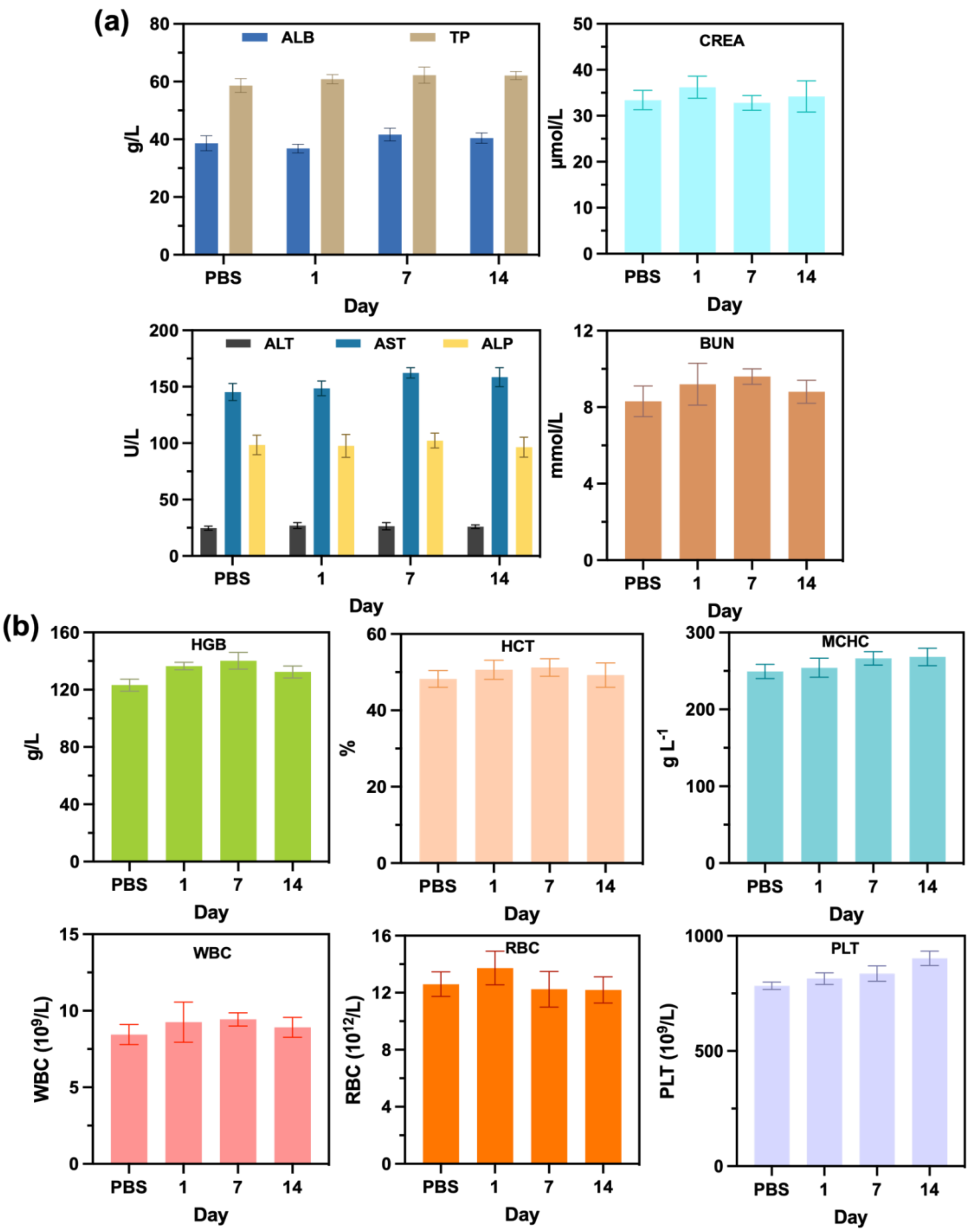

Figure S41. Hematological analysis for biosafety appraisal. a) Blood biochemistry and b) hematology data after treated with $_{T+EP}$AuNP$_{Lu\&Lap}$ (20 mg kg^{-1}) at different time points (at 0, 1, 7, 14 days, n=3). ALB and TP indicate blood albumin or protein, advising liver or kidney functions. CREA and BUN suggest kidney health, while ALT, AST and ALP inform liver health. All the indices of blood biochemistry and hematology within normal range indicated good health condition of mice receiving treatment. Quantitative data are presented as mean ± SD (n=3).

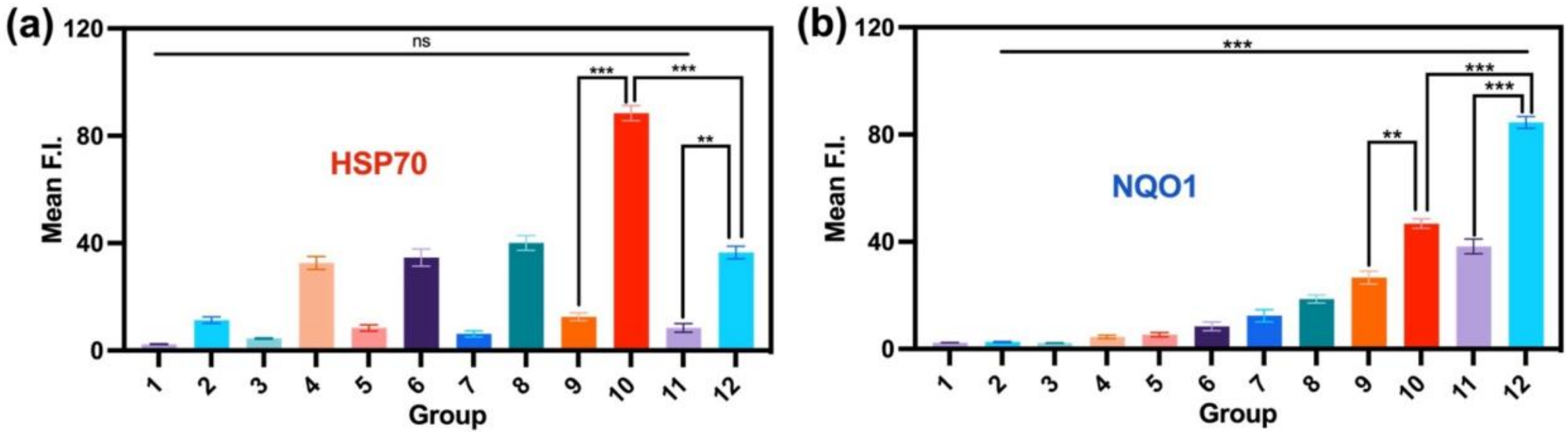

Figure S42. Corresponding quantitative analysis of mean fluorescence intensity of the CLSM images of HSP70 and NQO1 fluorescence in mice receiving different combinations of therapy. Quantitative data are presented as mean ± SD (n=3).

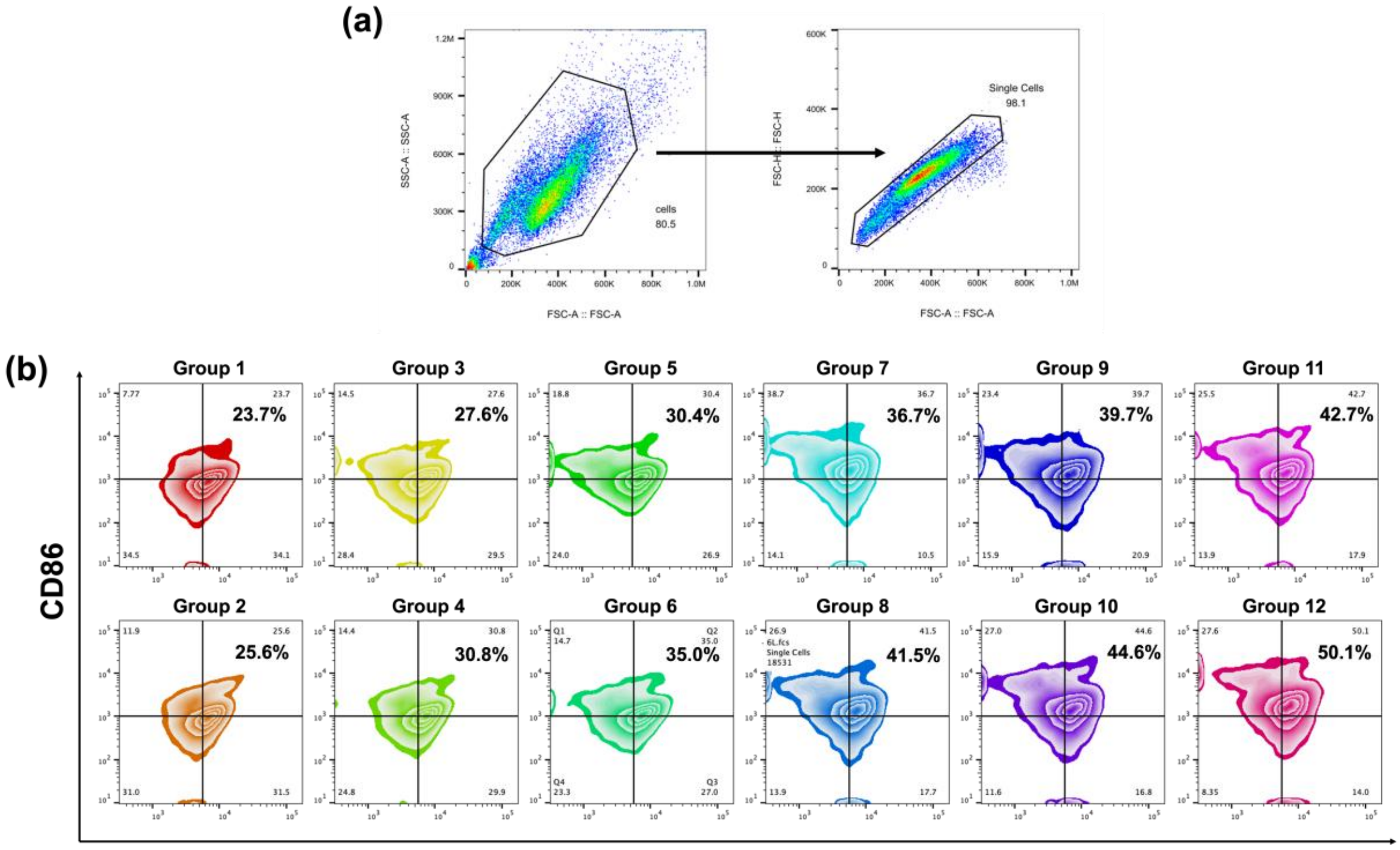

Figure S43. Assessment of DC maturation upon treatment. a) Gating strategy of DC for analysis. b) Populations of DCs in lymph nodes of mice receiving different regimens of treatment. Data are presented as mean ± SD (n=3). Statistical significance between every two groups was calculated via one-way ANOVA. *$p < 0.05$, **$p < 0.01$, and ***$p < 0.001$. 808 nm NIR irradiation was executed with an intensity of 1.25 W cm^{-2}.

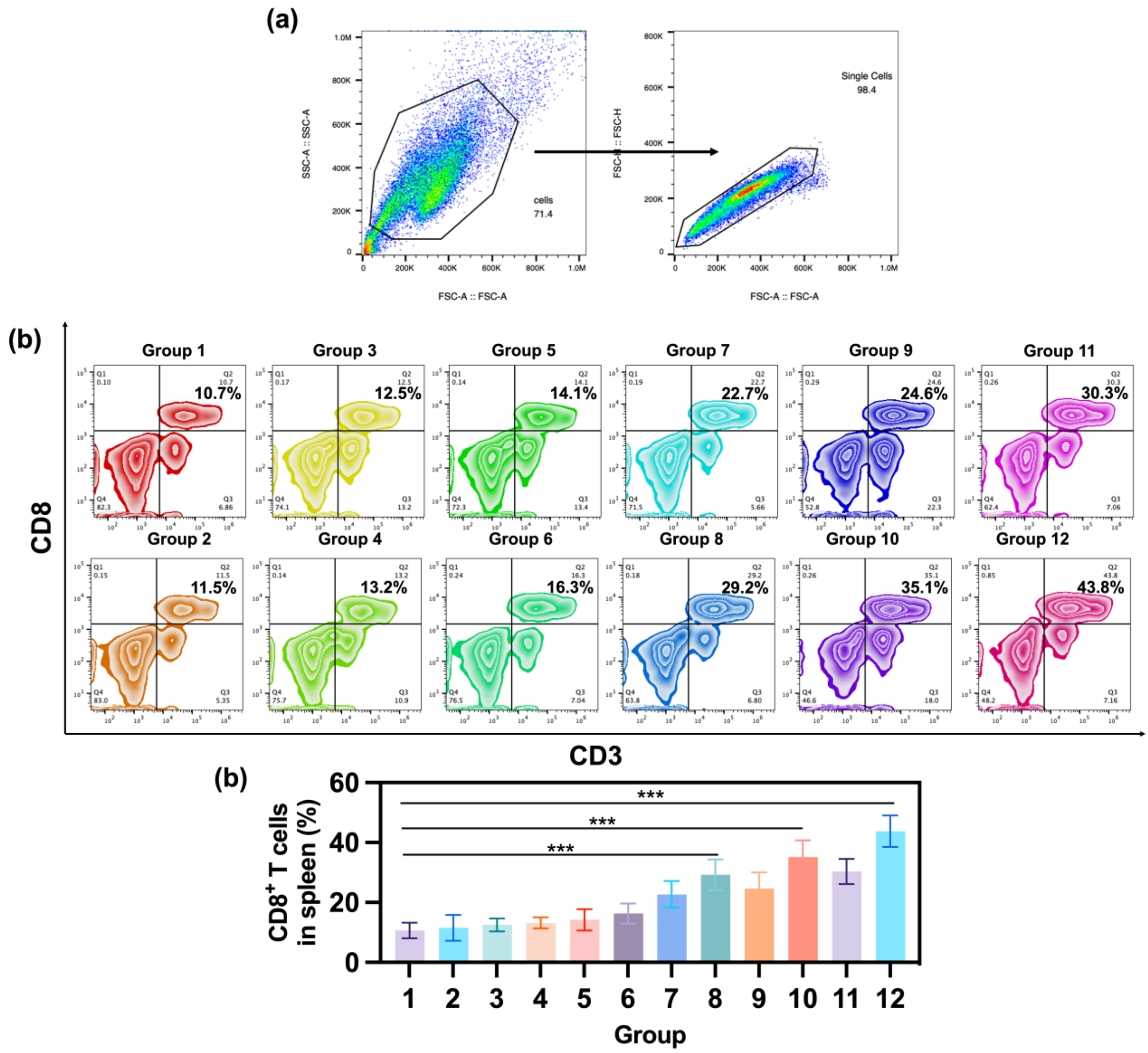

Figure S44. Evaluation of CD8$^+$ T cells activation upon treatment in splenocytes. a) Gating strategy of CD8+ T cells for analysis. b) The populations of CD8$^+$ T cells in spleen and c) corresponding quantitative analysis after different regimens of treatments. Data are presented as mean ± SD (n=3).

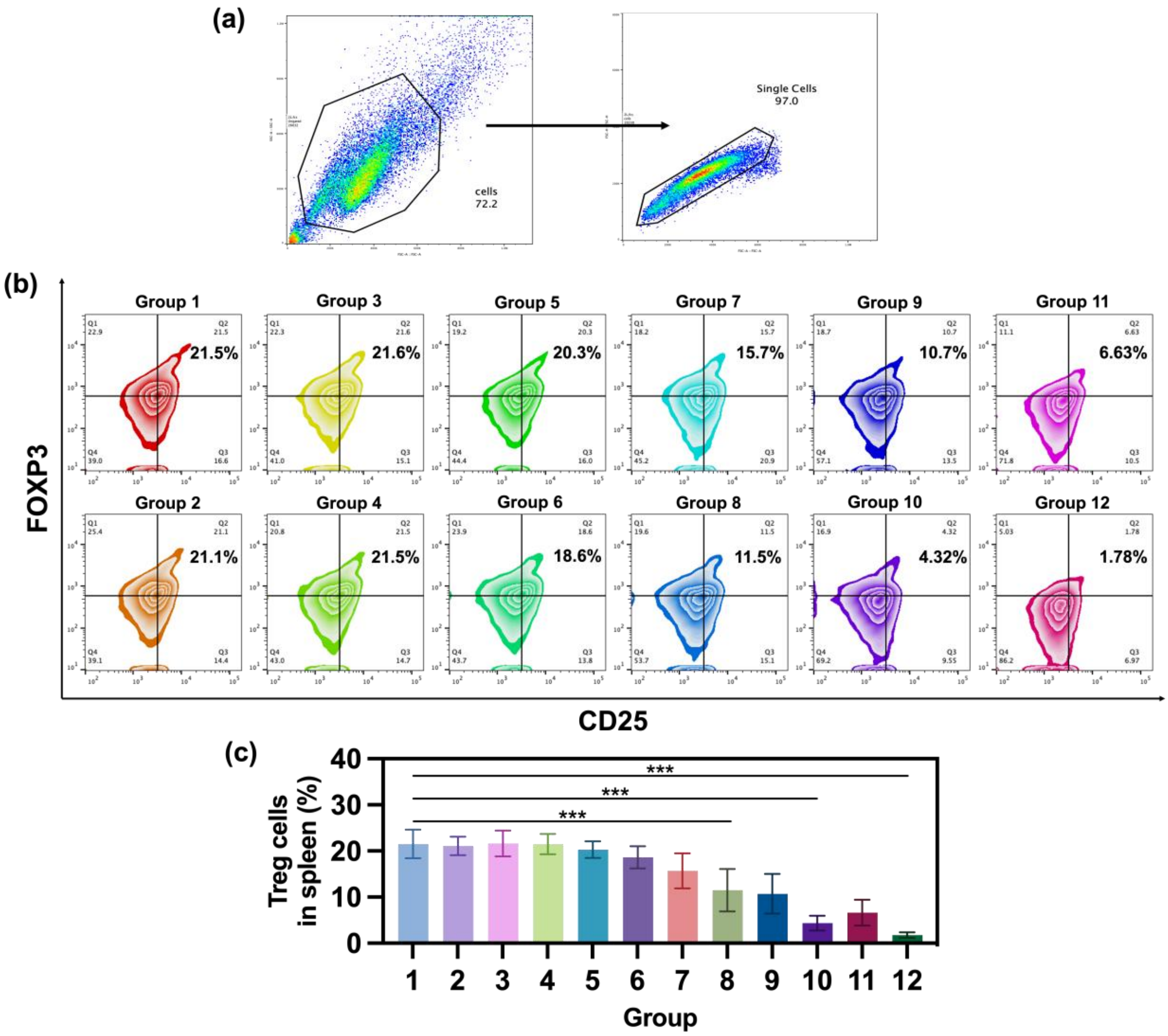

Figure S45. Evaluation of Treg cells suppression after treatment in splenocytes. a) Gating strategy of Treg cells for analysis. b) The populations of Treg cells in spleen and c) corresponding quantitative analysis after different regimens of treatments. Data are presented as mean ± SD (n=3).

Table S1. Electrochemical parameters of cyclic voltammetry

System	Anodic Peak Potential (E_{pa}) [V]	Anodic Peak Current (I_{pa}) [μA]	Cathodic Peak Potential (E_{pc}) [V]	Cathodic Peak Current (I_{pc}) [μA]
**PpIX**	+0.75	33.3	-1.01	-66.7
**EGCG**	+0.27	4.0	-0.98	-34.4
**PpIX/EGCG**	+0.67	59.4	-0.93	-48.5

References

[1] Y. Zu, Z. Gao, “Facile and controllable loading of single-stranded DNA on gold nanoparticles” *Anal Chem* 2009, *81*, 8523–8528.

[2] Z. Y. Lee, S. S. Raghavan, F. J. Ghadessy, Y. N. Teo, “Rapid and sensitive detection of acrylic acid using a novel fluorescence assay” *RSC Adv* 2014, *4*, 60216–60220.

[3] J. Li, J. Wang, J. Zhang, T. Han, X. Hu, M. M. S. Lee, D. Wang, B. Z. Tang, “A Facile Strategy of Boosting Photothermal Conversion Efficiency through State Transformation for Cancer Therapy” *Advanced Materials* 2021, *33*, 2105999.
